# Construction of heterometallic clusters with multiple uranium–metal bonds by using dianionic nitrogen–phosphorus ligands†

**DOI:** 10.1039/d0sc00389a

**Published:** 2020-03-13

**Authors:** Genfeng Feng, Karl N. McCabe, Shuao Wang, Laurent Maron, Congqing Zhu

**Affiliations:** State Key Laboratory of Coordination Chemistry, Jiangsu Key Laboratory of Advanced Organic Materials, School of Chemistry and Chemical Engineering, Nanjing University Nanjing China zcq@nju.edu.cn; LPCNO, CNRS & INSA, Université Paul Sabatier 135 Avenue de Rangueil Toulouse France laurent.maron@irsamc.ups-tlse.fr; State Key Laboratory of Radiation Medicine and Protection, School for Radiological and Interdisciplinary Sciences (RAD-X), Collaborative Innovation Center of Radiation Medicine of Jiangsu Higher Education Institutions, Soochow University Suzhou China

## Abstract

Compared with the prevalent metal–metal bond in transition metals, examples of the actinide–metal bond in heterometallic clusters are rare. Herein, a series of heterometallic clusters with multiple uranium–metal bonds has been prepared based on two newly synthesized nitrogen–phosphorus ligands **L1** {O[(CH_2_)_2_NHP(^i^Pr)_2_]_2_} and **L2** {[CH_2_O(CH_2_)_2_NHP(^i^Pr)_2_]_2_}. Different P–P distances, 6.069 and 4.464 Å, are observed in the corresponding uranium complexes **1** {O[(CH_2_)_2_NP(^i^Pr)_2_]_2_UCl_2_} and **2** {[CH_2_O(CH_2_)_2_NP(^i^Pr)_2_]_2_UCl_2_}, respectively, and lead to the different coordination modes with transition metals. The reactions of zero-valent group 10 metal compounds with complex **1** generate heterometallic clusters (**3-U2Ni2** and **4-U2Pd2**) featuring four uranium–metal bonds; whereas reactions with **2** afford one-dimensional metal-chain **5-(UNi)n**, bimetallic species **6-UPd**, and a tri-platinum bridged diuranium molecular cluster **7-U2Pt3**. Complex **5-(UNi)n** represents the first infinite chain containing the U–M bond and **7-U2Pt3** is the first species with multiple U–Pt bonds. This study further highlights the important role of ligands in the construction of multiple uranium–metal bonds and may allow the synthesis of novel d–f heterometallic clusters and the investigation of their applications in catalysis and small-molecule activation.

## Abstract 2

Compared with the prevalent metal–metal bond in transition metals, examples of the actinide–metal bond in heterometallic clusters are rare.

## Graphical abstract

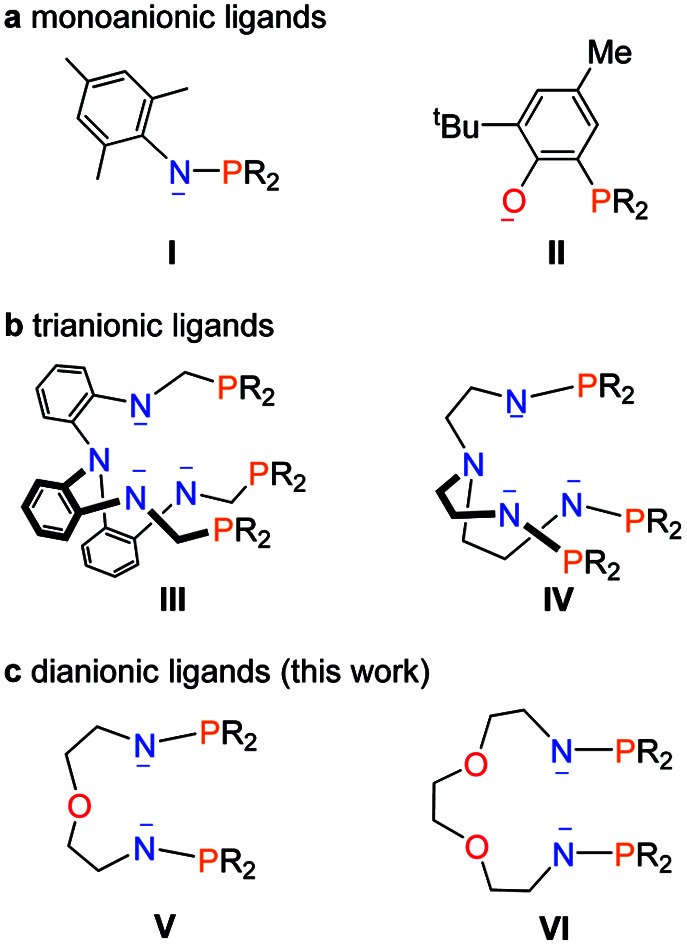

## Introduction

Multimetallic clusters containing metal–metal bonds have attracted many theoreticians and experimentalists due to their fascinating structures and potential applications in catalysis and activation of small molecules.^1^ Clusters with metal–metal bonds involving transition metals or even main-group metal elements have been extensively reported in recent decades.^2^ However, the synthesis of multimetallic clusters with direct bonding between f-block metals, especially actinide metals, and transition metals still lag far behind and are probably hindered by synthetic difficulties. Uranium has potential applications in catalysis and energy,^3^ and the heterometallic clusters featuring direct uranium-transition metal bonds are of particular interest due to the multimetallic synergistic effects from different metals.^4^

Remarkable progress concerning U–M bonds has been achieved by theoretical investigations,^5^ but the construction of U–M bonds remains a challenging project. The first complex containing a uranium–transition metal bond was produced by a salt elimination reaction and reported in 1987 by Sternal and Marks.^6^ Subsequently, the research into uranium–metal bonds went into a period of inactivity until in 2000, when the interaction of iron with uranium in ferrocenophane systems was reported by the groups of Ephritikhine and Diaconescu.^7^ In 2008, the Arnold group reported U–Al/Ga bonds based on a U(iii) metallocene species,^8^ and in the same period, Liddle and co-workers reported a series of complexes featuring U–M (M = Ga, Re, Ru, Co) bonds using a tris(2-aminoethyl)amine (Tren) ligand.^9^ These reports not only enriched the chemistry of uranium–metal bonds but also inspired its continuing development.^10^ Almost all of these examples however contain only a single U–M bond.

**Fig. 1:**
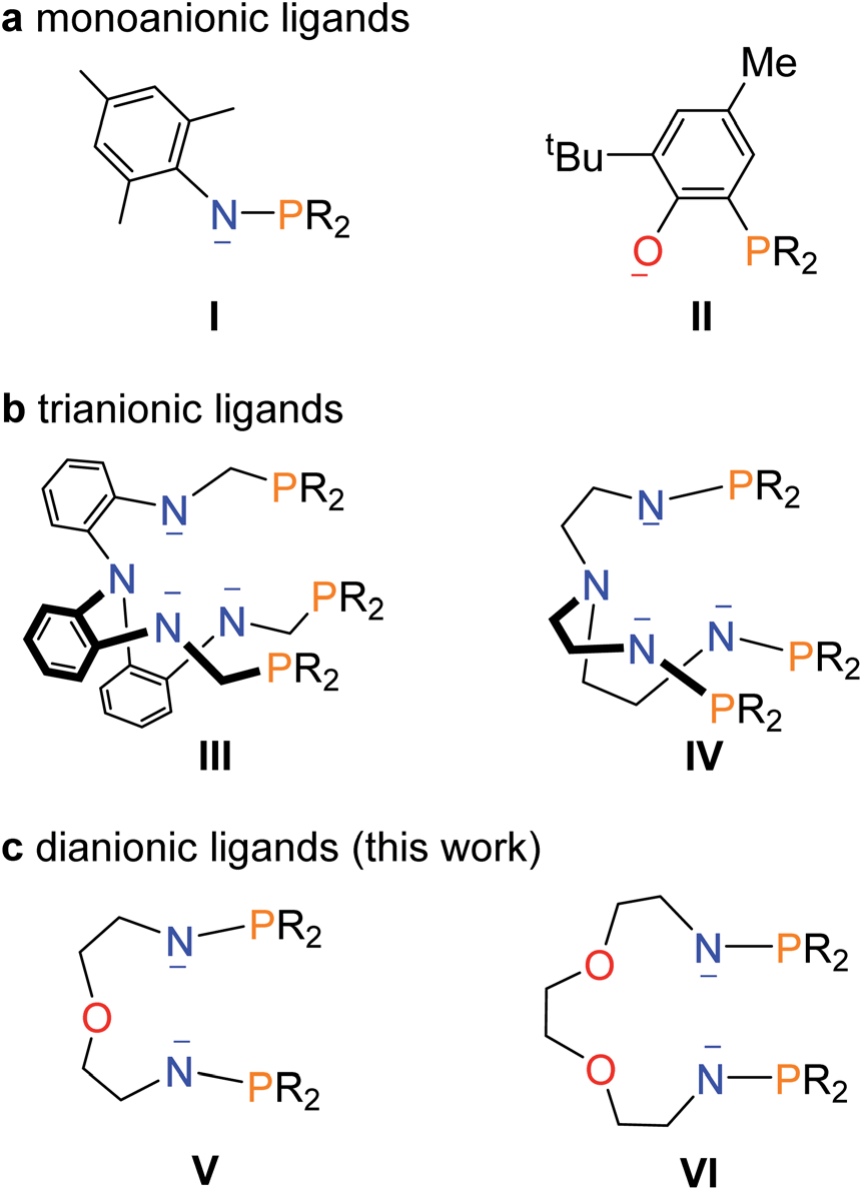
Representative ligands used in the construction of uranium–metal bonds.

Ligands play an important role in the stabilization of uranium–metal bonds. Two kinds of monoanionic ligands, phosphinoamide and phosphine-substituted aryloxide (**I** and **II** in Fig. 1), were used to synthesize complexes with U–M (M = Co, Rh, Ni, Pd, Pt, and Mo) bonds by the groups of Thomas, Bart, Arnold, and Liddle.^11^ However, at least threefold monoanionic ligands are used to stabilize a U–M bond in these species. Arnold and co-workers reported a trianionic ligand (**III** in Fig. 1) to stabilize a U–Co bond *via* a novel photolytic synthetic method.^12^ Recently, we found that a trianionic heptadentate N_4_P_3_ scaffold with three rigid N–P units (**IV** in Fig. 1) could be used to synthesize heterometallic clusters with multiple U–Ni bonds and two U–Rh triple bonds.^13^

This finding raises a question of whether dianionic ligands can be used to stabilize U–M bonds. Herein we report the synthesis and characterization of a series of heterometallic molecular clusters with multiple U–M (M = Ni, Pd, and Pt) bonds based on two newly synthesized dianionic ligands with rigid N–P units (**V** and **VI** in Fig. 1).

## Results and discussion

### Synthesis and structural characterization

**Scheme 1:**
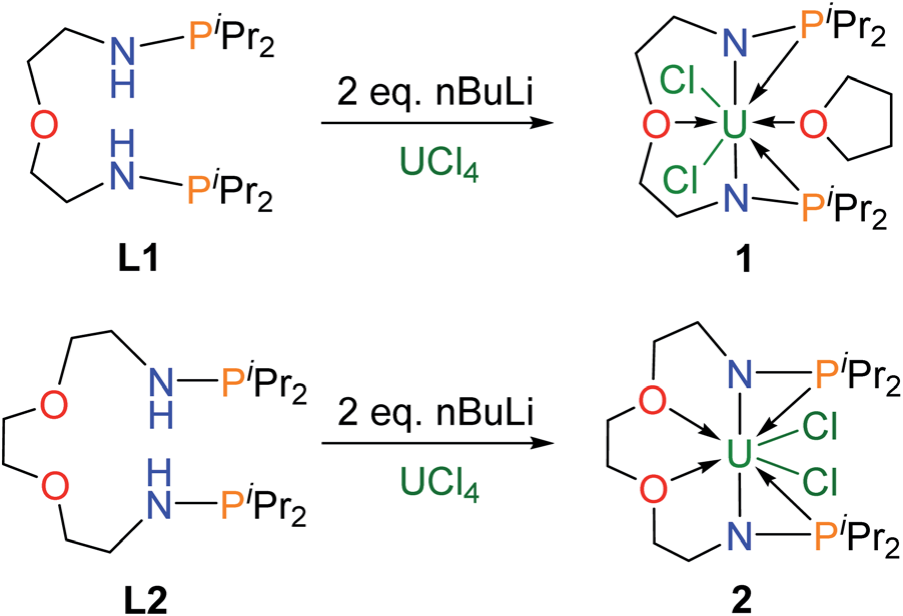
Preparation of the uranium precursors **1** and **2**.

Dianionic ligands (**L1** and **L2**) are easily prepared from the reactions of 2,2′-oxydiethylamine or 2,2′-(ethylenedioxy)diethylamine, respectively, with chlorodiiso-propylphosphine in the presence of triethylamine (see the ESI† for details). Ligand **L1** was first deprotonated with 2 equivalents of *n*-BuLi at −30 °C (Scheme 1) and after treatment of the resulting yellow solution with UCl_4_ at rt overnight, complex **1** was isolated as a gray-green solid in 58% yield after recrystallization from toluene at −30 °C.

Under the same conditions, complex **2** was prepared and isolated as green crystals in 65% yield from the reaction of ligand **L2** with UCl_4_ (Scheme 1). The ^1^H NMR spectra of complexes **1** and **2** show a broad range of peaks from +70 to −60 ppm and from +190 to −80 ppm (Fig. S7 and S8†), respectively. These ^1^H NMR spectra suggest that both **1** and **2** have a two-fold symmetry in solution. However, due to the paramagnetic properties of U(iv), no phosphorus signal was observed in the ^31^P NMR spectra ranging from +1000 to −1000 ppm. This phenomenon has been previously reported for actinide complexes.^11*b*,12^

**Fig. 2:**
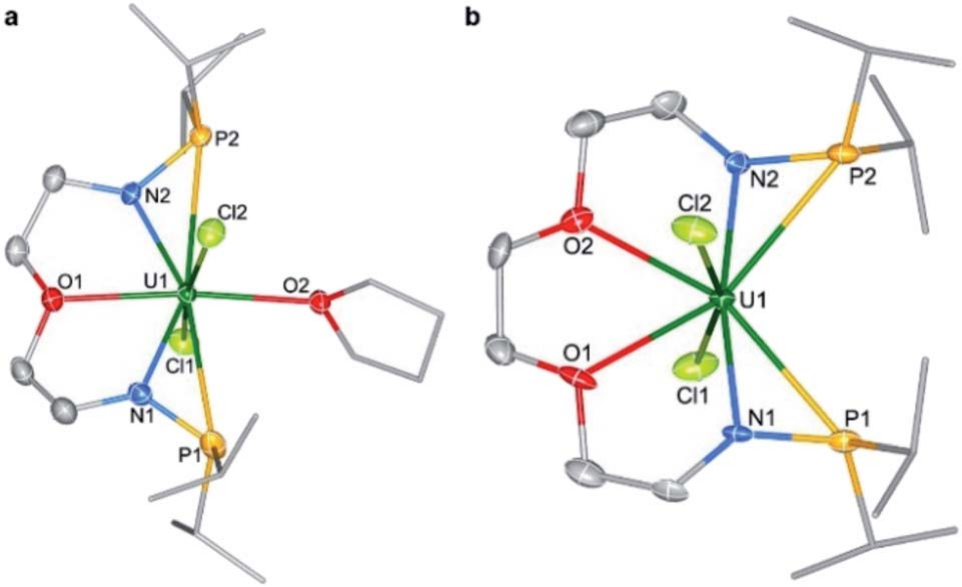
Molecular structures of **1** (a) and **2** (b). Thermal ellipsoids are drawn at 50% probability. Hydrogen atoms are omitted for clarity. Selected bond distances (Å) for **1**: U1–N1 2.245(5), U1–N2 2.227(5), U1–O1 2.485(5), U1–O2 2.485(5), U1–P1 3.075(2), U1–P2 3.047(2), U1–Cl1 2.644(2), and U1–Cl2 2.665(2); for **2**: U1–N1 2.245(6), U1–N2 2.235(5), U1–O1 2.542(5), U1–O2 2.541(5), U1–P1 2.882(2), U1–P2 2.894(2), U1–Cl1 2.688(2), and U1–Cl2 2.685(2).

Single crystals of complexes **1** and **2** suitable for X-ray diffraction were both grown in toluene at −30 °C. As illustrated in Fig. 2, the uranium centers of these species are eight-coordinated with two N atoms, two O atoms, two P atoms, and two Cl atoms. The successful assembly of complex **2** with a longer and more flexible ligand may result from the strong interaction between the O atoms and the U center. The U–N, U–O, and U–Cl bond distances in **1** and **2** are comparable, but the U–P bond lengths in complex **2** (2.882(2) and 2.894(2) Å) are clearly shorter than the corresponding U–P bonds in complex **1** (3.075(2) and 3.047(2) Å). The most significant difference between these uranium precursors is that the P1⋯P2 distance in complex **1** is 6.069 Å, whereas it is only 4.464 Å in complex **2** (Fig. 2). The different P–U–P angles in complex **1** (165.03(5)°) and complex **2** (100.99(6)°) were also observed. These geometries play key roles in the coordination modes upon reactions with group 10 metals.

**Scheme 2:**
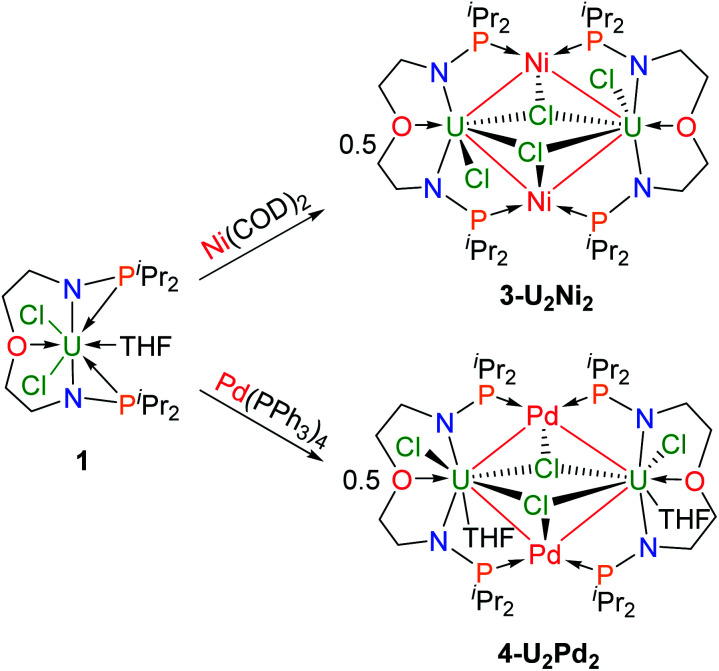
Synthesis of heterometallic clusters with multiple U–Ni (**3-U2Ni2**) and U–Pd (**4-U2Pd2**) bonds.

We attempted to react complex **1** with zero-valent group 10 metal compounds. Treatment of **1** with one equivalent of Ni(COD)_2_ (COD = cyclooctadiene) in THF at rt overnight afforded a dark solution (Scheme 2), from which complex **3-U2Ni2** was isolated as a black crystalline solid after standing at −30 °C. Other zero-valent group 10 metals were also used in this reaction. Orange-red microcrystals of **4-U2Pd2** were obtained from the reaction of complex **1** with an equimolar amount of Pd(PPh_3_)_4_ in THF (Scheme 2). We also attempted to prepare a platinum congener through the reaction of complex **1** with Pt(PPh_3_)_4_ or Pt(COD)_2_, but only an unidentified product was formed in an insoluble suspension, which could not be analyzed by ^1^H NMR. Although we examined many different conditions and methods for the growth of crystals, no single crystals suitable for X-ray crystallographic analysis were obtained. Therefore, we are unable to judge whether a similar cluster with multiple U–Pt bonds was formed. The ^1^H NMR spectrum of complex **3-U2Ni2** suggests its two-fold symmetry with ten peaks in the range of +70 to −40 ppm (Fig. S9†). However, ^1^H NMR of **4-U2Pd2** gave no signals even though different deuterated solvents were used, due to its extremely low solubility after precipitation or crystallization. The purities of **4-U2Pd2** were further confirmed by elemental analysis and X-ray powder diffraction (Fig. S10†). The complex **4-U2Pd2** is the first example of a palladium-bridged uranium dimer.

**Fig. 3:**
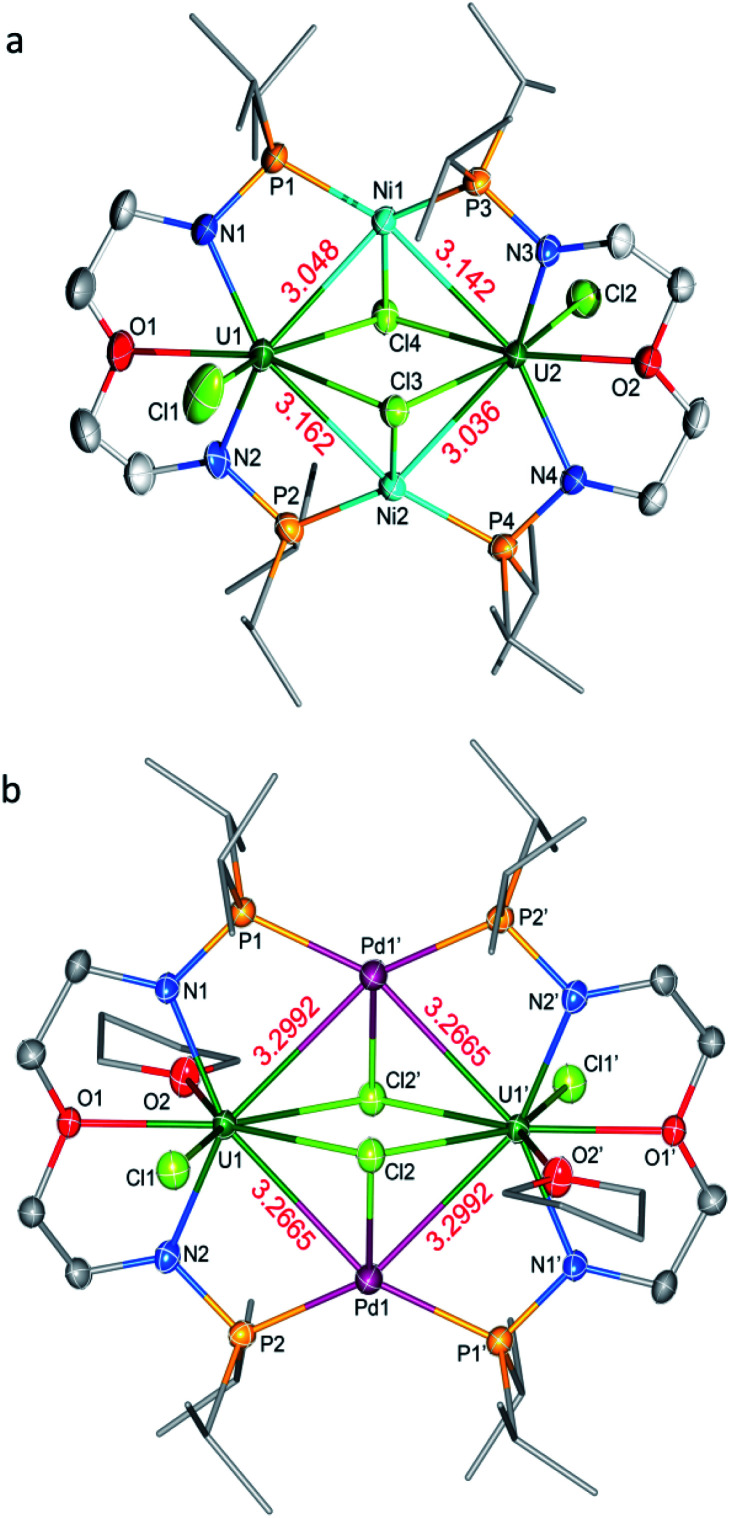
Molecular structures of **3-U2Ni2** (a) and **4-U2Pd2** (b). Thermal ellipsoids are drawn at 50% probability. Hydrogen atoms are omitted for clarity. THF molecules in the lattice of **4-U2Pd2** are also omitted. Selected bond distances (Å) for **3-U2Ni2**: U1–Ni1 3.048(1), U1–Ni2 3.162(1), U2–Ni1 3.142(1), U2–Ni2 3.036(1), U1–N1 2.243(7), U1–N2 2.248(7), U1–O1 2.532(7), U1–Cl1 2.633(3), U1–Cl3 2.867(2), U1–Cl4 2.870(2), U2–N3 2.256(7), U2–N4 2.252(7), U2–O2 2.502(6), U2–Cl2 2.653(2), U2–Cl3 2.815(2), and U2–Cl4 2.835(2); for **4-U2Pd2**: U1–Pd1 3.2665(6), U1–Pd1′ 3.2992(6), U1–N1 2.246(5), U1–N2 2.271(5), U1–O1 2.520(4), U1–O2 2.757(4), U1–Cl1 2.6928(16), U1–Cl2 2.9229(15), and U1–Cl2′ 2.8339(16).

The molecular structures of **3-U2Ni2** and **4-U2Pd2** in the solid-state were determined by single-crystal X-ray diffraction (Fig. 3). The crystal systems and space groups of **3-U2Ni2** and **4-U2Pd2** are monoclinic *P*2_1_/*n* and orthorhombic *Pccn*, respectively. The uranium centers in **3-U2Ni2** employ an eight-coordinated geometry with two Ni atoms, three Cl atoms, two N atoms and one O atom. The three donor atoms (N1, N2, and O1) from the ligand with U are coplanar. The coordination environment of uranium in **4-U2Pd2** is similar to that in **3-U2Ni2** except for the additional coordinated O atom from THF. The Ni and Pd atoms adopt almost the same coordination geometry with two U atoms, two P atoms, and one Cl atom. Interestingly, these clusters have similar M–U–M angles, 86.86(3)° in **3-U2Ni2** and 87.551(14)° in **4-U2Pd2**. The most remarkable feature of these species is the two uranium atoms bridged by two transition metals to form heterometallic clusters with four U–M bonds. In complex **3-U2Ni2**, the four U–Ni bond lengths were between 3.036(1) and 3.162(1) Å, which are 10% longer than the sum of the covalent single-bond radii of uranium and nickel (2.80 Å).^14^ The formal shortness ratio (FSR),^15^*i.e.* the ratio of the M–M bond length to the sum of the covalent atomic radii of the two metals, has been widely used to assess the bonding between two metals. The FSR values for the U–Ni bonds in **3-U2Ni2** range from 1.08 to 1.13, which are close to the FSR values of actinide–metal bonds reported in Th–Ni, U–Co and Th–Co bimetallic complexes, which range from 1.02 to 1.12.^11*a*,12,16^ However, the FSR values of U–Ni bonds in **3-U2Ni2** are slightly larger than the 0.90–0.91 in the complexes XU(μ-OAr^P^-1κ^1^O,2κ^1^P)_3_Ni (X = I, F, OSiMe_3_) reported by Arnold and co-workers.^11*b*^

The complex **4-U2Pd2** is the first reported heterometallic cluster with multiple U–Pd bonds (Fig. 3b). This cluster crystallizes in an orthorhombic space group *Pccn* with two centrosymmetric ligand chelated U–Pd moieties. The U–Pd bond lengths, 3.2665(6) and 3.2992(6) Å, are longer than the sum of the covalent single-bond radii of uranium and palladium (2.90 Å).^14^ The FSR values of U–Pd bonds in **4-U2Pd2** are 1.13 and 1.14, which are also larger than 0.93 in the complex IU(μ-OAr^P^-1κ^1^O,2κ^1^P)_3_Pd reported by Arnold and co-workers.^11*b*^ In addition, the FSR values for the U–Pd bonds in **4-U2Pd2** are slightly larger than those of U–Ni bonds in **3-U2Ni2**, which is probably due to the coordinated THF reducing the electronic accepting tendency of uranium(iv) from Pd(0).

**Scheme 3:**
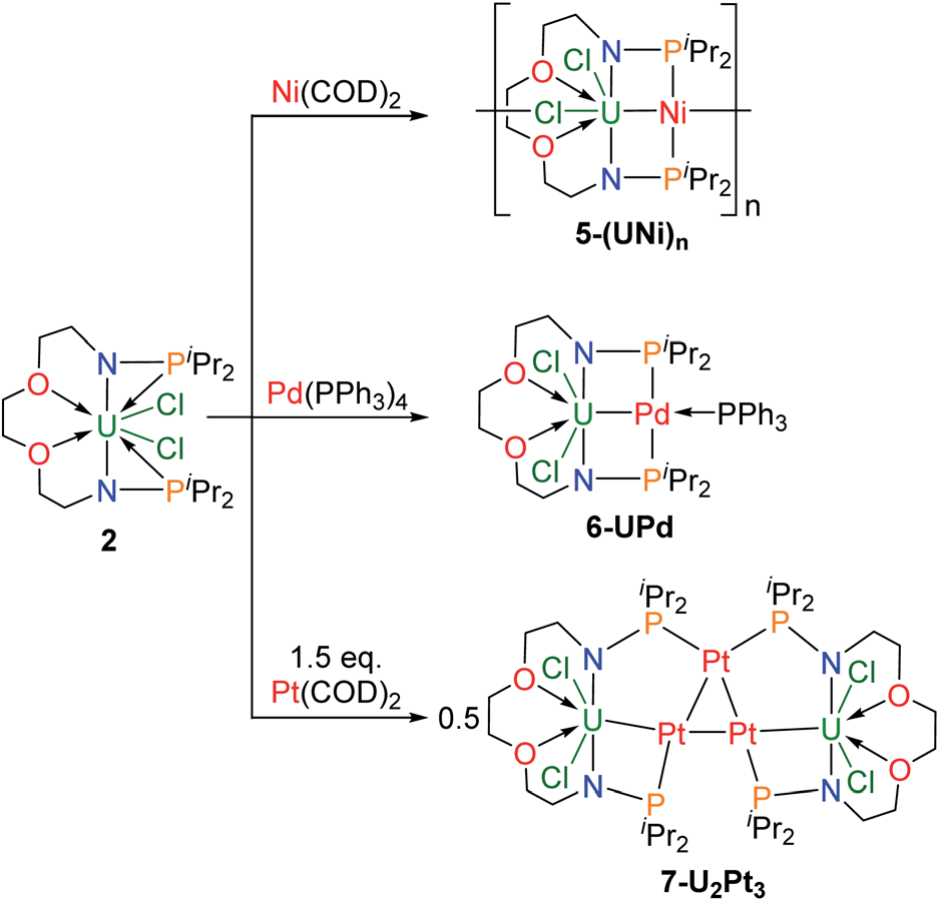
Synthesis of heterometallic clusters, **5-(UNi)n**, **6-UPd**, and **7-U2Pt3**.

In order to compare the effects of ligands on different coordination modes, the reactions of complex **2** with zero-valent transition metals were also explored (Scheme 3). The addition of one equivalent of Ni(COD)_2_ to a solution in THF of complex **2** led to a color change from green to black, and isolation of the complex **5-(UNi)n** as black crystals in 68% yield at −30 °C. Orange crystals of **6-UPd** and black crystals of **7-U2Pt3** were obtained in 49% and 52% yields by the reactions of complex **2** with Pd(PPh_3_)_4_ or Pt(COD)_2_, respectively, under the same conditions (Scheme 3). The characterization of these heterometallic clusters by multi-NMR spectroscopy was hindered by their poor solubility in different deuterated solvents and thus elemental analysis and X-ray powder diffraction were used to verify the purity of their bulk samples (Fig. S11–S13†).

**Fig. 4:**
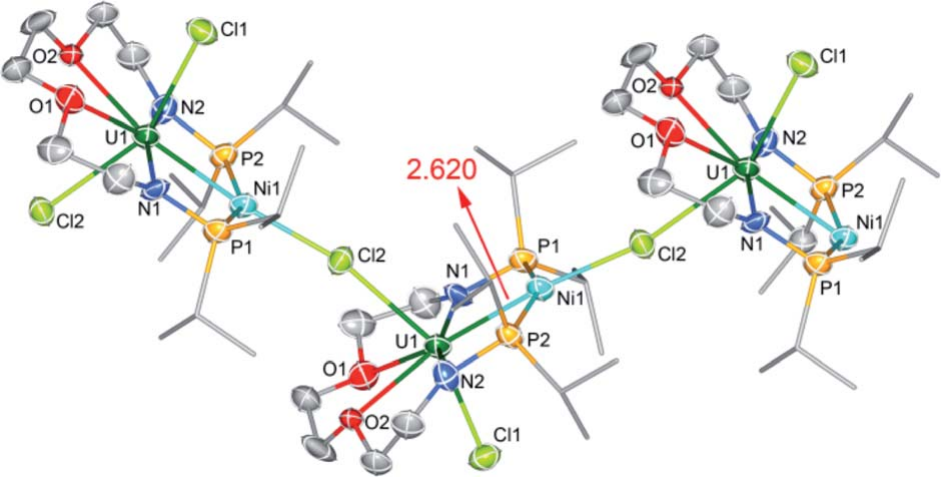
Molecular structures of **5-(UNi)n**. The infinite chain structure is sustained by Cl atoms. Thermal ellipsoids are drawn at 50% probability. Hydrogen atoms and THF molecules in the lattice are omitted for clarity. Selected bond distances (Å): U1–Ni1 2.620(3), U1–N1 2.241(16), U1–N2 2.236(17), U1–O1 2.548(15), U1–O2 2.523(13), U1–Cl1 2.711(5), and U1–Cl2 2.820(5).

The molecular structure of complex **5-(UNi)n** was confirmed by single crystal X-ray diffraction. As shown in Fig. 4, one nickel atom is ligated by two P atoms, probably due to the short separation of these P atoms (4.464 Å) in the precursor (**2**). Nickel is tetra-coordinated with two P atoms, one U atom, and one Cl atom from another unit and thus forms a one-dimensional molecular-chain. The uranium center in **5-(UNi)n** is seven coordinated with a pentagonal bipyramid geometry. The Cl–U–Cl and P–Ni–P angles are 153.83(17)° and 150.6(2)°, respectively. The U–Ni bond length in **5-(UNi)n** is 2.620(3) Å, which is much shorter than the sum of the covalent single-bond radii of uranium and nickel (2.80 Å),^14^ and thus the FSR value of the U–Ni bond in **5-(UNi)n** is only 0.94. The U–Ni bond length in **5-(UNi)n** is very close to those (2.520(1)-2.556(1) Å) found in the complexes XU(μ-OAr^P^-1κ^1^O,2κ^1^P)_3_Ni (X = I, F, OSiMe_3_).^11*b*^ The bond length of U–Ni (2.620(3) Å) in **5-(UNi)n** is significantly shorter than that observed in complex **3-U2Ni2** (3.097 Å on average), which denotes a stronger metal–metal bonding interaction between U and Ni in **5-(UNi)n**. Complex **5-(UNi)n** is the first example of a one-dimensional molecular-chain containing a U–M bond.

**Fig. 5:**
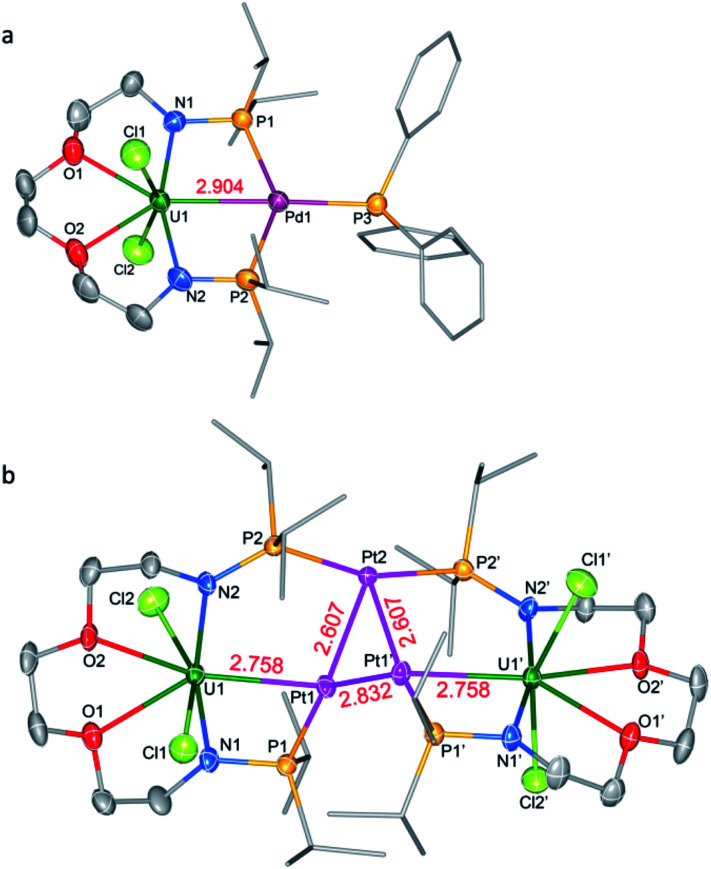
Molecular structures of bimetallic complex **6-UPd** (a) and heterometallic cluster **7-U2Pt3** (b). Thermal ellipsoids are drawn at 50% probability. Hydrogen atoms and THF molecules in the lattice are omitted for clarity. Selected bond distances (Å) for **6-UPd**: U1–Pd1 2.904(1), U1–N1 2.222(4), U1–N2 2.216(4), U1–O1 2.481(3), U1–O2 2.510(3), U1–Cl1 2.700(1), and U1–Cl2 2.701(1); for **7-U2Pt3**: U1–Pt1 2.758(1), Pt1–Pt2 2.607(1), Pt1–Pt1′ 2.832(1), Pt2–Pt1′ 2.607(1), U1–N1 2.286(5), U1–N2 2.343(5), U1–O1 2.600(4), U1–O2 2.541(4), U1–Cl1 2.673(2), and U1–Cl2 2.687(2).

Complex **6-UPd** is a typical bimetallic species with a U–Pd bond and a palladium which is terminally blocked by a PPh_3_ molecule (Fig. 5a). **6-UPd** crystallizes in a triclinic space group P-1. The Cl–U–Cl and P–Pd–P angles are 155.73(5)° and 139.43(4)°, respectively. In addition, the geometries of uranium and palladium in **6-UPd** are similar to the corresponding geometries in **5-(UNi)n**. The U–Pd bond length (2.904(1) Å) in complex **6-UPd** is similar to the sum (2.90 Å) of the covalent single-bond radii of uranium and palladium and thus the FSR of this U–Pd bond is 1.0. Although the U–Pd bond length in **6-UPd** is longer than the single example of a U–Pd bond (2.686(2) − 2.694(1) Å, FSR = 0.93) in the complex IU(μ-OAr^P^-1κ^1^O,2κ^1^P)_3_Pd reported by Arnold and co-workers,^11*b*^ it is still about 0.4 Å shorter than the U–Pd bond found in **4-U2Pd2**, which is 3.283 Å on average.

In contrast to molecules such as **5-(UNi)n** and **6-UPd** in which only one transition metal was bound between two P atoms, the triplatinum-bridged diuranium cluster, **7-U2Pt3**, represents an unexpected result which was not affected by the use of less Pt(COD)_2_. As shown in Fig. 5b, the cluster possesses a folded structure with Pt2 as the node and three platinum atoms constitute a triangle of 3 Pt–Pt bonds. The coordinated environment of the uranium center in **7-U2Pt3** is similar to that in **5-(UNi)n** and **6-UPd**, whereas each Pt has a roughly tetrahedral geometry. The centrosymmetric *C*2/*c* space group of **7-U2Pt3** exhibits identical lengths (2.758(1) Å) for two U–Pt bonds. The U–Pt distance is shorter than the sum of the covalent single-bond radii of uranium and platinum (2.93 Å) and thus the FSR value of this U–Pt bond is 0.94, which is comparable to the only published example of a U–Pt bond in the complex IU(μ-OAr^P^-1κ^1^O,2κ^1^P)_3_Pt (2.706(1)–2.709(1) Å, FSR = 0.92).^11*b*^ It is also shorter than the Th–Pt bond distance (2.984(1) Å) in the complex (η^5^-C_5_Me_5_)_2_Th(μ-PPh_2_)_2_Pt(PMe_3_) overlooking the small radius difference between Th and U atoms.^17^ Two distinct Pt–Pt bond lengths (2.607(1) and 2.832(1) Å) were observed among the three platinum atoms. They are both longer than the sum of the covalent single-bond radii of two platinum atoms (2.46 Å), and this implies a weak Pt–Pt interaction. A triangular trimetal unit is a structure long known in group 10 and 11 metals with a d^10^ electron configuration and its formation could be described as constituting d^10^–d^10^ weak interactions.^18^ The complex **7-U2Pt3** is the first example of the d–f heterometallic cluster with more than one U–Pt bond.

### Magnetic susceptibility and UV-Vis/NIR spectroscopy

**Fig. 6:**
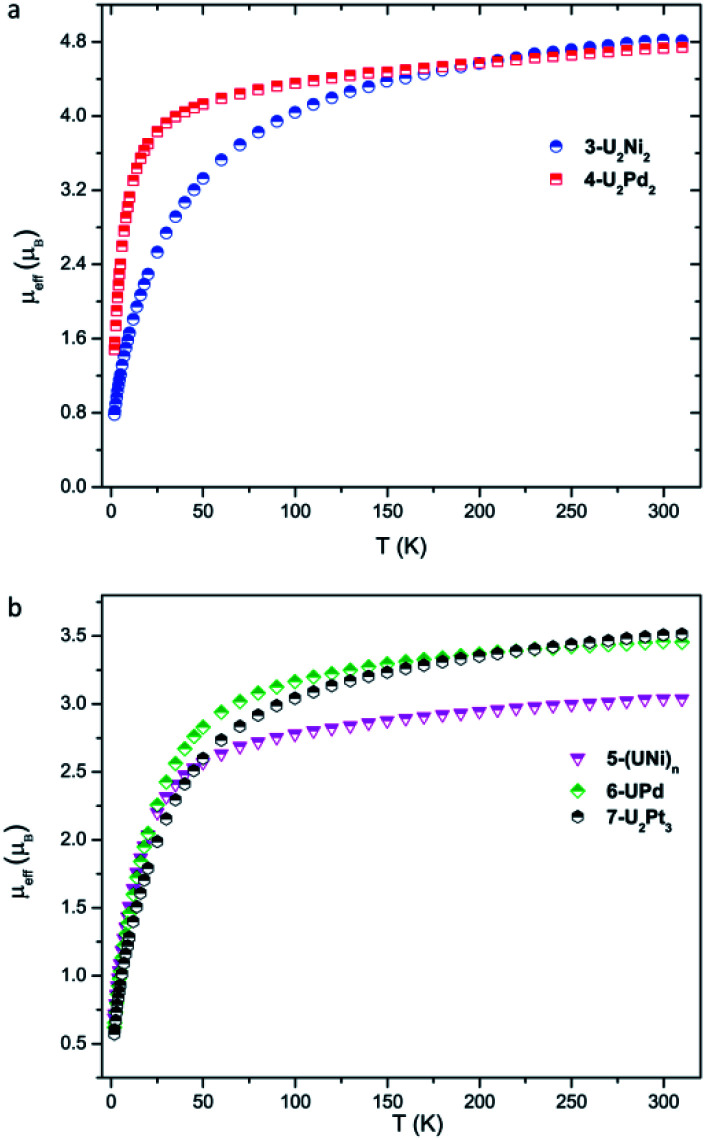
Variable-temperature effective magnetic moment data. (a) Magnetic moment *per molecule* for **3-U2Ni2** and **4-U2Pd2**. (b) Magnetic moment *per uranium ion* for **5-(UNi)n**, **6-UPd**, and **7-U2Pt3**.

Variable-temperature magnetic data for **3-U2Ni2**, **4-U2Pd2**, **5-(UNi)n**, **6-UPd**, and **7-U2Pt3** in the solid state were collected using a superconducting quantum interference device (SQUID) (Fig. 6). The effective magnetic moments for **3-U2Ni2** and **4-U2Pd2** at 300 K are 4.81 and 4.74 *μ*_B_ per molecule, respectively, which are lower than the theoretical value for two independent f^2^ uranium ions in the ^3^H_4_ ground state (5.06 *μ*_B_). This phenomenon has been observed in previously reported U(iv) complexes and is thought to be due to the quenching of spin–orbit coupling.^9*a*,19^

The magnetic moments of complexes **3-U2Ni2** and **4-U2Pd2** decline slowly from room temperature, then fall, reaching the values of 0.78 *μ*_B_ and 1.45 *μ*_B_ per molecule (two U ions) at 1.8 K with a trend to zero, indicating a magnetic singlet at low temperature or anti-ferromagnetic coupling. However, the magnetic moment of **4-U2Pd2** drops sharply below 25 K, whereas the magnetic moment of **3-U2Ni2** declines much more gradually and starts to decline at a higher temperature (150 K). It appears that low-lying magnetic states of **4-U2Pd2** are accessible at temperatures below 25 K while the energetically higher magnetic states of **3-U2Ni2** slowly become thermally accessible with increasing temperature. Since it is coordinated by an additional THF molecule, **4-U2Pd**_2_ retains a higher moment for a longer time than is usual for U(iv) complexes, which has been observed in complexes bearing strong donor ligands.^20^ At 1.8 K, the magnetic moment of **4-U2Pd2** is about 1.03 *μ*_B_ per U ion. Although this value is slightly higher than that of the common U(iv) complexes, it is still close to that of the U(iv) complexes, [Cp*_2_Co][U(O) (NR_2_)_3_], [{U(Tren^TIPS^) (AsK_2_)}_4_] and [((^Ad,Me^ArO)_3_tacn)U(OH)], reported by the groups of Liddle, Hayton and Meyer.^21^ These results confirm the U(iv) formulation in **3-U2Ni2** and **4-U2Pd2**.

The +IV oxidation state assignments of uranium for complexes **5-(UNi)n**, **6-UPd**, and **7-U2Pt3** were also confirmed from their similar temperature dependencies (Fig. 6b). In the temperature range of 300–50 K, complexes **5-(UNi)n**, **6-UPd**, and **7-U2Pt3** display *μ*_eff_ values in small ranges of 3.03–2.51 *μ*_B_, 3.45–2.83 *μ*_B_, and 3.52–2.60 *μ*_B_ per U ion, respectively. The quenching of spin–orbit coupling for complex **5-(UNi)n** with a short U–Ni bond perhaps results in it retaining a lower *μ*_eff_ value than the other two complexes above 50 K. As the temperature is lowered, the magnetic moments of **5-(UNi)n**, **6-UPd**, and **7-U2Pt3** decrease precipitously toward zero, reaching 0.69, 0.62, and 0.57 *μ*_B_ at 1.8 K, respectively.

**Fig. 7:**
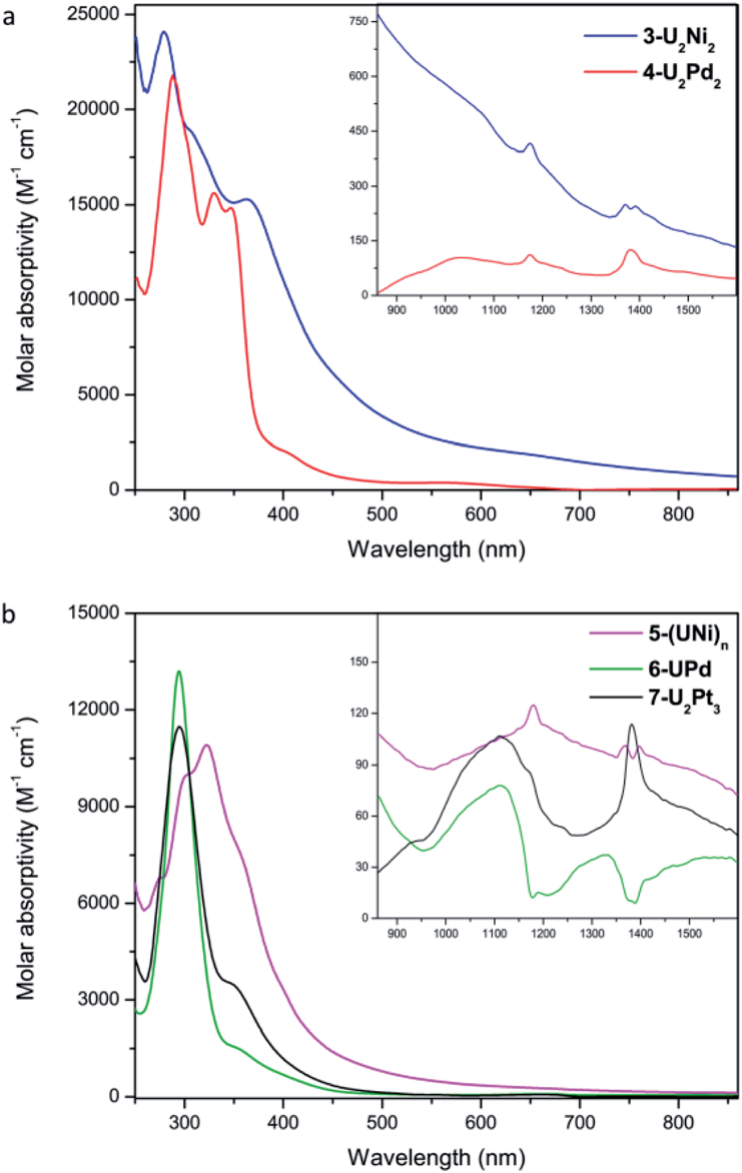
UV-Vis absorption spectra of **3-U2Ni2** and **4-U2Pd2** (a), **5-(UNi)n**, **6-UPd**, and **7-U2Pt3** (b) measured in THF at rt. Inset: near infrared absorption spectra.

The UV-Vis-NIR electronic absorption spectra of these heterometallic molecular clusters were recorded in THF at rt (Fig. 7). The complexes **3-U2Ni2** and **4-U2Pd2** display two intense absorption peaks at 280 nm and 288 nm, respectively, with a shoulder at 359 nm for **3-U2Ni2** and two peaks at 330 nm and 347 nm for **4-U2Pd2**. These intense absorptions may be assigned to charge-transfer bands. In the NIR region, a similar, but weak absorption behavior is observed for **3-U2Ni2** and **4-U2Pd2**, which is characteristic for f*–*f transitions with small molar extinction coefficients. Complex **5-(UNi)n** exhibits broad and intense charge-transfer bands in the UV-Vis region with a peak centered at 323 nm (Fig. 7b). In the NIR region, three peaks at 1180, 1370, and 1396 nm were observed, which are similar to those in the spectrum of complex **3-U2Ni2** at 1175, 1372, and 1390 nm. These weak absorptions (*ε* < 150 M^−1^ cm^−1^) are attributed to f–f transitions expected for U(iv) complexes. Complexes **6-UPd** and **7-U2Pt3** exhibit charge-transfer absorptions at the same position, 295 nm with shoulders at about 350 nm, and they also have similar weak absorption bands from 800 to 1600 nm, which could be attributed to f–f transitions of U(iv). The intense and weak absorptions observed for these complexes in the UV-Vis and NIR regions compare well with those in the reported U(iv) complexes and support the presence of U(iv) in all these new heterometallic clusters.^22^ However, the assignment of U–M absorptions in the UV-Vis-NIR absorption spectra is impossible, perhaps due to the low energy of U–M bonds.

### Computational investigation

**Fig. 8:**
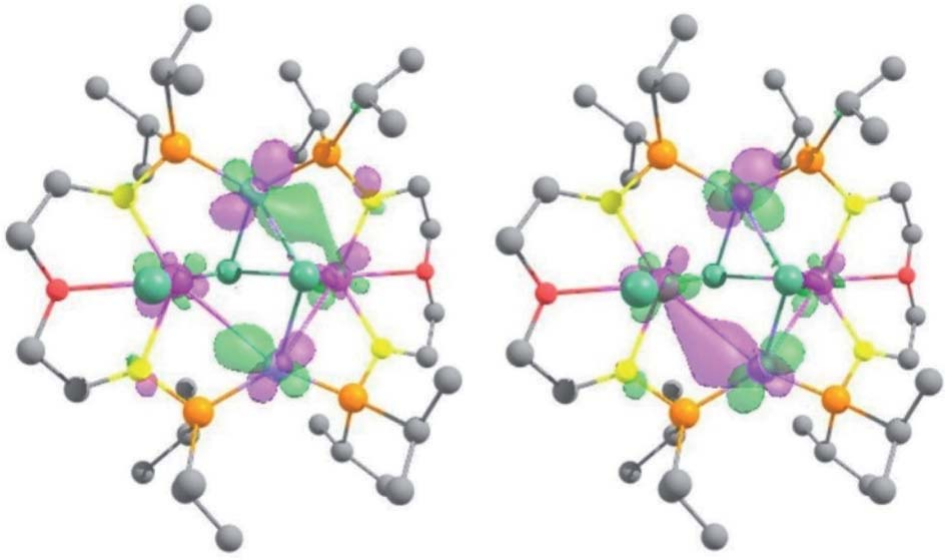
Representation of the HOMO and HOMO−1 of complex **3-U2Ni2**. The contour plot is set to 0.03.

In order to gain insights into the nature of the U–M interactions, DFT calculations (B3PW91) were carried out on the complexes **3-U2Ni2**, **4-U2Pd2**, **5-(UNi)n**, **6-UPd** and **7-U2Pt3**. The optimized geometries of these five complexes compare well with the experimental data (Figs. S26, S28, S30, S32 and S35†). In **3-U2Ni2**, the U–Ni distances are computed to be 3.20 Å (in excellent agreement with the experimental ones ranging between 3.04 and 3.16 Å) and the U–Cl (bridging) is 2.80 Å (2.81 Å experimentally). Even though the U–Ni distances are long, the HOMO and HOMO−1 clearly describe a bonding interaction between U and Ni (Fig. 8). This is further illuminated by a natural bonding orbital (NBO) analysis. Indeed, two U–Ni bonds that are strongly polarized toward Ni (89–90%) were located. These bonds involve a hybrid sdf orbital on U (12% s, 28% d, and 53% f) and a pure d orbital on Ni (99% d). The strong polarization of this bond is reflected in the Wiberg bond index (WBI) that is 0.4 for the U–Ni (for comparison, the U–Cl index is 0.6 and that of U–N is 0.8). Therefore, consistent with the long distance, the U–Ni interaction exists but is not strongly covalent, reflecting the mismatch between the energies of the atomic orbitals on U and Ni that are involved in the interaction. Finally, the unpaired density plot (Fig. S27†) clearly demonstrates that the two uranium centers have an oxidation state of +IV and therefore the two Ni atoms are zero-valent. In complex **4-U2Pd2**, the U–Pd distances compare well with the experimental ones (3.20–3.40 Å *vs.* 3.26/3.29 Å) as well as the U–Cl ones (2.70–2.90 Å both computationally and experimentally). As for complex **3-U2Ni2**, MOs (see Fig. S33 in the ESI†) are displaying some U–Pd bonding interactions between d orbitals on Pd and f orbitals on U. The NBO analysis indicates that the U–Pd WBI is also small (0.2 and 0.3) in line with a donor–acceptor interaction as observed in **3-U2Ni2**. The donation occurs from a filled d orbital on Pd onto an empty orbital on U. This together with the unpaired spin density (Fig. S34†) is consistent with two uranium centers with an oxidation state +IV and a zero-valent Pd.

For complex **6-UPd**, the U–Pd distance is perfectly reproduced by our computational method (2.90 Å). Quite unexpectedly, with such a short U–Pd distance, no bonding interaction was found on the molecular orbitals unlike what was found for the complex **3-U2Ni2**. The same holds true for the NBO analysis. The U–Pd WBI is however equal to 0.4, similar to what was found for the U–Ni interactions in **3-U2Ni2**. At the second-order donor–acceptor NBO level, a donation from occupied d orbitals on Pd to an empty hybrid sdf orbital on uranium (roughly 50 kcal mol^−1^) and also a donation from the Pd–P bonds toward uranium are observed. The latter donation describes a kind of push–pull effect that enhances the U–Pd interaction. This difference in bonding seems to be associated with the ligand and more precisely to the presence of the phosphorus atoms. Indeed, the NBO analysis also clearly reveals some donation of the U–P bonds into the empty orbitals on Pd (mainly sp). The unpaired spin density plot (Fig. S29†) shows some residual spin density on the Pd in line with the latter donation but the complex should be mainly regarded as a U(iv) system and a Pd(0).

To further investigate this influence of the ligand, the complex **7-U2Pt3** was analyzed in the same way. The U–Pt bonds are computed to be 2.80 Å in excellent agreement with the experimental one (2.75 Å). The Pt–Pt distances in the triangular structure are also well reproduced (2.70 Å *vs.* 2.60 Å experimentally and 2.90 Å *vs.* 2.80 Å). Similar to complex **6-UPd**, no bonding U–Pt interaction could have been found in the molecular orbitals. However, at the NBO level, a strong polarization toward Pt (91%) in the U–Pt bond was found, that involves a hybrid spdf orbital on U (26% s, 12% p, 45% d, and 17% f) and a hybrid sd orbital on Pt (7% s and 93% d). Despite this strong polarization, the U–Pt WBI is 0.8 indicating a very important covalent contribution in the interaction. This reflects the better match between the energies of the atomic orbitals of U and Pt. As in **6-UPd**, the NBO analysis indicates some donation of the U–P bonds into the empty orbitals on Pt (mainly sp). This donation is lower and does not influence the nature of the U–Pt interaction, which is mainly controlled by the energy of the atomic orbitals. In the triangular Pt_3_ moiety, only donor–acceptor interactions are observed in line with d^10^–d^10^ interactions between Pt(0) and the associated WBI is in the range 0.2–0.3. The oxidation states were confirmed by analyzing the unpaired spin density plot (Fig. S31†) where the density is only located on each uranium center in line with two U(iv). Calculations were carried out on the monomer part of **5-(UNi)n**. Unlike all the other examples, the U–Ni distance is not correctly reproduced (3.20 *vs.* 2.62 Å) so that the bonding analysis would be meaningless. However, this means that the short U–Ni distance in **5-(UNi)n** is likely due to the formation of a polymeric structure.

## Conclusions

Two new dianionic ligands, each with two rigid N–P units, have been successfully designed and synthesized. They were used to construct a series of novel heterometallic molecular clusters with multiple uranium–metal bonds. Specifically, **5-(UNi)n** is the first example of one-dimensional molecular-chain containing U–M bonds, while **4-U2Pd2** and **7-U2Pt3** represent the first clusters containing multiple U–Pd and U–Pt bonds, respectively. This work not only demonstrates the great potential of the dianionic N–P ligands in the construction of uranium–metal bonds but also provides a new platform to construct d–f heterometallic clusters with metal–metal bonds. This may promote the utilization of these novel clusters in catalysis and activation of small molecules.

## Conflicts of interest

The authors declare no conflict of interest.

## Supplementary Material

SC-011-D0SC00389A-s001

SC-011-D0SC00389A-s002

## References

[cit1] (a) Adams R. D. and Cotton F. A., Catalysis by di- and polynuclear metal cluster complexes, Wiley-VCH, New York, 1998

[cit2] (b) Gonzalez-Moraga G., Cluster chemistry: introduction to the chemistry of transition metal and main group element molecular clusters, Springer-Verlag, Berlin, 1993

[cit3] Fox A. R., Bart S. C., Meyer K., Cummins C. C. (2008). Nature.

[cit4] Gambarotta S., Scott J. (2004). Angew. Chem., Int. Ed..

[cit5] Gagliardi L., Pyykkö P. (2004). Angew. Chem., Int. Ed..

[cit6] Sternal R. S., Marks T. J. (1987). Organometallics.

[cit7] Bucaille A., Le Borgne T., Ephritikhine M., Daran J. C. (2000). Organometallics.

[cit8] Minasian S. G., Krinsky J. L., Williams V. A., Arnold J. (2008). J. Am. Chem. Soc..

[cit9] Liddle S. T., McMaster J., Mills D. P., Blake A. J., Jones C., Woodul W. D. (2009). Angew. Chem., Int. Ed..

[cit10] Chi C., Wang J.-Q., Qu H., Li W.-L., Meng L., Luo M., Li J., Zhou M. (2017). Angew. Chem., Int. Ed..

[cit11] Napoline J. W., Kraft S. J., Matson E. M., Fanwick P. E., Bart S. C., Thomas C. M. (2013). Inorg. Chem..

[cit12] Ward A. L., Lukens W. W., Lu C. C., Arnold J. (2014). J. Am. Chem. Soc..

[cit13] Feng G., Zhang M., Shao D., Wang X., Wang S., Maron L., Zhu C. (2019). Nat. Chem..

[cit14] Pyykkö P., Atsumi M. (2009). Chem.–Eur. J..

[cit15] (a) Cotton F. A., Murillo C. A. and Walton R. A., Multiple Bonds Between Metal Atoms, Springer Science and Business Media, Inc., New York, 3rd edn, 2005

[cit16] Ritchey J. M., Zozulin A. J., Wrobleski D. A., Ryan R. R., Wasserman H. J., Moody D. C., Paine R. T. (1985). J. Am. Chem. Soc..

[cit17] Hay P. J., Ryan R. R., Salazar K. V., Wrobleski D. A., Sattelberger A. P. (1986). J. Am. Chem. Soc..

[cit18] Imhof D., Venanzi L. M. (1994). Chem. Soc. Rev..

[cit19] Lam O. P., Anthon C., Heinemann F. W., O'Connor J. M., Meyer K. (2008). J. Am. Chem. Soc..

[cit20] King D. M., McMaster J., Tuna F., McInnes E. J. L., Lewis W., Blake A. J., Liddle S. T. (2014). J. Am. Chem. Soc..

[cit21] Brown J. L., Fortier S., Lewis R. A., Wu G., Hayton T. W. (2012). J. Am. Chem. Soc..

[cit22] Castro-Rodríguez I., Meyer K. (2006). Chem. Commun..

