# Supplementary material for: Construction of heterometallic clusters with multiple uranium–metal bonds by using dianionic nitrogen–phosphorus ligands
Source: Chem Sci. 2020 Mar 13;11(29):7585–92. doi: 10.1039/d0sc00389a (PMC8152682; doi:10.1039/d0sc00389a)
Supplement: SC-011-D0SC00389A-s001 [file SC-011-D0SC00389A-s001.pdf]

Electronic Supplementary Material (ESI) for Chemical Science.
This journal is © The Royal Society of Chemistry 2020

Electronic Supplementary Information (ESI)

Construction of Heterometallic Clusters with Multiple Uranium-Metal Bonds by Dianionic Nitrogen-Phosphorus Ligands

Genfeng Feng,[a] Karl N. McCabe,[b] Shuao Wang,[c] Laurent Maron,*[b] and Congqing Zhu*[a]

[a] State Key Laboratory of Coordination Chemistry, Jiangsu Key Laboratory of Advanced Organic Materials, School of Chemistry and Chemical Engineering, Nanjing University, Nanjing, China.

[b] LPCNO, CNRS & INSA, Université Paul Sabatier, 135 Avenue de Rangueil, Toulouse, France.

[c] State Key Laboratory of Radiation Medicine and Protection, School for Radiological and interdisciplinary Sciences (RAD-X) and Collaborative Innovation Center of Radiation Medicine of Jiangsu Higher Education Institutions, Soochow University, Suzhou, China.

Content

1. Experimental Procedures……………………………………………………………………………..2
2. NMR spectra……………………………………………………………………………………………..5
3. Powder X-ray diffraction……………………………………………………………………………10
4. X-ray Crystallographic data…………………………………………………………………………12
5. Magnetic measurements data………………………………………………………37
6. Computational Section………………………………………………………………………………39
7. References……………………………………………………………………………………………….66

1. Experimental Procedures

All operations were carried out using an inert atmosphere glove box with dry argon (<1 ppm O_2/H_2O). Solvents were dried and deoxygenated before use through a commercial solvent drying system. Samples were carefully checked for purity and data reproducibility. All NMR spectra were recorded on Bruker AVQ-400 or DRX 500 spectrometers with tetramethylsilane (TMS) as the internal standard at room temperature. CHN microanalyses were performed on a Vario EL III elemental analyser at Shanghai Institute of Organic Chemistry, the Chinese Academy of Sciences, and the samples were treated under high vacuum for 24 hours at room temperature. The powder X-ray diffraction pattern (PXRD) measurements were obtained on a Philips X'pert MPD Pro X-ray diffractometer using Cu Kα radiation (λ = 0.15418 nm), and the X-ray tube was operated at 40 kV and 40 mA at room temperature. Magnetic susceptibility measurements on crystalline samples were carried out on a SQUID magnetometer at 0.1 T in the temperature range from 1.8 to 300 K. UV-Vis-NIR spectra were recorded on a Lambda 750 spectrometer at room temperature. $Pt(COD)_2$ was synthesized according to a published method.[S1]

L1 and **L2**. 2, 2'-Oxydiethanamine (1.00 g, 9.6 mmol) and Et_3N (9.70 g, 96.0 mmol) were dissolved in THF (150 mL). Chlorodiisopropylphosphine (2.93 g, 19.2 mmol) was then added dropwise and the mixture was stirred overnight at room temperature. Volatiles were removed in vacuo and the residual white solid was extracted with hexane. After filtration, the solvents were removed in vacuo leaving **L1** as a colorless oil (3.0 g 93%). ^{1}H NMR (500 MHz, C_6D_6, ppm): δ 3.26 (t, $^3J_{H-H}$ = 5.5 Hz, 4H, -OC*H₂*-), 3.10-3.07 (m, 4H, -C*H₂*NH-), 1.49–1.43 (m, 4H,-*CH*Me$_2$), 1.22–1.19 (m, 4H, -N*H*-), 1.06–1.00 (m, 24H, -C*H₃*). ^{31}P NMR (202 MHz, C_6D_6, ppm): δ 65.23. ^{13}C NMR (126 MHz, C_6D_6, ppm): δ 72.09 (d, $^3J_{C-P}$ = 7.6 Hz, -*C*H$_2$O-), 47.65 (d, $^3J_{C-P}$ = 31.5 Hz, -*C*H$_2$NH-), 25.71 (d, $^1J_{C-P}$ = 16.4 Hz, -*C*H(CH$_3$)$_2$-) 18.23, (d, $^2J_{C-P}$ = 26.5 Hz, -*C*H$_3$-), 16.36 (d, $^2J_{C-P}$ = 11.3 Hz, -*C*H$_3$-). The synthesis of ligand **L2** (91% yield) was accomplished by the same method except that 2, 2'-(ethylenedioxy)diethylamine was used. ^{1}H NMR (500 MHz, C_6D_6, ppm): δ 3.40 (s, 4H, -OC*H₂*-), 3.30 (t, $^3J_{H-H}$ = 5.5 Hz, 4H, -OC*H₂*CH$_2$NH-), 3.11–3.06 (m, 4H, -OCH$_2$C*H₂*NH-), 1.47–1.43 (m, 4H, -C*H*Me$_2$), 1.30–1.27 (m, 24H, -N*H*-), 1.05–0.99 (m, 24H, -C*H₃*). ^{31}P NMR (202 MHz, C_6D_6, ppm): δ 65.26. ^{13}C NMR (126 MHz, C_6D_6, ppm): δ 73.18 (d, $^3J_{C-P}$ = 8.8 Hz, -O*C*H$_2$CH$_2$NH-), 70.26 (s, -O*C*H$_2$-) 48.43 (d, $^2J_{C-P}$ = 31.5 Hz, -*C*H$_2$NH-) 26.49 (d, $^1J_{C-P}$ = 16.4 Hz, -*C*H(CH$_3$)$_2$) 19.03 (d, $^2J_{C-P}$ = 26.5 Hz, -*C*H$_3$-) 17.17 (d, $^2J_{C-P}$ = 11.3 Hz, -*C*H$_3$-).

1 and **2**. Ligand **L1** (336 mg, 1.00 mmol) was dissolved in 5 mL THF and pre-cooled at -30°C. n-BuLi (2.4 M, 0.83 mL, 2.00 mmol) was then added dropwise. Three hours later, UCl_4 (380 mg, 1.00 mmol) in 5 mL THF was added at -30°C. The reaction mixture warmed to room temperature and then stirred for 6 h. Volatile materials were removed under reduced pressure and the mixture was extracted with toluene. Gray-green crystals of **1** (415 mg, 58%) were isolated from toluene solution by storage at −30 °C for 24 h. Anal. Calcd (%) for $C_{20}H_{44}Cl_2N_2O_2P_2U$: C, 33.58; H, 6.20; N, 3.92. Found: C, 33.45; H, 6.30; N, 3.94. ^{1}H NMR (500 MHz, C_6D_6, ppm): δ 66.67 (s, 4H), 61.18 (s, 4H), 30.00 (s, 12H), 29.21 (s, 12H), -22.36 (s, 4H, THF), -47.72 (s, 4H), -53.20 (s, 4H, THF). The synthesis of complex **2** (65% yield) was accomplished by the same method except that ligand **L2** was used. Anal. Calcd (%) for $C_{18}H_{40}Cl_2N_2O_2P_2U$: C, 31.45; H, 5.87; N, 4.08. Found: C, 31.00; H, 6.19; N, 4.06.

^1H NMR (500 MHz, C_6D_6, ppm): δ 184.93 (s, 4H), 45.34 (s, 4H), 40.69 (s, 12H), 20.82 (s, 12H), -5.94 (s, 12H), -70.98 (s, 4H).

3-U$_2$Ni$_2$. To a solution of complex **1** (120 mg, 0.17 mmol) dissolved in 2 mL THF, a 2 mL THF solution containing $Ni(COD)_2$ (46.0 mg, 0.17 mmol) was added. After shaking several times to get a clear black solution, the resulting mixture was allowed to stand undisturbed at room temperature for 24 h. Storing the solution at −30 °C for 24 h yielded product **3-U$_2$Ni$_2$** (71 mg, 60%) as black crystals. Anal. Calcd (%) for $C_{44}H_{96}Cl_4N_4Ni_2O_5P_4U_2$: C, 32.61; H, 5.97; N, 3.46. Found: C, 28.16; H, 5.20; N, 4.11. The carbon content for this species is consistently lower than the calculated values, which probably due to the loss of solvent molecules or due to the inadequate combustion of these air and moisture sensitive species. ^1H NMR (500 MHz, C_6D_6, ppm): δ 65.52 (s, 4H), 58.82 (s, 4H), 52.20 (s, 4H), 44.87 (s, 4H), 36.94 (s, 12H), 36.16 (s, 12H), 13.46 (s, 12H), 0.59 (s, 12H), -28.78 (s, 4H), -38.45 (s, 4H).

4-U$_2$Pd$_2$. To a solution of complex **1** (120 mg, 0.17 mmol) dissolved in 2 mL THF, a 5 mL THF solution containing $Pd(PPh_3)_4$ (193 mg, 0.17 mmol) was added. After shaking several times to get a clear orange solution, the resulting mixture was allowed to stand undisturbed at room temperature for 24 h. Orange-red crystals were grown from the THF solution at –30 °C. Washing the crystals with cooled THF afforded pure **4-U$_2$Pd$_2$** (65 mg, 47%). Anal. Calcd (%) for $C_{40}H_{88}Cl_4N_4O_4P_4Pd_2U_2$: C, 29.23; H, 5.40; N, 3.41. Found: C, 27.98; H, 5.23; N, 3.39. The carbon content for this species is consistently lower than the calculated values, which probably due to the loss of solvent molecules or due to the inadequate combustion of these air and moisture sensitive species.

5-(UNi)$_n$. To a solution of complex **2** (103 mg, 0.15 mmol) dissolved in 2 mL THF, a 3 mL THF solution containing $Ni(COD)_2$ (41 mg, 0.15 mmol) was added. The resulting clear solution was allowed to stand undisturbed at room temperature for 24 h. The product of **5-(UNi)$_n$** (76 mg, 68%) was obtained as black crystals after storage the THF solution at −30 °C for 24 hours. Anal. Calcd (%) for $C_{22}H_{48}Cl_2N_2NiO_3P_2U$: C, 32.30; H, 5.91; N, 3.42. Found: C, 30.37; H, 5.45; N, 3.62. The carbon content for this species is consistently lower than the calculated values, which probably due to the loss of solvent molecules or due to the inadequate combustion of these air and moisture sensitive species.

6-UPd. To a solution of complex **2** (41 mg, 0.06 mmol) dissolved in 2 mL THF, a 5 mL THF solution containing $Pd(PPh_3)_4$ (68 mg, 0.06 mmol) was added. The resulting clear solution was allowed to stand undisturbed at room temperature for 24 h. The product of **6-UPd** (31 mg, 49%) was obtained as orange crystals after storage the THF solution at −30 °C for 24 h. Anal. Calcd (%) for $C_{40}H_{63}Cl_2N_2O_3P_3PdU$: C, 42.58; H, 5.63; N, 2.48. Found: C, 42.58; H, 5.56; N, 2.52.

7-U$_2$Pt$_3$. To a solution of complex **2** (83 mg, 0.12 mmol) dissolved in 2 mL THF, a 2 mL THF solution containing $Pt(COD)_2$ (74 mg, 0.018 mmol) was added. The resulting clear solution was allowed to stand undisturbed at room temperature for 24 h. The product of **7-U$_2$Pt$_3$** (62 mg, 52%) was obtained as black crystals after storage the THF solution at −30 °C for 24 h. Anal. Calcd (%) for $C_{44}H_{96}Cl_4N_4O_6P_4Pt_3U_2$: C, 25.11; H, 4.60; N, 2.66. Found: C, 23.74; H, 4.47; N, 2.53. The carbon content for this species is consistently lower

than the calculated values, which probably due to the loss of solvent molecules or due to the inadequate combustion of these air and moisture sensitive species.

2. NMR spectra

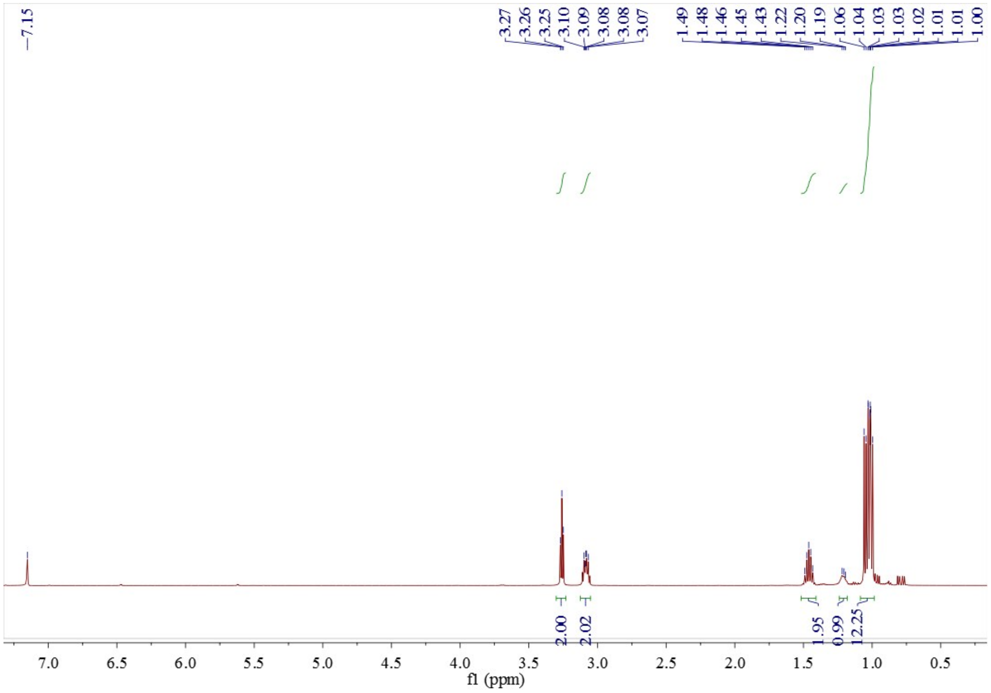

Fig. S1 ^{1}H NMR spectrum of ligand **L1** in benzene-D_6.

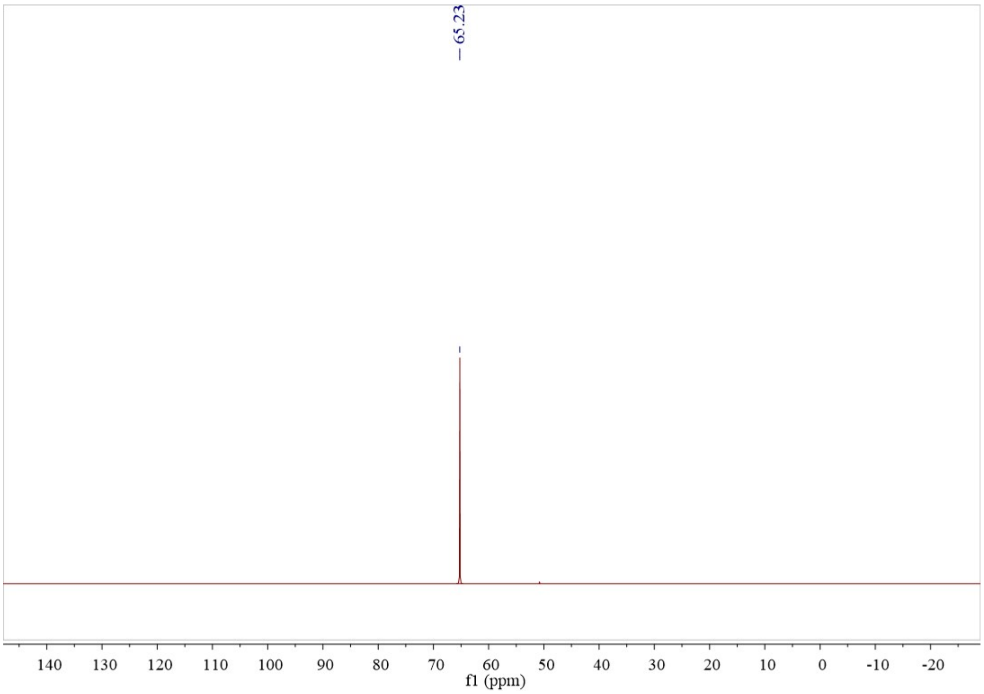

Fig. S2 ^{31}P NMR spectrum of ligand **L1** in benzene-D_6.

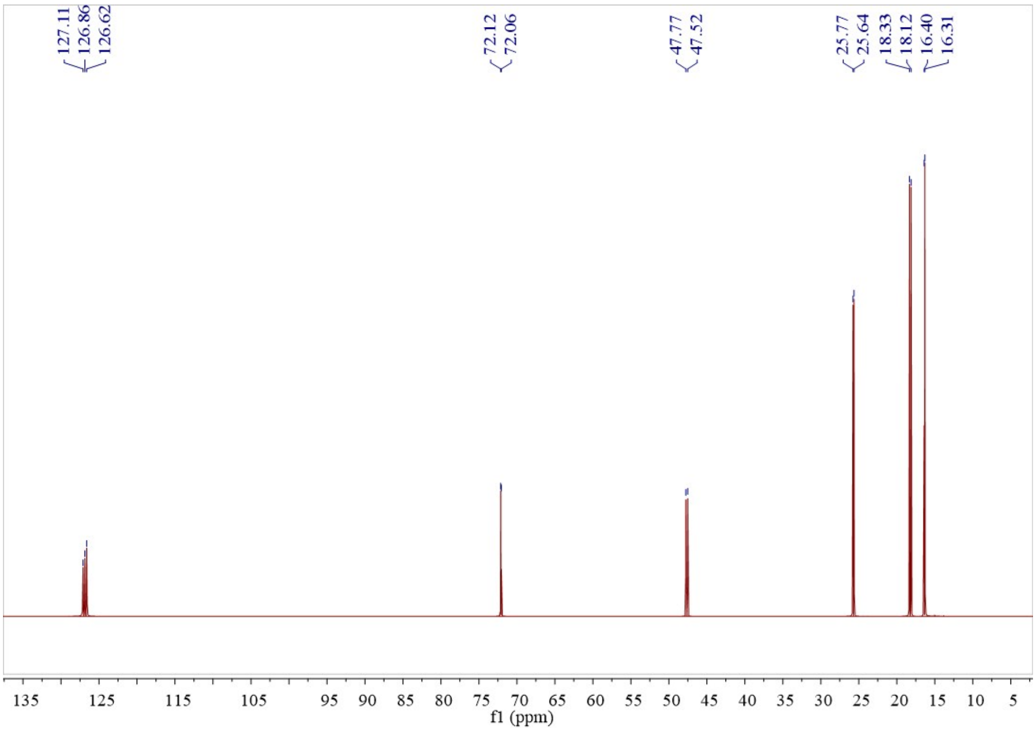

Fig. S3 ^{31}C NMR spectrum of ligand **L1** in benzene-D_6.

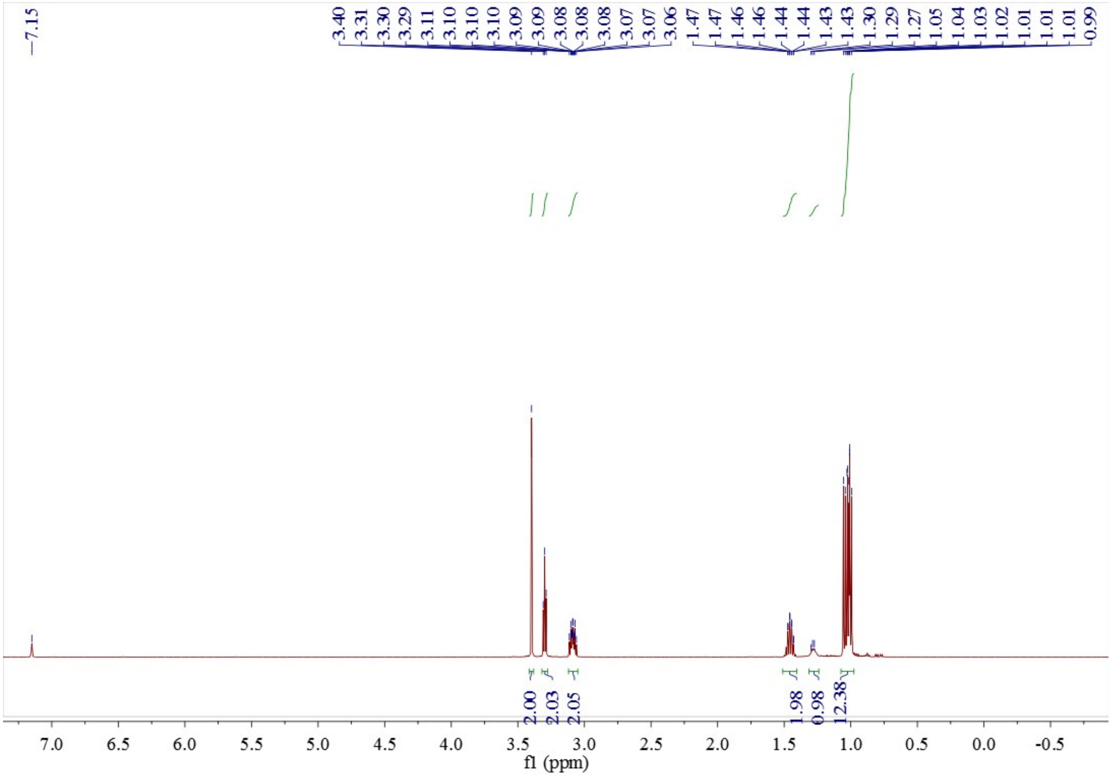

Fig. S4 ^{1}H NMR spectrum of ligand **L2** in benzene-D_6.

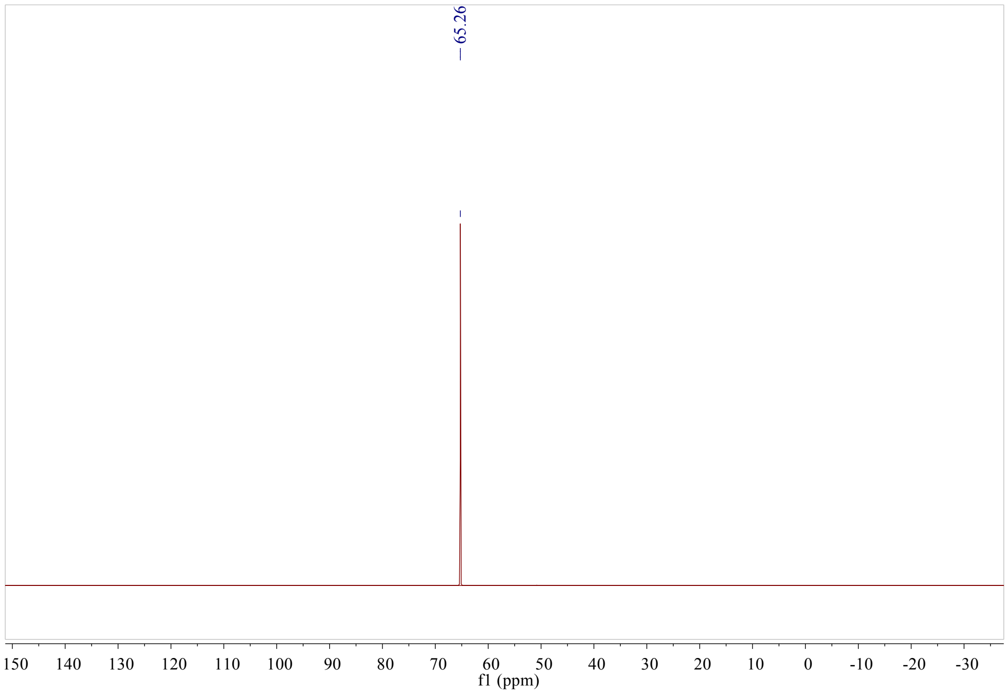

Fig. S5 ^{31}P NMR spectrum of ligand **L2** in benzene-D$_6$.

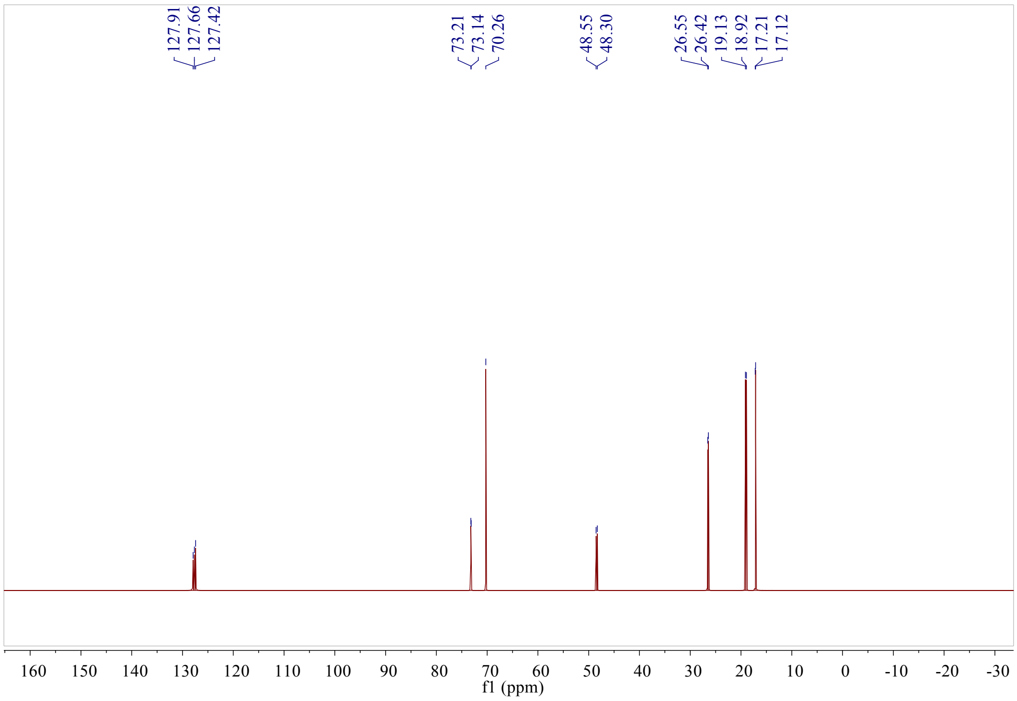

Fig. S6 ^{13}C NMR spectrum of ligand **L2** in benzene-D$_6$.

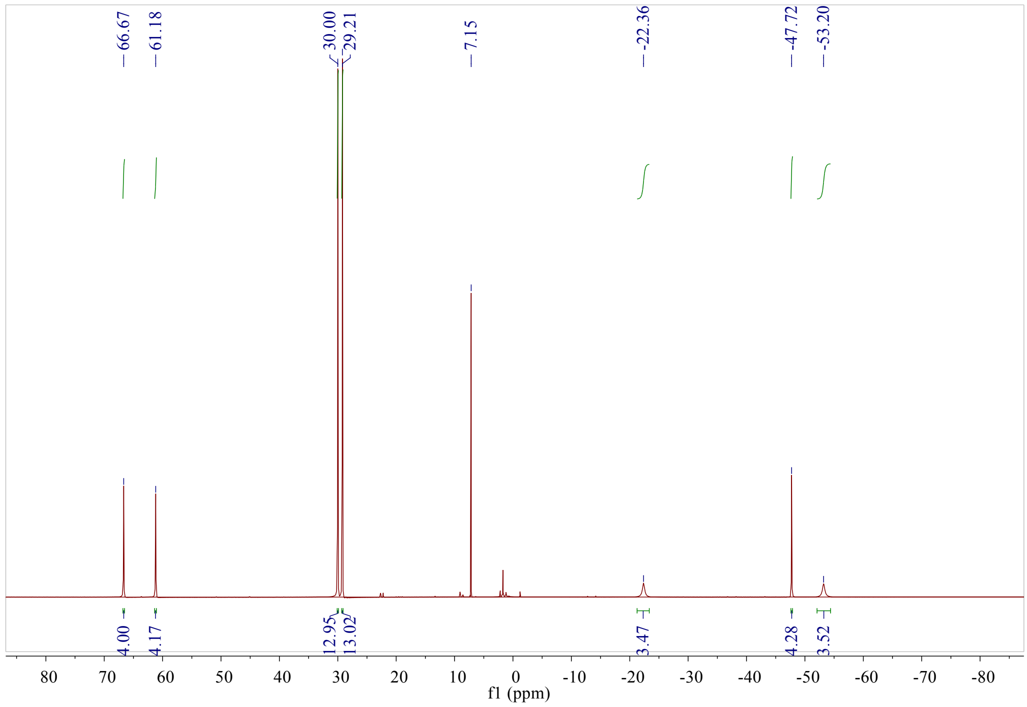

Fig. S7 ^{1}H NMR spectrum of complex **1** in benzene-D_6.

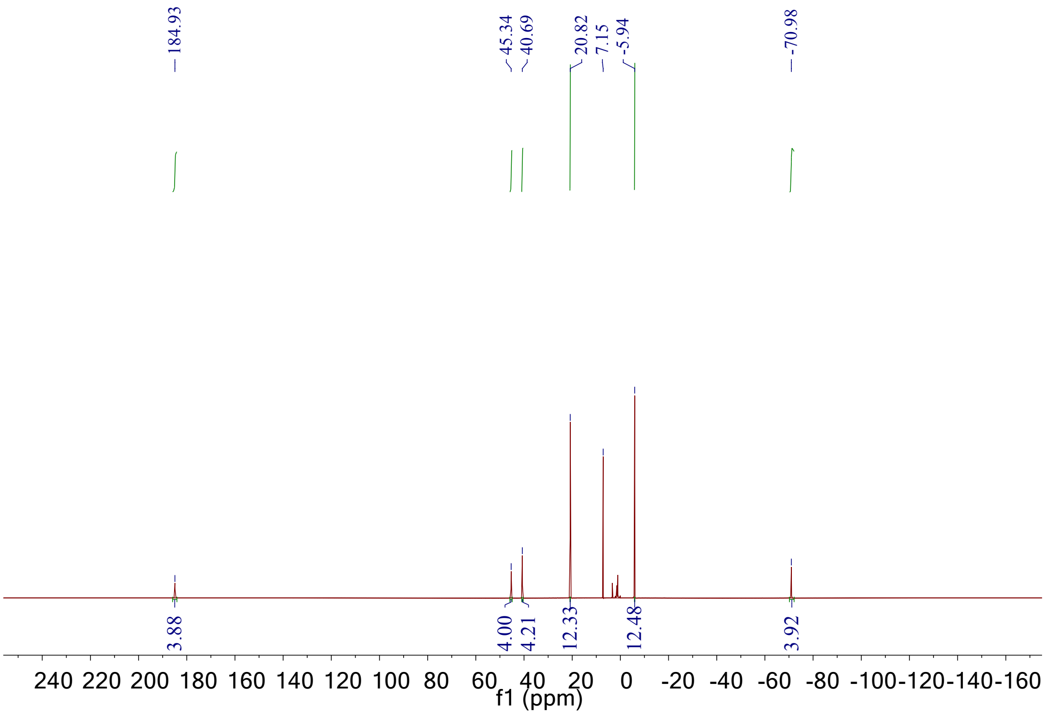

Fig. S8 ^{1}H NMR spectrum of complex **2** in benzene-D_6.

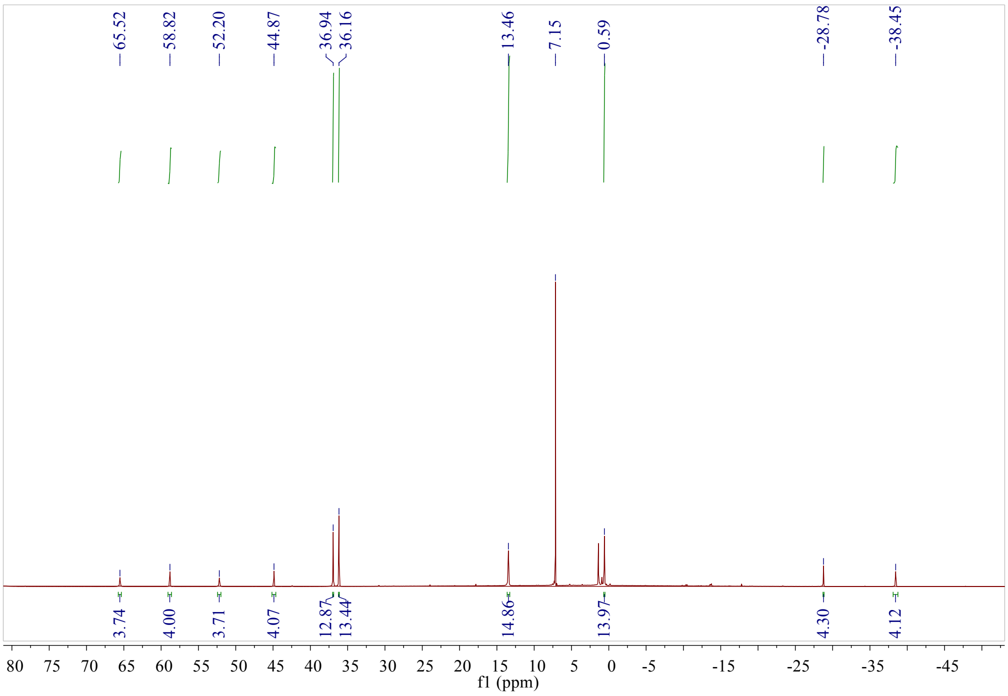

Fig. S9 ^{1}H NMR spectrum of cluster **3-U$_2$Ni$_2$** in benzene-D$_6$.

3. Powder X-ray diffraction

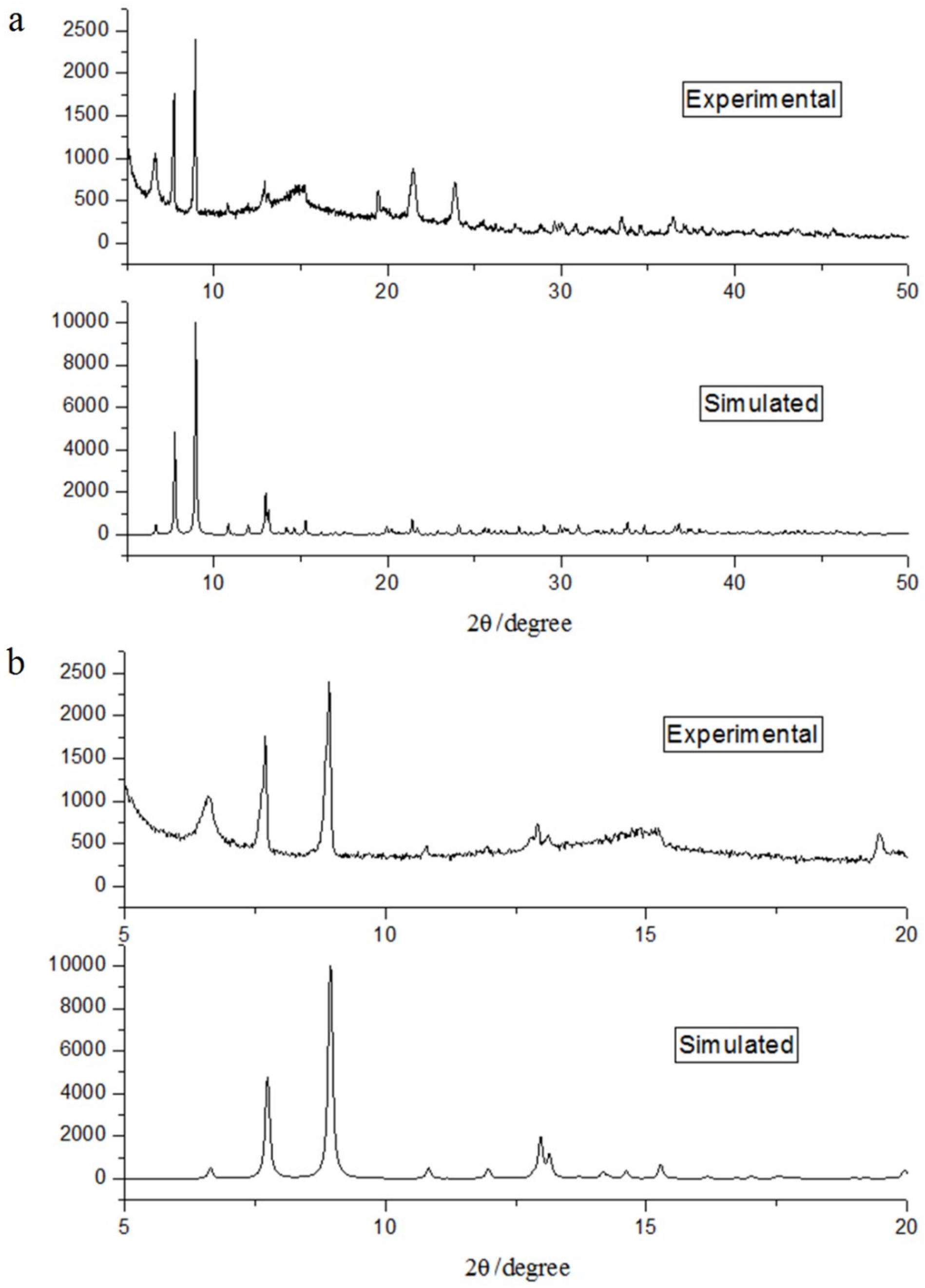

Fig. S10 Powder X-ray diffraction patterns for **4-U_2Pd_2** from 5° to 50° (a) and 5° to 20° (b).

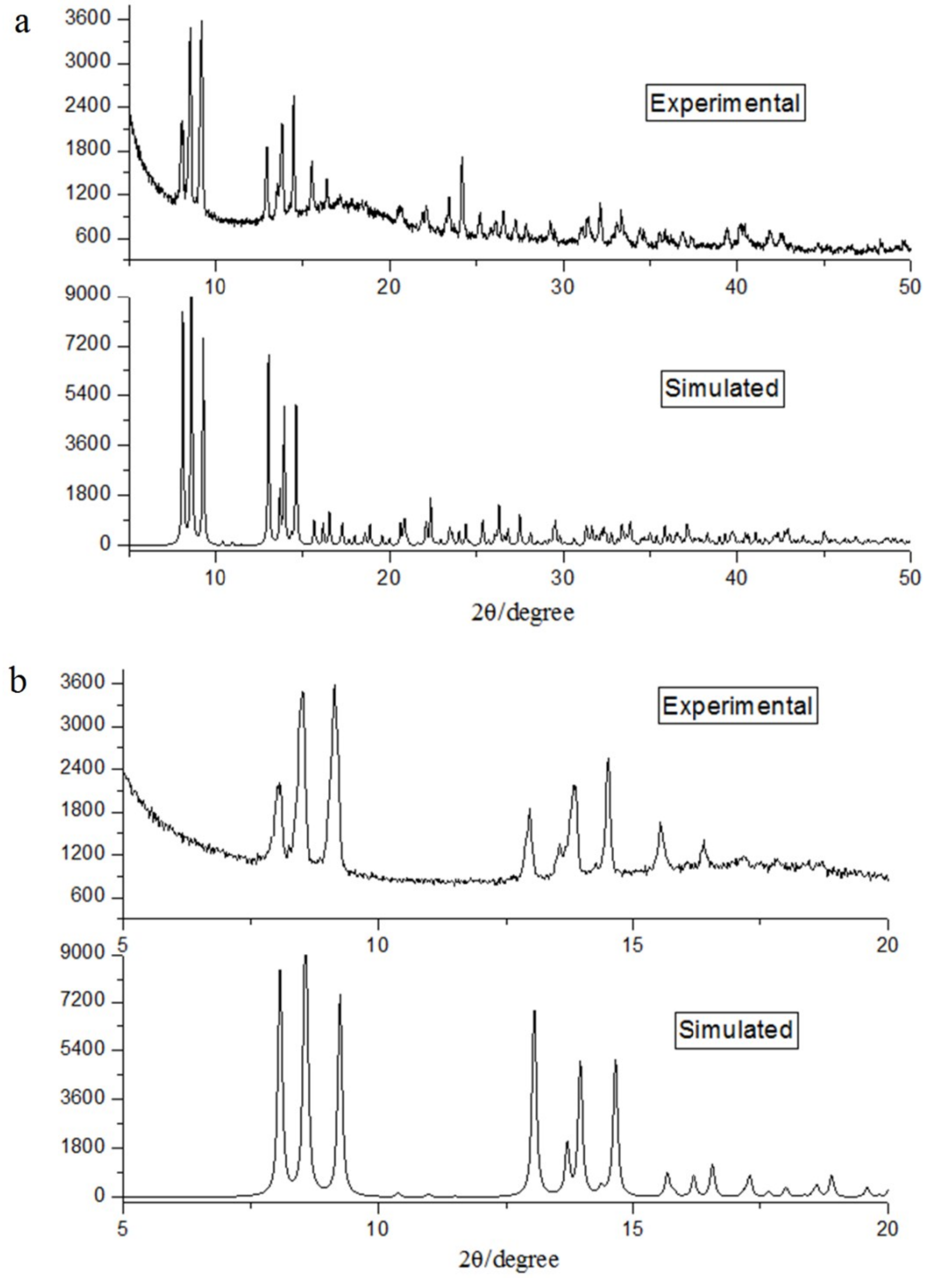

Fig. S11 Powder X-ray diffraction patterns for **5-(UNi)$_n$** from 5° to 50° (a) and 5° to 20° (b).

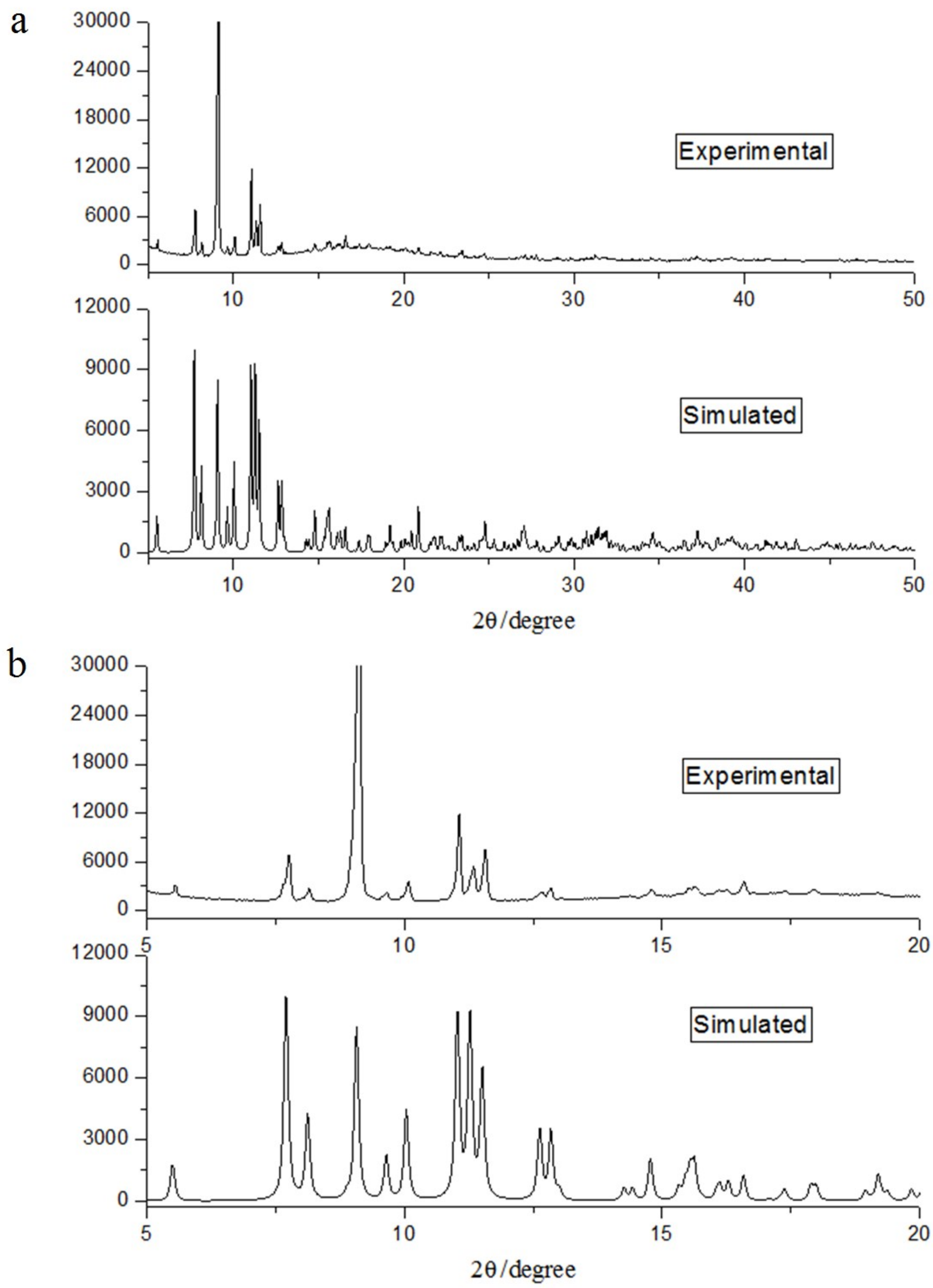

Fig. S12 Powder X-ray diffraction patterns for **6-UPd** from 5° to 50° (a) and 5° to 20° (b).

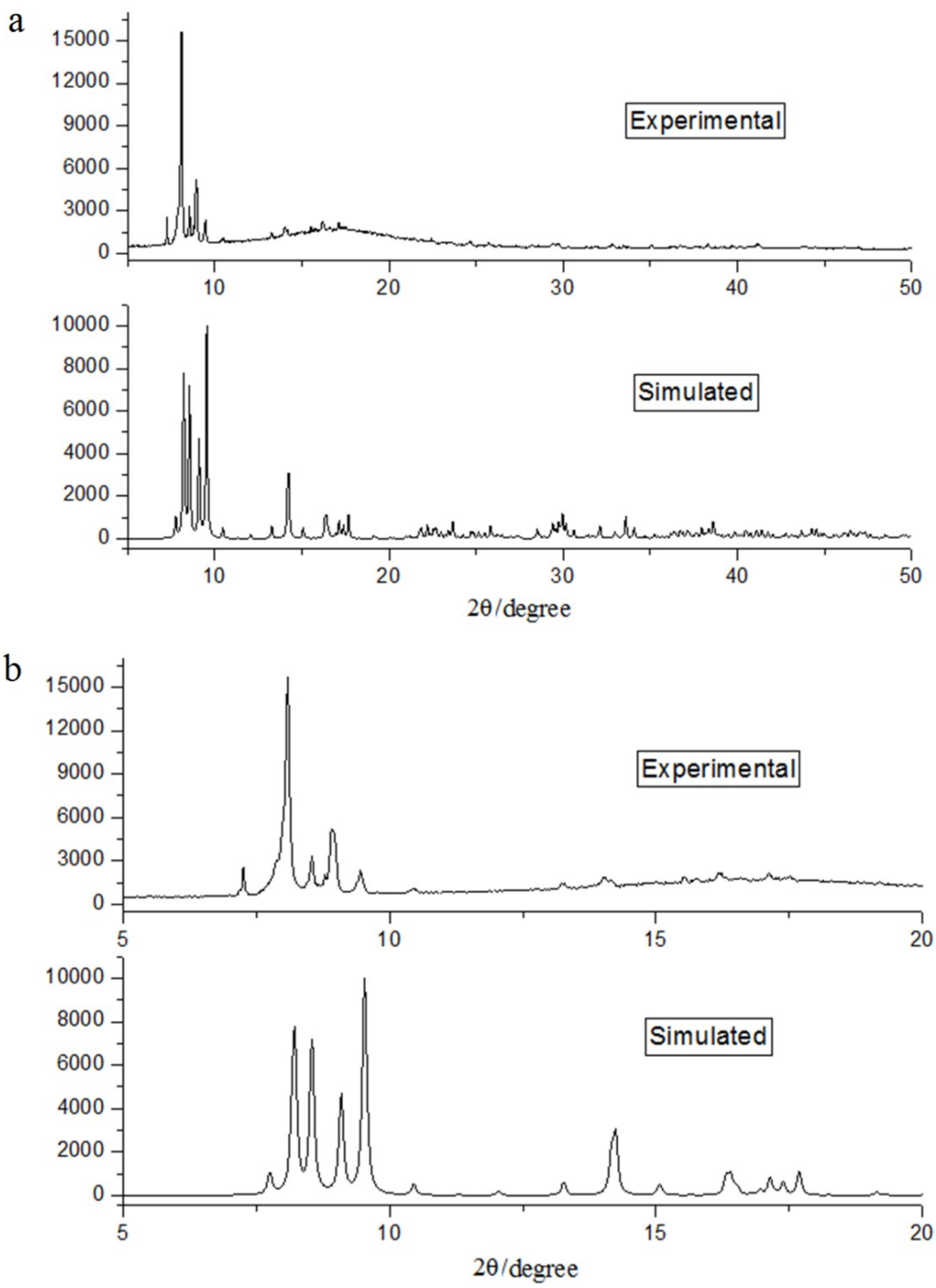

Fig. S13 Powder X-ray diffraction patterns for **7-U_2Pt_3** from 5° to 50° (a) and 5° to 20° (b).

4. X-ray Crystallographic data

Single-crystal X-ray diffraction data for complexes **1**, **2**, **3-U_2Ni_2**, **4-U_2Pd_2**, **5-$(UNi)_n$**, **6-UPd** and **7-U_2Pt_3** were collected on BRUKER D8 VENTURE PHOTON II detectors with a radiation source of Mo(Kα) (0.71073 Å) or Ga(Kα) (1.34139 Å). Integrations, cell refinement and data reduction were performed with the SAINT program.[S2] The crystal system was determined by Laue symmetry, and the space groups were assigned on the basis of systematic absences by using XPREP. All structures were solved by directed methods and refined on F^2 using full-matrix least-squares methods with SHELXTL version 6.10.[S3] All non-hydrogen atoms were refined on F^2 by full-matrix least-squares procedures with the use of anisotropic displacement parameters. Evaluation of the CIF using the CheckCIF routine at www.checkcif.iucr.org gave no A or B alert for complexes **1**, **2**, **4-U_2Pd_2**, **6-UPd**, and **7-U_2Pt_3**. An alert level B of low bond precision on C-C bonds was found when checking the CIF files of complexes **3-U_2Ni_2** and **5-$(UNi)_n$**. The quality of these two crystals was poor but they represented the best products obtained from multiple recrystallization attempts. CCDC-1979169 (**1**), 1979170 (**2**), 1979171 (**3-U_2Ni_2**), 1979172 (**4-U_2Pd_2**), 1979173 (**5-$(UNi)_n$**), 1979174 (**6-UPd**), and 1979175 (**7-U_2Pt_3**) contain the crystallographic data for this paper. These data can be obtained free of charge from the Cambridge Crystallographic Data Centre (www.ccdc.cam.ac.uk/data-request/cif). Details of the data collection and refinement for theses complexes are given in Tables S1 and S2.

Table S1. Crystal data and structure refinements for **1**, **2**, **3-U_2Ni_2** and **4-U_2Pd_2**.

Complex	**1**	**2**	**3-U_2Ni_2**	**4-U_2Pd_2**
Formula	$C_{20}H_{44}Cl_2N_2O_2P_2U$	$C_{18}H_{40}Cl_2N_2O_2P_2U$	$C_{44}H_{96}Cl_4N_4Ni_2O_5P_4U_2$	$C_{40}H_{88}Cl_4N_4O4P_4Pd_2U_2$
*M*r [g/mol]	715.44	687.39	1620.40	1643.68
Temp. [K]	173(2)	173(2)	173(2)	173(2)
Crystal system	Monoclinic	Monoclinic	Monoclinic	Orthorhombic
Space group	$P2_1/c$	$P2_1/c$	$P2_1/n$	*Pccn*
a [Å]	12.7105(10)	14.480(2)	14.5223(19)	16.3226(16)
b [Å]	28.115(2)	14.590(2)	15.8953(18)	22.813(2)
c [Å]	8.1727(7)	12.5668(18)	27.339(3)	14.7718(15)

α [°]	90	90	90	90
β [°]	105.391	102.290(4)	103.728(3)	90
γ [°]	90	90	90	90
Volume [Å^3]	2815.8(4)	2594.0(6)	6130.5(13)	5500.6(10)
Z/$D_{calcd.}$ [g/cm^3]	4/1.688	4/1.760	4/1.756	4/1.985
μ [mm^{-1}]	6.085	15.343	6.192	17.973
F(000)	1400	1336	3184	3168
ϑ range [°]	2.205 to 27.594	3.786 to 63.448	1.930 to 25.000	2.896 to 54.027
Index ranges	$-16 \le h \le 16$<br>$-25 \le k \le 36$<br>$-10 \le l \le 10$	$0 \le h \le 19$<br>$-19 \le k \le 0$<br>$-16 \le l \le 16$	$-17 \le h \le 14$<br>$-18 \le k \le 18$<br>$-27 \le l \le 32$	$-19 \le h \le 19$<br>$-27 \le k \le 27$<br>$-17 \le l \le 17$
Completeness	99.8 %	98.7 %	99.9 %	100.0 %
Data/parameters	6512 / 270	6110 / 253	10800 / 581	5045 / 279
GOF on F^2	1.091	1.125	1.002	1.013
Final R indices [I>2σ(*I*)]	R_1 = 0.0474<br>R_2 = 0.1391	R_1 = 0.0647<br>R_2 = 0.1935	R_1 = 0.0465<br>R_2 = 0.1383	R_1 = 0.0332<br>R_2 = 0.0685
R indices (all data)	R_1 = 0.0541<br>R_2 = 0.1439	R_1 = 0.0776<br>R_2 = 0.2195	R_1 = 0.0591<br>R_2 = 0.1495	R_1 = 0.0554<br>R_2 = 0.0776
Largest diff. peak and hole [e·Å^{-3}]	4.911 and -3.682	2.719 and -4.676	2.691 and -2.743	0.945 and -0.817

Table S2. Crystal data and structure refinements for complexes **5-(UNi)$_n$**, **6** and **7-U$_2$Pt$_3$**.

Complex	**5-(UNi)$_n$**	**6-UPd**	**7-U$_2$Pt$_3$**
Formula	$C_{22}H_{48}Cl_2N_2NiO_3P_2U$	$C_{40}H_{63}Cl_2N_2O_3P_3PdU$	$C_{44}H_{96}Cl_4N_4O_6P_4Pt_3U_2$
Mr [g/mol]	818.20	1128.16	2104.25

Temp. [K]	173(2)	173(2)	173(2)
Crystal system	Monoclinic	Triclinic	Monoclinic
Space group	$P2_1/n$	$P\bar{1}$	$C2/c$
a [Å]	11.3508(18)	12.208(3)	12.6630(8)
b [Å]	12.914(2)	12.209(3)	21.4809(13)
c [Å]	20.597(4)	17.048(4)	23.0838(17)
α [°]	90	108.557(7)	90
β [°]	95.165(7)	90.701(8)	99.354(2)
γ [°]	90	108.839(7)	90
Volume [$Å^3$]	3006.9(9)	2261.0(10)	6195.6(7)
Z/$D_{calcd.}$ [g/cm^3]	4/1.807	2/1.657	4/2.256
μ [mm^{-1}]	16.624	11.277	21.368
F(000)	1608	1116	3928
ϑ range/deg	3.518 to 54.617	2.398 to 54.402	3.376 to 63.565
Index ranges	$-13 \le h \le 13$<br>$-15 \le k \le 15$<br>$-22 \le l \le 24$	$-14 \le h \le 14$<br>$-14 \le k \le 14$<br>$-20 \le l \le 20$	$-16 \le h \le 16$<br>$-27 \le k \le 28$<br>$-30 \le l \le 20$
Completeness	97.1 %	99.3	99.2 %
Data/parameters	5406 / 277	8233 / 477	7657 / 311
GOF on F^2	1.112	1.042	1.066
Final R indices [I>2σ(I)]	R_1 = 0.0977<br>R_2 = 0.2146	R_1 = 0.0344<br>R_2 = 0.0940	R_1 = 0.0376<br>R_2 = 0.0948
R indices (all data)	R_1 = 0.1126<br>R_2 = 0.2235	R_1 = 0.0359<br>R_2 = 0.0956	R_1 = 0.0439<br>R_2 = 0.0985
Largest diff. peak and hole [e·$Å^{-3}$]	4.361 and -2.234	1.154 and -1.831	1.667 and -2.406

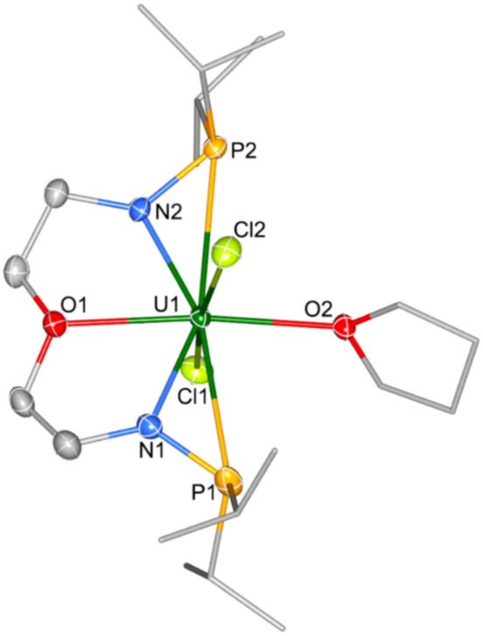

Fig. S14 Crystal structure of complex **1**. Thermal ellipsoids are drawn at 50 % probability. Hydrogen atoms are omitted for clarity.

Table S3. Selected bond lengths [Å] and angles [°] for complex **1**.

U(1)-N(2)	2.227(5)	U(1)-Cl(2)	2.6653(17)
U(1)-N(1)	2.245(5)	U(1)-P(2)	3.0466(18)
U(1)-O(1)	2.485(5)	U(1)-P(1)	3.0747(19)
U(1)-O(2)	2.485(5)	C(16)-C(14)	1.548(11)
U(1)-Cl(1)	2.6441(17)	P(2)-N(2)	1.678(6)
P(2)-C(14)	1.865(8)	O(2)-C(17)	1.455(8)
P(2)-C(11)	1.869(7)	O(2)-C(20)	1.468(8)
P(1)-N(1)	1.671(6)	O(1)-C(3)	1.446(8)
P(1)-C(8)	1.855(9)	O(1)-C(1)	1.450(8)
P(1)-C(5)	1.869(9)	N(1)-C(2)	1.460(9)
N(2)-C(4)	1.451(8)	C(5)-C(7)	1.515(13)
C(11)-C(12)	1.525(10)	C(14)-C(15)	1.530(11)
C(11)-C(13)	1.537(10)	C(8)-C(10)	1.513(15)
C(2)-C(1)	1.508(11)	C(8)-C(9)	1.530(16)

C(4)-C(3)	1.508(10)	C(17)-C(18)	1.505(11)
C(5)-C(6)	1.512(14)	C(20)-C(19)	1.502(10)
N(2)-U(1)-N(1)	129.2(2)	O(1)-U(1)-O(2)	171.33(16)
N(2)-U(1)-O(1)	64.66(18)	N(2)-U(1)-Cl(1)	89.92(16)
N(1)-U(1)-O(1)	64.98(19)	N(1)-U(1)-Cl(1)	94.73(15)
N(2)-U(1)-O(2)	119.13(18)	O(1)-U(1)-Cl(1)	87.91(13)
N(1)-U(1)-O(2)	111.70(18)	O(2)-U(1)-Cl(1)	84.36(11)
N(2)-U(1)-Cl(2)	89.63(15)	N(2)-U(1)-P(2)	32.66(14)
N(1)-U(1)-Cl(2)	93.25(15)	N(1)-U(1)-P(2)	159.94(15)
O(1)-U(1)-Cl(2)	100.86(12)	O(1)-U(1)-P(2)	97.22(11)
O(2)-U(1)-Cl(2)	87.18(11)	O(2)-U(1)-P(2)	87.21(11)
Cl(1)-U(1)-Cl(2)	170.05(6)	Cl(1)-U(1)-P(2)	93.71(5)
Cl(2)-U(1)-P(2)	80.63(5)	Cl(1)-U(1)-P(1)	92.16(6)
N(2)-U(1)-P(1)	161.21(15)	Cl(2)-U(1)-P(1)	91.46(6)
N(1)-U(1)-P(1)	32.04(15)	P(2)-U(1)-P(1)	165.03(5)
O(1)-U(1)-P(1)	96.74(11)	N(2)-P(2)-C(14)	106.1(3)
O(2)-U(1)-P(1)	79.67(11)	N(2)-P(2)-C(11)	104.7(3)
N(2)-P(2)-U(1)	45.74(19)	C(8)-P(1)-C(5)	100.6(4)
C(14)-P(2)-U(1)	118.8(2)	N(1)-P(1)-U(1)	45.47(19)
C(11)-P(2)-U(1)	130.6(2)	C(8)-P(1)-U(1)	129.3(4)
N(1)-P(1)-C(8)	111.5(4)	C(5)-P(1)-U(1)	127.6(3)
N(1)-P(1)-C(5)	107.2(3)	C(17)-O(2)-C(20)	108.7(5)
C(17)-O(2)-U(1)	122.2(4)	C(2)-N(1)-P(1)	129.9(5)
C(20)-O(2)-U(1)	123.1(4)	C(2)-N(1)-U(1)	127.3(4)
C(3)-O(1)-C(1)	113.7(5)	P(1)-N(1)-U(1)	102.5(3)
C(3)-O(1)-U(1)	115.3(4)	C(4)-N(2)-P(2)	129.6(5)

C(1)-O(1)-U(1)	115.7(4)	C(4)-N(2)-U(1)	128.3(4)
P(2)-N(2)-U(1)	101.6(3)		

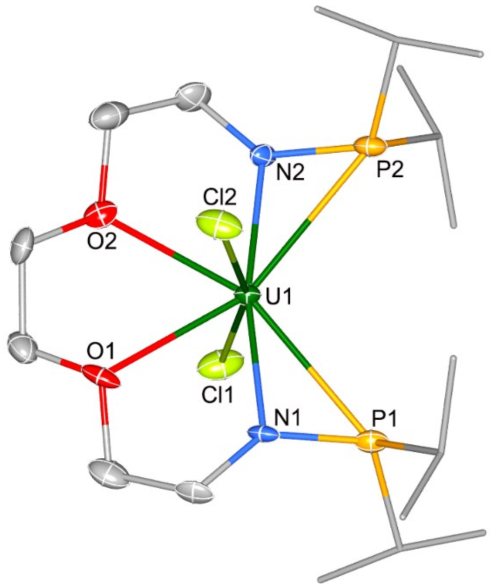

Fig. S15 Crystal structure of complex **2**. Thermal ellipsoids are drawn at 50 % probability. Hydrogen atoms are omitted for clarity.

Table S4. Selected bond lengths [Å] and angles [°] for complex **2**.

U(1)-N(2)	2.237(8)	U(1)-O(2)	2.559(7)
U(1)-N(1)	2.242(7)	U(1)-Cl(2)	2.683(2)
U(1)-O(3)	2.547(6)	U(1)-Cl(1)	2.686(3)
U(1)-P(1)	2.885(3)	N(1)-P(2)	1.636(9)
U(1)-P(2)	2.894(2)	C(1)-O(3)	1.443(12)
N(1)-C(7)	1.500(13)	C(1)-C(6)	1.498(16)
P(1)-N(2)	1.651(8)	P(1)-C(2)	1.844(12)
P(1)-C(14)	1.883(11)		
N(2)-U(1)-N(1)	167.4(3)	N(2)-U(1)-O(2)	127.9(3)
N(2)-U(1)-O(3)	64.4(3)	N(1)-U(1)-O(2)	64.7(3)

N(1)-U(1)-O(3)	128.2(3)	O(3)-U(1)-O(2)	63.6(2)
N(2)-U(1)-Cl(2)	89.4(2)	N(1)-U(1)-Cl(1)	89.6(2)
N(1)-U(1)-Cl(2)	92.5(2)	O(3)-U(1)-Cl(1)	84.46(17)
O(3)-U(1)-Cl(2)	80.35(17)	O(2)-U(1)-Cl(1)	80.69(17)
O(2)-U(1)-Cl(2)	84.22(17)	Cl(2)-U(1)-Cl(1)	162.18(8)
N(2)-U(1)-Cl(1)	92.4(2)	N(2)-U(1)-P(1)	34.8(2)
N(1)-U(1)-P(1)	132.9(2)	N(2)-U(1)-P(2)	133.4(2)
O(3)-U(1)-P(1)	98.43(16)	N(1)-U(1)-P(2)	34.3(2)
O(2)-U(1)-P(1)	159.72(16)	O(3)-U(1)-P(2)	159.43(16)
Cl(2)-U(1)-P(1)	102.65(8)	O(2)-U(1)-P(2)	98.19(16)
Cl(1)-U(1)-P(1)	88.70(8)	Cl(2)-U(1)-P(2)	88.80(8)
Cl(1)-U(1)-P(2)	102.64(8)	C(7)-N(1)-U(1)	128.0(7)
P(1)-U(1)-P(2)	100.99(6)	P(2)-N(1)-U(1)	95.2(3)
C(7)-N(1)-P(2)	136.2(7)	O(3)-C(1)-C(6)	105.1(9)

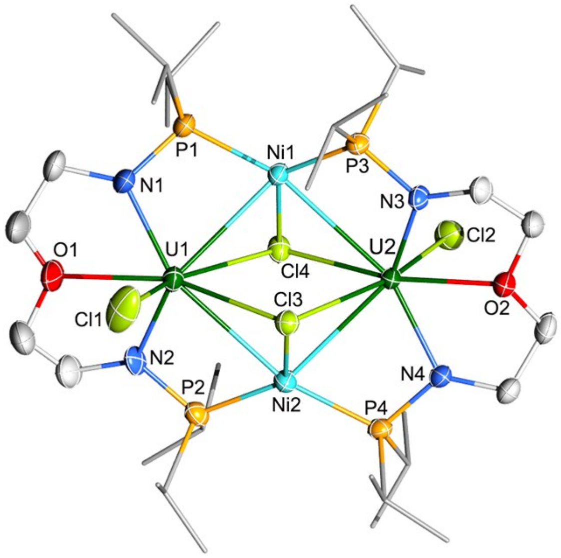

Fig. S16 Crystal structure of complex **3-U$_2$Ni$_2$**. Thermal ellipsoids are drawn at 50 % probability. Hydrogen atoms are omitted for clarity.

Table S5. Selected bond lengths [Å] and angles [°] for complex **3-U$_2$Ni$_2$**.

U(1)-N(1)	2.243(7)	U(1)-Cl(4)	2.8696(19)

U(1)-N(2)	2.248(7)	U(1)-Ni(1)	3.0477(11)
U(1)-O(1)	2.532(7)	U(1)-P(1)	3.160(2)
U(1)-Cl(1)	2.633(3)	U(1)-Ni(2)	3.1615(11)
U(1)-Cl(3)	2.867(2)	U(1)-P(2)	3.178(2)
U(2)-N(4)	2.252(7)	U(2)-Cl(4)	2.835(2)
U(2)-N(3)	2.256(7)	U(2)-Ni(2)	3.0358(10)
U(2)-O(2)	2.502(6)	U(2)-Ni(1)	3.1421(10)
U(2)-Cl(2)	2.653(2)	U(2)-P(4)	3.146(2)
U(2)-Cl(3)	2.8145(19)	U(2)-P(3)	3.159(2)
Ni(1)-P(3)	2.162(3)	N(1)-C(1)	1.462(12)
Ni(1)-P(1)	2.173(2)	N(1)-P(1)	1.679(8)
Ni(1)-Cl(4)	2.257(2)	N(2)-C(4)	1.427(12)
Ni(2)-P(2)	2.157(3)	N(2)-P(2)	1.689(8)
Ni(2)-P(4)	2.175(2)	N(3)-C(5)	1.464(11)
Ni(2)-Cl(3)	2.286(2)	N(3)-P(3)	1.672(7)
O(1)-C(2)	1.272(14)	O(1)-C(3)	1.442(13)
O(2)-C(6)	1.415(11)	P(1)-C(12)	1.851(12)
O(2)-C(7)	1.460(10)	P(1)-C(9)	1.877(9)
O(4)-C(37)	1.39(4)	P(2)-C(18)	1.864(9)
O(4)-C(40)	1.47(5)	P(2)-C(15)	1.866(10)
O(5)-C(44)	1.31(3)	P(3)-C(21)	1.863(8)
O(5)-C(41)	1.42(4)	P(3)-C(24)	1.876(8)
P(4)-C(30)	1.848(9)	P(4)-C(27)	1.864(9)
N(1)-U(1)-N(2)	119.8(3)	N(1)-U(1)-Cl(3)	130.7(2)
N(1)-U(1)-O(1)	62.3(3)	N(2)-U(1)-Cl(3)	109.4(2)
N(2)-U(1)-O(1)	64.1(3)	O(1)-U(1)-Cl(3)	154.9(2)

N(1)-U(1)-Cl(1)	93.2(2)	Cl(1)-U(1)-Cl(3)	78.85(8)
N(2)-U(1)-Cl(1)	101.6(2)	N(1)-U(1)-Cl(4)	97.6(2)
O(1)-U(1)-Cl(1)	79.0(3)	N(2)-U(1)-Cl(4)	94.1(2)
O(1)-U(1)-Cl(4)	127.6(3)	Cl(1)-U(1)-Ni(1)	118.15(9)
Cl(1)-U(1)-Cl(4)	153.29(9)	Cl(3)-U(1)-Ni(1)	70.20(4)
Cl(3)-U(1)-Cl(4)	75.59(6)	Cl(4)-U(1)-Ni(1)	44.71(4)
N(1)-U(1)-Ni(1)	71.54(19)	N(1)-U(1)-P(1)	30.62(19)
N(2)-U(1)-Ni(1)	138.6(2)	N(2)-U(1)-P(1)	138.2(2)
O(1)-U(1)-Ni(1)	131.76(19)	O(1)-U(1)-P(1)	92.07(18)
Cl(1)-U(1)-P(1)	107.04(9)	Cl(4)-U(1)-P(1)	73.07(6)
Cl(3)-U(1)-P(1)	105.64(6)	Ni(1)-U(1)-P(1)	40.93(4)
N(1)-U(1)-Ni(2)	156.09(19)	Ni(1)-U(1)-Ni(2)	86.86(3)
N(2)-U(1)-Ni(2)	70.34(19)	P(1)-U(1)-Ni(2)	126.99(4)
O(1)-U(1)-Ni(2)	134.16(17)	N(1)-U(1)-P(2)	146.9(3)
Cl(1)-U(1)-Ni(2)	106.31(7)	N(2)-U(1)-P(2)	30.6(2)
Cl(3)-U(1)-Ni(2)	44.24(4)	O(1)-U(1)-P(2)	94.47(18)
Cl(4)-U(1)-Ni(2)	58.95(5)	Cl(1)-U(1)-P(2)	105.76(9)
Cl(3)-U(1)-P(2)	80.35(6)	N(4)-U(2)-O(2)	63.9(2)
Cl(4)-U(1)-P(2)	77.55(6)	N(3)-U(2)-O(2)	64.6(2)
Ni(1)-U(1)-P(2)	119.32(4)	N(4)-U(2)-Cl(2)	92.81(18)
P(1)-U(1)-P(2)	147.21(6)	N(3)-U(2)-Cl(2)	99.20(19)
Ni(2)-U(1)-P(2)	39.78(5)	O(2)-U(2)-Cl(2)	84.03(17)
N(4)-U(2)-N(3)	125.2(3)	N(4)-U(2)-Cl(3)	94.80(17)
N(3)-U(2)-Cl(3)	94.19(18)	N(3)-U(2)-Cl(4)	113.32(19)
O(2)-U(2)-Cl(3)	118.91(16)	O(2)-U(2)-Cl(4)	163.73(16)
Cl(2)-U(2)-Cl(3)	156.84(7)	Cl(2)-U(2)-Cl(4)	80.39(7)
N(4)-U(2)-Cl(4)	121.4(2)	Cl(3)-U(2)-Cl(4)	76.97(6)

N(4)-U(2)-Ni(2)	72.14(18)	Cl(3)-U(2)-Ni(2)	45.82(4)
N(3)-U(2)-Ni(2)	139.75(18)	Cl(4)-U(2)-Ni(2)	60.86(5)
O(2)-U(2)-Ni(2)	132.03(14)	N(4)-U(2)-Ni(1)	159.54(17)
Cl(2)-U(2)-Ni(2)	117.22(6)	N(3)-U(2)-Ni(1)	70.67(18)
O(2)-U(2)-Ni(1)	134.80(14)	Ni(2)-U(2)-Ni(1)	87.41(3)
Cl(2)-U(2)-Ni(1)	97.26(6)	N(4)-U(2)-P(4)	31.53(18)
Cl(3)-U(2)-Ni(1)	69.47(4)	N(3)-U(2)-P(4)	148.7(2)
Cl(4)-U(2)-Ni(1)	43.99(4)	O(2)-U(2)-P(4)	94.94(14)
Cl(2)-U(2)-P(4)	102.03(6)	Ni(1)-U(2)-P(4)	128.25(4)
Cl(3)-U(2)-P(4)	74.50(5)	N(4)-U(2)-P(3)	154.3(2)
Cl(4)-U(2)-P(4)	92.87(6)	N(3)-U(2)-P(3)	30.56(18)
Ni(2)-U(2)-P(4)	41.16(4)	O(2)-U(2)-P(3)	94.89(14)
Cl(2)-U(2)-P(3)	99.47(6)	P(4)-U(2)-P(3)	157.15(5)
Cl(3)-U(2)-P(3)	82.69(5)	P(3)-Ni(1)-P(1)	122.27(9)
Cl(4)-U(2)-P(3)	83.14(6)	P(3)-Ni(1)-Cl(4)	128.78(9)
Ni(2)-U(2)-P(3)	120.27(4)	P(1)-Ni(1)-Cl(4)	108.58(9)
Ni(1)-U(2)-P(3)	40.13(5)	P(3)-Ni(1)-U(1)	135.69(7)
P(1)-Ni(1)-U(1)	72.32(6)	Cl(4)-Ni(1)-U(1)	63.46(5)
P(3)-Ni(1)-U(2)	70.36(6)	P(2)-Ni(2)-Cl(3)	123.10(9)
P(1)-Ni(1)-U(2)	164.48(7)	P(4)-Ni(2)-Cl(3)	108.31(9)
Cl(4)-Ni(1)-U(2)	60.75(6)	P(2)-Ni(2)-U(2)	149.93(8)
U(1)-Ni(1)-U(2)	92.33(3)	P(4)-Ni(2)-U(2)	72.13(6)
P(2)-Ni(2)-P(4)	124.24(9)	Cl(3)-Ni(2)-U(2)	61.98(5)
P(2)-Ni(2)-U(1)	70.52(7)	C(1)-N(1)-U(1)	128.7(6)
P(4)-Ni(2)-U(1)	164.22(7)	P(1)-N(1)-U(1)	106.5(4)
Cl(3)-Ni(2)-U(1)	61.03(6)	C(4)-N(2)-P(2)	124.8(7)
U(2)-Ni(2)-U(1)	92.17(3)	C(4)-N(2)-U(1)	127.8(6)

C(1)-N(1)-P(1)	124.4(6)	P(2)-N(2)-U(1)	106.8(4)
C(5)-N(3)-P(3)	127.1(6)	Ni(2)-Cl(3)-U(2)	72.21(6)
C(5)-N(3)-U(2)	126.4(5)	Ni(2)-Cl(3)-U(1)	74.73(7)
P(3)-N(3)-U(2)	106.1(4)	U(2)-Cl(3)-U(1)	103.61(6)
C(8)-N(4)-P(4)	127.8(6)	Ni(1)-Cl(4)-U(2)	75.25(6)
C(8)-N(4)-U(2)	127.5(5)	Ni(1)-Cl(4)-U(1)	71.83(6)
P(4)-N(4)-U(2)	104.6(3)	U(2)-Cl(4)-U(1)	103.03(7)
C(2)-O(1)-C(3)	120.8(9)	C(12)-P(1)-Ni(1)	113.6(3)
C(2)-O(1)-U(1)	121.5(8)	C(9)-P(1)-Ni(1)	118.0(3)
C(3)-O(1)-U(1)	115.0(6)	N(1)-P(1)-U(1)	42.9(3)
C(6)-O(2)-C(7)	115.9(7)	C(12)-P(1)-U(1)	121.7(4)
N(1)-P(1)-C(12)	104.1(5)	C(12)-P(1)-C(9)	104.0(5)
N(1)-P(1)-C(9)	106.4(4)	N(1)-P(1)-Ni(1)	109.6(3)

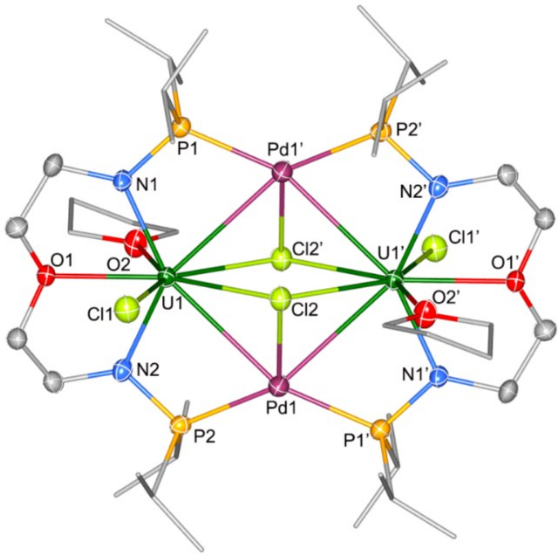

Fig. S17 Crystal structure of complex **4-U$_2$Pd$_2$**. Thermal ellipsoids are drawn at 50 % probability. Hydrogen atoms are omitted for clarity.

Table S6. Selected bond lengths [Å] and angles [°] for complex **4-U$_2$Pd$_2$**.

U(1)-N(1)	2.246(5)	U(1)-Pd(1)	3.2655(6)
U(1)-N(2)	2.271(5)	U(1)-Pd(1)#1	3.2992(6)

U(1)-O(1)	2.520(4)	Pd(1)-P(2)	2.2825(17)
U(1)-Cl(1)	2.6928(16)	Pd(1)-P(1)#1	2.3101(17)
U(1)-O(2)	2.757(4)	Pd(1)-Cl(2)	2.6893(16)
U(1)-Cl(2)#1	2.8339(16)	Pd(1)-U(1)#1	3.2992(6)
U(1)-Cl(2)	2.9229(15)	Cl(2)-U(1)#1	2.8340(16)
U(1)-P(1)	3.2069(17)	N(1)-C(1)	1.479(8)
U(1)-P(2)	3.2251(16)	N(1)-P(1)	1.673(5)
N(2)-P(2)	1.671(5)	N(2)-C(4)	1.464(8)
N(2)-P(2)	1.671(5)	P(1)-Pd(1)#1	2.3100(17)
N(2)-P(2)	1.671(5)	P(2)-C(8)	1.854(7)
N(2)-P(2)	1.671(5)	P(2)-C(5)	1.862(6)
N(2)-P(2)	1.671(5)	C(1)-C(2)	1.512(9)
N(2)-P(2)	1.671(5)	N(2)-P(2)	1.671(5)
N(1)-U(1)-N(2)	130.73(18)	O(1)-U(1)-Cl(1)	75.10(10)
N(1)-U(1)-O(1)	65.63(15)	N(1)-U(1)-O(2)	80.38(16)
N(2)-U(1)-O(1)	65.61(16)	N(2)-U(1)-O(2)	79.57(16)
N(1)-U(1)-Cl(1)	86.40(14)	O(1)-U(1)-O(2)	73.35(13)
N(2)-U(1)-Cl(1)	87.87(13)	Cl(1)-U(1)-O(2)	148.44(10)
N(1)-U(1)-Cl(2)#1	108.13(13)	N(1)-U(1)-Cl(2)	111.90(13)
N(2)-U(1)-Cl(2)#1	106.67(14)	N(2)-U(1)-Cl(2)	112.50(12)
O(1)-U(1)-Cl(2)#1	143.86(10)	O(1)-U(1)-Cl(2)	146.98(10)
Cl(1)-U(1)-Cl(2)#1	141.04(5)	Cl(1)-U(1)-Cl(2)	71.88(5)
O(2)-U(1)-Cl(2)#1	70.51(10)	O(2)-U(1)-Cl(2)	139.67(10)
Cl(2)#1-U(1)-Cl(2)	69.16(5)	Cl(2)#1-U(1)-P(1)	84.26(4)
N(1)-U(1)-P(1)	29.56(13)	Cl(2)-U(1)-P(1)	87.51(4)
N(2)-U(1)-P(1)	159.44(13)	N(1)-U(1)-P(2)	160.03(13)

O(1)-U(1)-P(1)	95.19(10)	N(2)-U(1)-P(2)	29.36(13)
Cl(1)-U(1)-P(1)	94.34(5)	O(1)-U(1)-P(2)	94.82(10)
O(2)-U(1)-P(1)	88.14(10)	Cl(1)-U(1)-P(2)	92.57(5)
O(2)-U(1)-P(2)	90.42(10)	Cl(2)#1-U(1)-P(2)	85.01(4)
Cl(2)-U(1)-P(2)	86.56(4)	O(2)-U(1)-Pd(1)	104.67(9)
P(1)-U(1)-P(2)	169.03(4)	Cl(2)#1-U(1)-Pd(1)	56.29(3)
N(1)-U(1)-Pd(1)	158.61(12)	Cl(2)-U(1)-Pd(1)	51.15(3)
N(2)-U(1)-Pd(1)	70.53(13)	P(1)-U(1)-Pd(1)	129.07(3)
O(1)-U(1)-Pd(1)	135.75(9)	P(2)-U(1)-Pd(1)	41.17(3)
Cl(1)-U(1)-Pd(1)	98.07(4)	N(1)-U(1)-Pd(1)#1	71.11(12)
N(2)-U(1)-Pd(1)#1	156.24(13)	P(2)-Pd(1)-P(1)#1	131.21(6)
O(1)-U(1)-Pd(1)#1	136.69(9)	P(2)-Pd(1)-Cl(2)	115.94(6)
Cl(1)-U(1)-Pd(1)#1	104.69(4)	P(1)#1-Pd(1)-Cl(2)	108.43(6)
O(2)-U(1)-Pd(1)#1	97.94(9)	P(2)-Pd(1)-U(1)	68.46(4)
Cl(2)#1-U(1)-Pd(1)#1	51.33(3)	P(1)#1-Pd(1)-U(1)	159.38(5)
Cl(2)-U(1)-Pd(1)#1	55.20(3)	Cl(2)-Pd(1)-U(1)	57.83(3)
P(1)-U(1)-Pd(1)#1	41.56(3)	P(2)-Pd(1)-U(1)#1	158.94(4)
P(2)-U(1)-Pd(1)#1	128.11(3)	P(1)#1-Pd(1)-U(1)#1	67.08(4)
Pd(1)-U(1)-Pd(1)#1	87.551(14)	Cl(2)-Pd(1)-U(1)#1	55.36(3)
U(1)-Pd(1)-U(1)#1	92.449(14)	Pd(1)-Cl(2)-U(1)	71.02(4)
Pd(1)-Cl(2)-U(1)#1	73.30(4)	U(1)#1-Cl(2)-U(1)	110.84(5)

Symmetry transformations used to generate equivalent atoms:

#1 -x+1,-y+1,-z+1

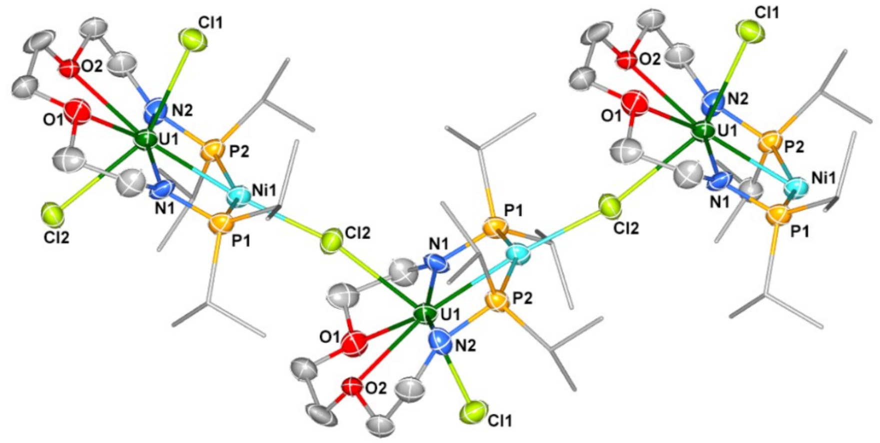

Fig. S18 Crystal structure of complex **5-(UNi)$_n$**. Thermal ellipsoids are drawn at 50 % probability. Hydrogen atoms are omitted for clarity.

Table S7. Selected bond lengths [Å] and angles [°] for complex **5-(UNi)$_n$**.

Ni(1)-P(2)	2.224(5)	U(1)-N(1)	2.241(16)
Ni(1)-P(1)	2.226(5)	U(1)-O(2)	2.523(13)
Ni(1)-Cl(2)	2.409(5)	U(1)-O(1)	2.548(15)
Ni(1)-U(1)	2.620(3)	U(1)-Cl(1)	2.711(5)
U(1)-N(2)	2.236(17)	U(1)-Cl(2)#1	2.820(5)
U(1)-P(1)	2.969(5)	N(2)-C(6)	1.51(3)
U(1)-P(2)	2.994(5)	N(2)-P(2)	1.656(17)
Cl(2)-U(1)#2	2.820(5)	O(1)-C(3)	1.42(3)
N(1)-C(1)	1.44(3)	O(1)-C(2)	1.45(3)
N(1)-P(1)	1.648(16)	O(2)-C(4)	1.43(3)
O(2)-C(5)	1.46(2)	P(2)-C(16)	1.83(2)
P(1)-C(7)	1.85(2)	P(2)-C(13)	1.83(2)
P(1)-C(10)	1.87(2)	C(1)-C(2)	1.55(3)
P(2)-Ni(1)-P(1)	150.6(2)	Cl(2)-Ni(1)-U(1)	170.77(18)
P(2)-Ni(1)-Cl(2)	109.2(2)	N(2)-U(1)-N(1)	158.6(6)
P(1)-Ni(1)-Cl(2)	100.2(2)	N(2)-U(1)-O(2)	67.1(5)

P(2)-Ni(1)-U(1)	75.85(15)	N(1)-U(1)-O(2)	134.0(5)
P(1)-Ni(1)-U(1)	75.06(16)	N(2)-U(1)-O(1)	134.1(5)
N(1)-U(1)-O(1)	67.3(5)	N(2)-U(1)-Ni(1)	79.1(4)
O(2)-U(1)-O(1)	67.1(5)	N(1)-U(1)-Ni(1)	79.7(4)
O(2)-U(1)-Ni(1)	146.2(3)	N(2)-U(1)-Cl(1)	91.8(5)
O(1)-U(1)-Ni(1)	146.5(3)	N(1)-U(1)-Cl(1)	95.2(5)
O(2)-U(1)-Cl(1)	80.1(3)	O(2)-U(1)-Cl(2)#1	79.2(3)
O(1)-U(1)-Cl(1)	77.7(4)	O(1)-U(1)-Cl(2)#1	79.7(4)
Ni(1)-U(1)-Cl(1)	100.70(14)	Ni(1)-U(1)-Cl(2)#1	105.42(13)
N(2)-U(1)-Cl(2)#1	94.6(5)	Cl(1)-U(1)-Cl(2)#1	153.83(17)
N(1)-U(1)-Cl(2)#1	88.0(5)	N(2)-U(1)-P(1)	125.5(4)
N(1)-U(1)-P(1)	33.3(4)	Cl(2)#1-U(1)-P(1)	97.58(15)
O(2)-U(1)-P(1)	167.4(3)	N(2)-U(1)-P(2)	33.1(4)
O(1)-U(1)-P(1)	100.4(3)	N(1)-U(1)-P(2)	125.7(4)
Ni(1)-U(1)-P(1)	46.43(11)	O(2)-U(1)-P(2)	100.2(3)
Cl(1)-U(1)-P(1)	99.25(16)	O(1)-U(1)-P(2)	167.0(3)
Ni(1)-U(1)-P(2)	46.09(11)	P(1)-U(1)-P(2)	92.44(14)
Cl(1)-U(1)-P(2)	98.41(16)	Ni(1)-Cl(2)-U(1)#2	167.2(2)
Cl(2)#1-U(1)-P(2)	100.74(15)		

Symmetry transformations used to generate equivalent atoms:

#1 -x+3/2,y-1/2,-z+1/2 #2 -x+3/2,y+1/2,-z+1/2

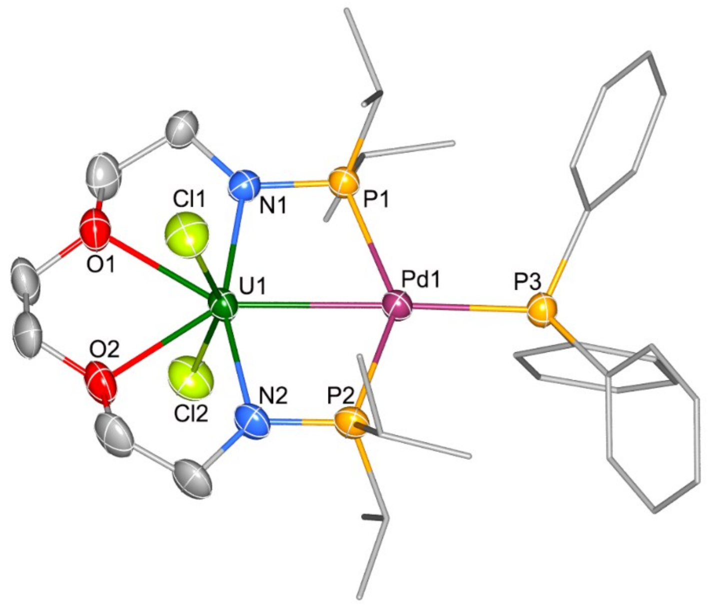

Fig. S19 Crystal structure of complex **6-UPd**. Thermal ellipsoids are drawn at 50 % probability. Hydrogen atoms are omitted for clarity.

Table S8. Selected bond lengths [Å] and angles [°] for complex **6-UPd**.

U(1)-N(2)	2.216(4)	U(1)-Cl(2)	2.7008(13)
U(1)-N(1)	2.222(4)	U(1)-Pd(1)	2.9041(6)
U(1)-O(1)	2.481(3)	U(1)-P(2)	3.0551(12)
U(1)-O(2)	2.510(3)	U(1)-P(1)	3.0617(14)
U(1)-Cl(1)	2.7002(14)	Pd(1)-P(1)	2.3770(12)
Pd(1)-P(2)	2.4053(12)	P(2)-C(16)	1.852(5)
Pd(1)-P(3)	2.4295(12)	P(2)-C(13)	1.859(5)
P(1)-N(1)	1.657(4)	P(3)-C(31)	1.825(5)
P(1)-C(10)	1.857(5)	P(3)-C(25)	1.835(5)
P(1)-C(7)	1.860(5)	P(3)-C(19)	1.851(5)
O(1)-C(3)	1.445(6)	O(3)-C(40)	1.439(11)
O(1)-C(2)	1.455(7)	N(1)-C(1)	1.469(6)
O(2)-C(5)	1.427(6)	N(2)-C(6)	1.465(6)
O(2)-C(4)	1.434(6)	C(1)-C(2)	1.500(8)
O(3)-C(37)	1.365(12)		

N(2)-U(1)-N(1)	158.14(15)	O(1)-U(1)-Cl(1)	79.55(9)
N(2)-U(1)-O(1)	133.92(13)	O(2)-U(1)-Cl(1)	82.11(9)
N(1)-U(1)-O(1)	67.94(13)	N(2)-U(1)-Cl(2)	93.78(10)
N(2)-U(1)-O(2)	67.07(12)	N(1)-U(1)-Cl(2)	90.46(11)
N(1)-U(1)-O(2)	134.72(13)	O(1)-U(1)-Cl(2)	79.78(9)
O(1)-U(1)-O(2)	66.94(12)	O(2)-U(1)-Cl(2)	78.18(9)
N(2)-U(1)-Cl(1)	91.45(10)	Cl(1)-U(1)-Cl(2)	155.73(5)
N(1)-U(1)-Cl(1)	93.44(11)	N(2)-U(1)-Pd(1)	79.60(10)
N(1)-U(1)-Pd(1)	78.61(10)	O(1)-U(1)-Pd(1)	146.23(9)
O(2)-U(1)-Pd(1)	146.67(8)	Cl(2)-U(1)-Pd(1)	105.66(4)
Cl(1)-U(1)-Pd(1)	98.60(4)	N(2)-U(1)-P(2)	32.07(10)
N(1)-U(1)-P(2)	126.11(11)	O(1)-U(1)-P(1)	99.69(9)
O(1)-U(1)-P(2)	165.70(9)	O(2)-U(1)-P(1)	166.38(8)
O(2)-U(1)-P(2)	99.15(8)	Cl(1)-U(1)-P(1)	98.50(4)
Cl(1)-U(1)-P(2)	95.75(4)	Cl(2)-U(1)-P(1)	97.38(4)
Cl(2)-U(1)-P(2)	101.23(4)	Pd(1)-U(1)-P(1)	46.87(2)
Pd(1)-U(1)-P(2)	47.53(2)	P(2)-U(1)-P(1)	94.34(3)
N(2)-U(1)-P(1)	126.37(10)	P(1)-Pd(1)-P(2)	139.43(4)
N(1)-U(1)-P(1)	31.78(11)	P(1)-Pd(1)-P(3)	110.62(4)
P(2)-Pd(1)-P(3)	109.95(4)	N(1)-P(1)-Pd(1)	107.95(14)
P(1)-Pd(1)-U(1)	70.05(4)	C(10)-P(1)-Pd(1)	118.33(19)
P(2)-Pd(1)-U(1)	69.53(3)	C(7)-P(1)-Pd(1)	111.27(18)
P(3)-Pd(1)-U(1)	175.64(3)	N(1)-P(1)-U(1)	44.94(13)
N(1)-P(1)-C(10)	105.1(2)	C(10)-P(1)-U(1)	124.48(18)
N(1)-P(1)-C(7)	108.9(2)	C(7)-P(1)-U(1)	127.2(2)
C(10)-P(1)-C(7)	104.8(3)	Pd(1)-P(1)-U(1)	63.08(3)

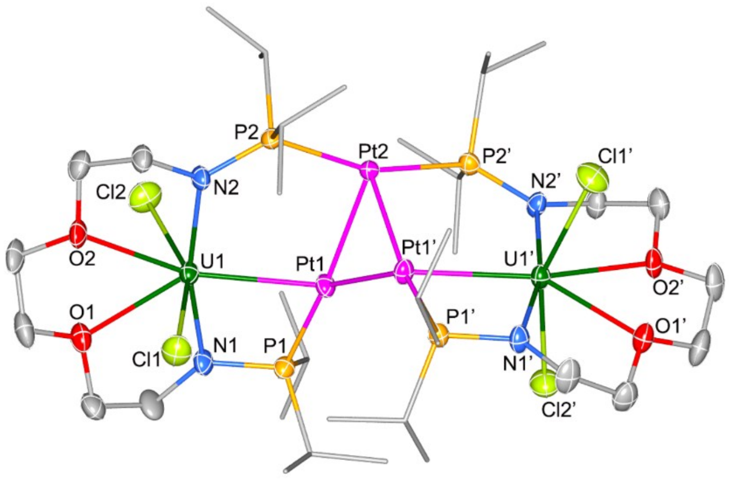

Fig. S20 Crystal structure of complex **7-U_2Pt_3**. Thermal ellipsoids are drawn at 50 % probability. Hydrogen atoms are omitted for clarity.

Table S9. Selected bond lengths [Å] and angles [°] for complex **7-U_2Pt_3**.

U(1)-N(1)	2.286(5)	U(1)-Cl(2)	2.6865(16)
U(1)-N(2)	2.343(5)	U(1)-Pt(1)	2.7584(4)
U(1)-O(2)	2.541(4)	U(1)-P(1)	3.1082(15)
U(1)-O(1)	2.600(4)	Pt(1)-P(1)	2.2161(14)
U(1)-Cl(1)	2.6725(16)	Pt(1)-Pt(2)	2.6074(4)
Pt(1)-Pt(1)#1	2.8317(5)	N(1)-P(1)	1.630(5)
Pt(2)-P(2)	2.3060(15)	N(2)-C(6)	1.485(7)
Pt(2)-P(2)#1	2.3060(15)	N(2)-P(2)	1.640(5)
Pt(2)-Pt(1)#1	2.6074(4)	O(1)-C(3)	1.424(8)
N(1)-C(1)	1.463(7)	O(1)-C(2)	1.439(8)
O(2)-C(5)	1.427(8)	O(3)-C(19)	1.555(17)
O(2)-C(4)	1.433(7)	P(1)-C(10)	1.843(6)
O(3)-C(22)	1.494(15)	P(1)-C(7)	1.850(6)
P(2)-C(16)	1.845(6)	P(2)-C(13)	1.866(6)
C(1)-C(2)	1.508(9)		
N(1)-U(1)-N(2)	166.60(18)	O(2)-U(1)-O(1)	61.51(14)

N(1)-U(1)-O(2)	125.74(16)	N(1)-U(1)-Cl(1)	93.94(14)
N(2)-U(1)-O(2)	67.53(15)	N(2)-U(1)-Cl(1)	90.78(13)
N(1)-U(1)-O(1)	64.22(15)	O(2)-U(1)-Cl(1)	78.14(11)
N(2)-U(1)-O(1)	129.02(15)	O(1)-U(1)-Cl(1)	80.94(11)
N(1)-U(1)-Cl(2)	91.03(14)	N(1)-U(1)-Pt(1)	74.52(13)
N(2)-U(1)-Cl(2)	89.85(13)	N(2)-U(1)-Pt(1)	92.42(12)
O(2)-U(1)-Cl(2)	79.44(11)	O(2)-U(1)-Pt(1)	159.34(9)
O(1)-U(1)-Cl(2)	79.65(11)	O(1)-U(1)-Pt(1)	138.47(10)
Cl(1)-U(1)-Cl(2)	155.44(6)	Cl(1)-U(1)-Pt(1)	97.97(4)
N(1)-U(1)-P(1)	30.63(13)	Cl(2)-U(1)-Pt(1)	106.53(4)
N(2)-U(1)-P(1)	136.23(12)	P(1)-Pt(1)-Pt(2)	163.89(4)
O(2)-U(1)-P(1)	156.16(10)	P(1)-Pt(1)-U(1)	76.47(4)
O(1)-U(1)-P(1)	94.75(10)	Pt(2)-Pt(1)-U(1)	102.594(9)
Cl(1)-U(1)-P(1)	96.94(5)	P(1)-Pt(1)-Pt(1)#1	138.02(4)
Cl(2)-U(1)-P(1)	99.51(5)	Pt(2)-Pt(1)-Pt(1)#1	57.111(7)
Pt(1)-U(1)-P(1)	43.89(3)	U(1)-Pt(1)-Pt(1)#1	116.123(15)
P(2)-Pt(2)-Pt(1)	95.94(4)	P(2)-Pt(2)-P(2)#1	160.59(8)
P(2)#1-Pt(2)-Pt(1)	100.35(4)	P(2)#1-Pt(2)-Pt(1)#1	95.94(4)
P(2)-Pt(2)-Pt(1)#1	100.35(4)	Pt(1)-Pt(2)-Pt(1)#1	65.777(14)
C(1)-N(1)-P(1)	128.8(4)	C(6)-N(2)-P(2)	119.0(4)
C(1)-N(1)-U(1)	127.4(4)	C(6)-N(2)-U(1)	116.9(4)
P(1)-N(1)-U(1)	103.8(2)	P(2)-N(2)-U(1)	123.6(3)

Symmetry transformations used to generate equivalent atoms:

#1 -x+1,y,-z+3/2

6. Magnetic measurements data

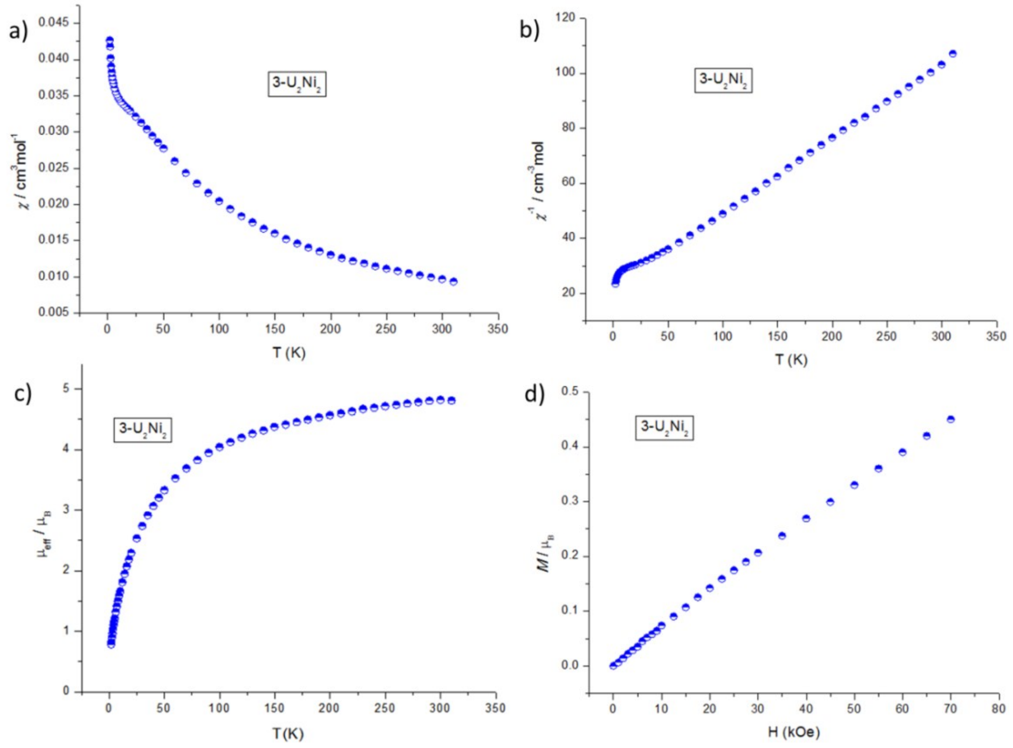

Fig. S21 Variable temperature SQUID data plotted of complex **3-U_2Ni_2** as χ *vs* T (a), 1/χ *vs* T (b), μ_{eff} *vs* T per molecule (c), and field-dependent SQUID magnetization at 1.8 K (d).

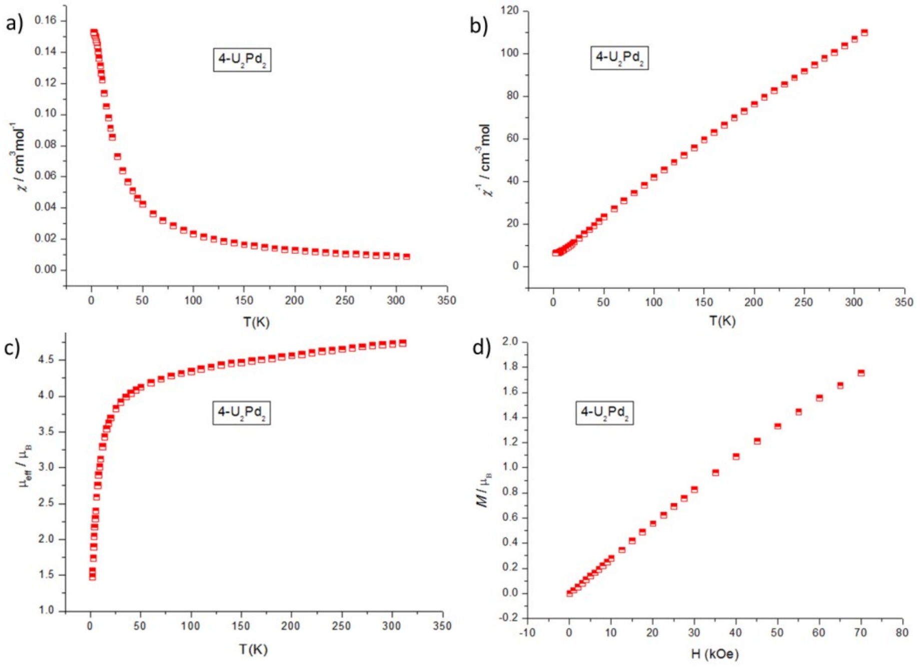

Fig. S22 Variable temperature SQUID data plotted of complex **4-U_2Pd_2** as χ *vs* T (a), 1/χ *vs* T (b), μ_{eff} *vs* T per molecule (c), and field-dependent SQUID magnetization at 1.8 K (d).

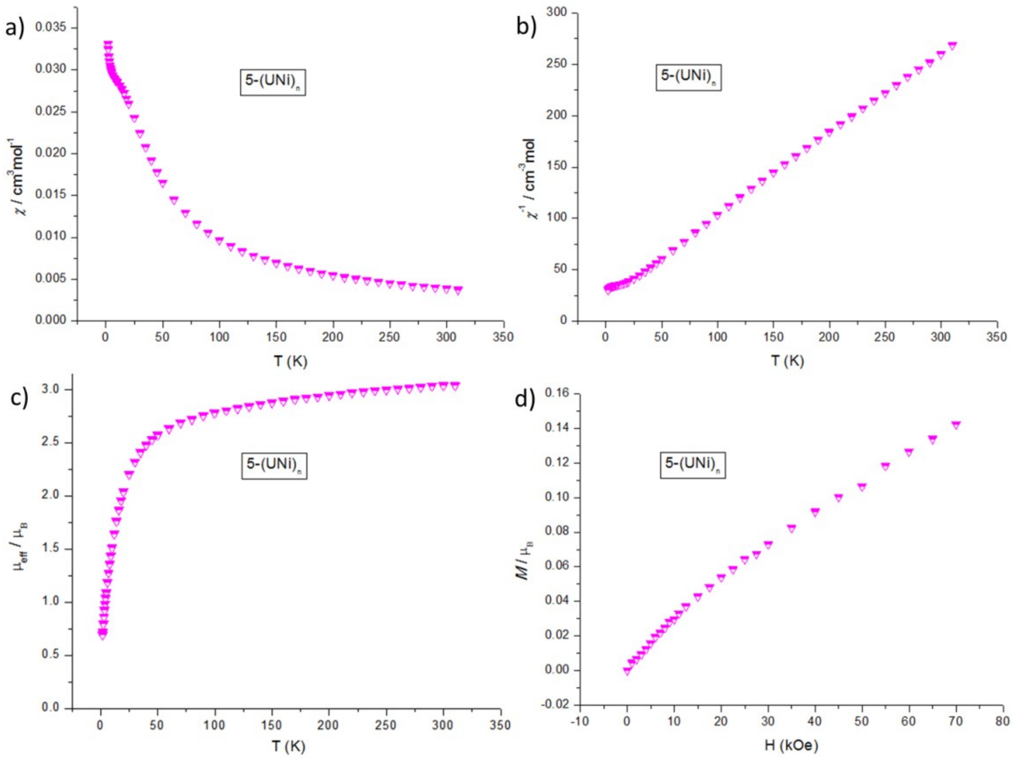

Fig. S23 Variable temperature SQUID data plotted of complex **5-(UNi)$_n$** χ *vs* T (a), 1/χ *vs* T (b), μ_{eff} *vs* T per uranium ion (c), and field-dependent SQUID magnetization at 1.8 K (d).

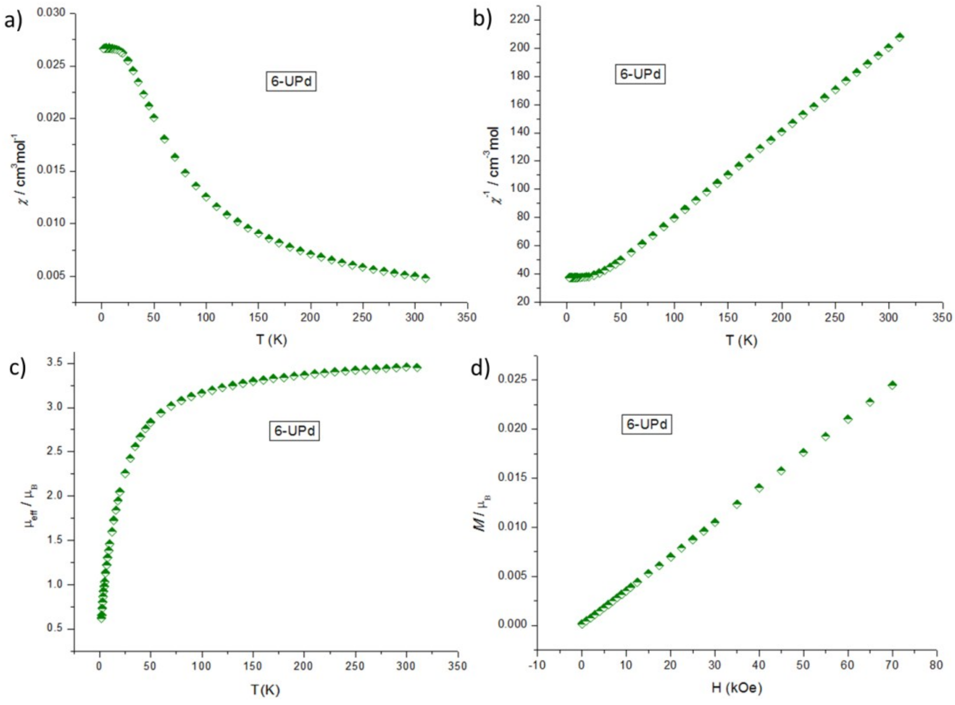

Fig. S24 Variable temperature SQUID data plotted of complex **6-UPd** as χ *vs* T (a), 1/χ *vs* T (b), μ_{eff} *vs* T per uranium ion (c), and field-dependent SQUID magnetization at 1.8 K (d).

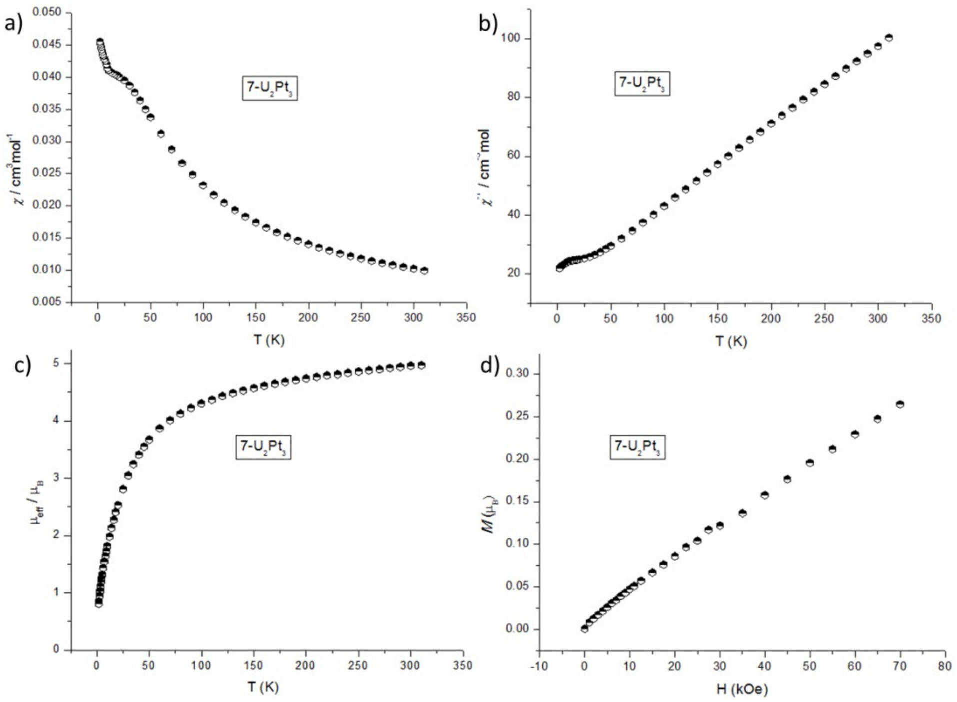

Fig. S25 Variable temperature SQUID data plotted of complex **3-U$_2$Ni$_2$** as χ *vs* T (a), 1/χ *vs* T (b), μ_{eff} *vs* T per uranium ion (c), and field-dependent SQUID magnetization at 1.8 K (d).

5. Computational Section.

The Gaussian09 program suite was used for all quantum-chemical calculations.[S4] We used Becke's 3-parameter hybrid version,[S5] combined with the non-local correlation functional provided by Perdew/Wang,[S6] denoted as B3PW91. The relativistic energy-consistent small-core pseudopotential of the Stuttgart-Köln ECP library was used in combination with its adapted segmented basis set to represent a uranium atom[S7-S9] For phosphorus, chlorine and nickel, palladium and platinum the quasi-relativistic energy-adjusted *ab-initio* pseudopotentials were used, along with their corresponding energy-optimized valence basis sets.[S10,S11] For all the atoms, the 6-31G(d) basis set was used.[S12,S13] In all computations no constraints were imposed on the geometry. A full geometry optimization was performed for each structure using Schlegel's analytical gradient method[S14] and the attainment of the energy minimum was verified by calculating the vibrational frequencies that result in the absence of imaginary eigenvalues. All stationary points have been identified for the minimum (number of imaginary frequencies, Nimag = 0). The NBO analysis[S15] was carried out on the optimized structures using the module included in the Gaussian package. Finally, the Chemcraft graphical program was used for the 3D representations of the structures and the orbital plots.[S16]

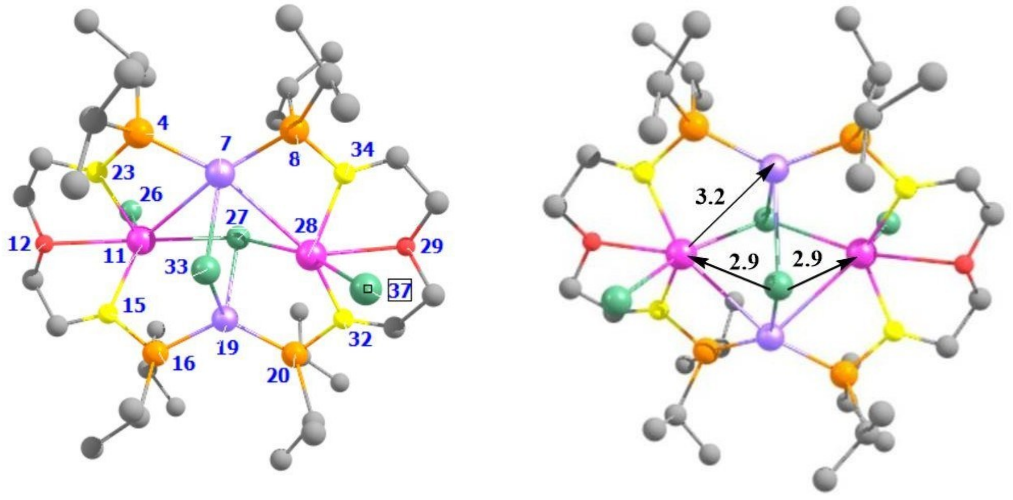

Fig. S26 Optimized structure of complex **3-U_2Ni_2**.

Table S10. The WBI of the complex **3-U_2Ni_2** with regards to the bonding surround the U atoms.

	**WBI**		**WBI**
U28 – Ni7	0.4	U11 – N23	0.8
U11 – Ni19	0.3	U28 – N34	0.8
U11 – Ni7	0.3	U11 – O12	0.3
U28 – Ni19	0.3	U28 – O29	0.3
U11 – Cl27	0.6	Ni7 – P8	0.4
U11 – Cl33	0.6	Ni19 – P20	0.4
U28 – Cl27	0.7	Ni7 – P4	0.4
U28 – Cl33	0.6	Ni19 – P16	0.4

Table S11. NBO analysis summary for complex **3-U_2Ni_2**.

	U(28)				Ni(7)			
1st Bond	11.62%				88.38%			
	s	p	d	f	s	p	d	f
	12.04%	6.94%	27.58%	53.43%	0.19%	0.50%	99.31%	0.00%

	U(11)				Ni(19)			
1st Bond	10.09%				89.91%			
	s	p	d	f	s	p	d	f
	13.07%	6.59%	29.10%	51.23%	0.08%	0.51%	99.41%	0.00%

	U(11)				N(15)			
1st Bond	12.91%				87.09%			
	s	p	d	f	s	p	d	f
	15.05%	5.21%	54.54%	25.17%	45.79%	54.21%	0.01%	0.00%
2nd Bond	12.63%				87.37%			

	s	p	d	f	s	p	d	f
	0.20%	12.62	48.87%	38.29%	0.00%	99.99%	0.01%	0.00%

	U(11)				N(23)			
1st Bond	14.19%				85.81%			
	s	p	d	f	s	p	d	f
	5.29%	3.78%	53.20%	37.70%	45.47%	54.52%	0.01%	0.00%
2nd Bond	12.74%				87.26%			
	s	p	d	f	s	p	d	f
	0.01%	11.98%	40.01%	47.98%	0.03%	99.96%	0.01%	0.00%
	U(28)				N(32)			
1st Bond	14.56%				85.44%			
	s	p	d	f	s	p	d	f
	6.98%	3.49%	53.00%	36.49%	44.81%	55.19%	0.01%	0.00%
2nd Bond	13.29%				86.71%			
	s	p	d	f	s	p	d	f
	0.01%	11.39%	41.38%	47.21%	0.02%	99.97%	0.01%	0.00%
	U(28)				N(34)			
1st Bond	12.94%				87.06%			
	s	p	d	f	s	p	d	f
	13.15%	5.78%	51.67%	29.36%	46.03%	53.96%	0.00%	0.00%
2nd Bond	12.23%				87.77%			
	s	p	d	f	s	p	d	f
	0.03%	13.26%	52.27%	34.43%	0.00%	99.99%	0.01%	0.00%
	U(11)				Cl(26)			
1st Bond	15.47%				84.53%			
	s	p	d	f	s	p	d	f
	14.75%	26.68 %	39.11%	19.45%	53.37%	46.63%	0.00%	0.00%
	U(11)				Cl(27)			
1st Bond	11.84%				88.16%			
	s	p	d	f	s	p	d	f
	14.42%	11.63 %	48.79%	25.16%	30.93%	69.07%	0.00%	0.00%
	U(11)				Cl(33)			

1st Bond	12.07%				87.93%			
	s	p	d	f	s	p	d	f
	15.30%	22.33 %	34.47%	27.89%	32.66%	67.34%	0.00%	0.00%
	U(28)				Cl(27)			
1st Bond	12.66%				87.34%			
	s	p	d	f	s	p	d	f
	14.83%	23.82 %	32.86%	28.49%	34.69%	65.31%	0.00%	0.00%
	U(28)				Cl(33)			
1st Bond	12.30%				87.70%			
	s	p	d	f	s	p	d	f
	11.59%	12.68 %	51.03%	24.70%	32.32%	67.68%	0.00%	0.00%
	U(28)				Cl(37)			
1st Bond	15.90%				84.10%			
	s	p	d	f	s	p	d	f
	14.27%	27.96 %	37.28%	20.49%	54.26%	45.74%	0.00%	0.00%

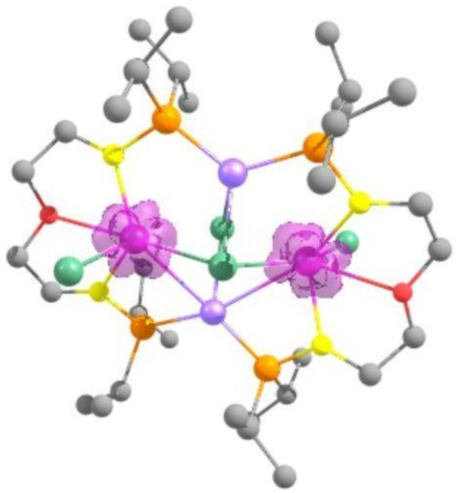

Fig. S27 Unpaired spin density plot for complex **3-U$_2$Ni$_2$**.

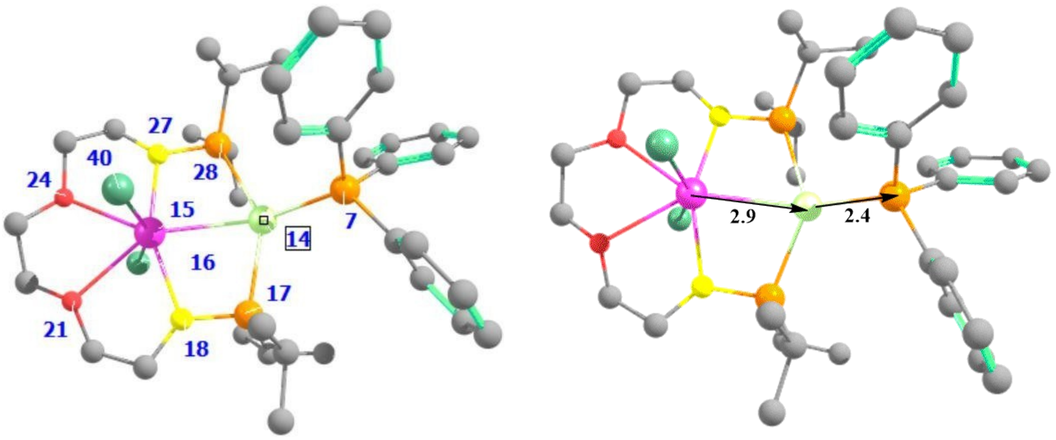

Fig. S28 Optimized structure of complex **6-UPd**.

Table S12. The WBI of the complex **6-UPd** with regards to the bonding surround the U atoms.

Bonding coordinate	WBI
U15 – Pd14	0.4
U15 – Cl16	0.8
U15 – Cl40	0.9
U15 – N18	0.6
U15 – N27	0.6
U15 – O21	0.3
U15 – O24	0.3
Pd14 – P28	0.4
Pd14 – P17	0.4
Pd14 – P7	0.4
N18 – P17	0.9
N27 – P28	0.9

Table S13. NBO analysis summary of the complex **6-UPd**.

	U(15)				P(17)			
1st Bond	13.09%				86.91%			
	s	p	d	f	s	p	d	f
	7.64%	20.38%	52.42%	19.55%	35.01 %	64.90 %	0.09%	0.00%
	U(15)				P(28)			
1st Bond	12.05%				87.95%			
	s	p	d	f	s	p	d	f
	6.78%	22.04%	52.15%	19.04%	35.75 %	64.18 %	0.07%	0.00%
	Pd(14)				P(28)			
1st Bond	25.25%				74.75%			
	s	p	d	f	s	p	d	f
	46.04 %	9.84%	44.12%	0.00%	31.31 %	68.55 %	0.14%	0.00%

Evidently, there is no ‘bond’ reported for U – Pd interactions. Likewise, the O atoms from the ligand are not observed with regards to the NBOs. Having said this, analysis of donations and electron localizations can be analyzed in order to further understand the covalency of the various atomic interactions. Lone pairs are present throughout the system and an outline is as follows: two on each O atom occupying hybrid s/p orbitals, each Cl atom presents four lone pairs with one occupying a hybrid s/p orbital and the others in a p orbital. Additionally, three lone pairs are found in the f orbitals of the U atom and finally two found on the N atoms with one occupying a mixture of s and p orbitals, whilst the other is restricted to a p orbital.

The donation of these lone pairs are evident and firstly a large donation of density (113.13 a.u) is observed from the lone pair on the P(7) atom to the Pd atom. Furthermore the bond between U(15) – P(28) seems to donate to the Rydberg antibonding orbitals of P(7) ~47 a.u. Small donations from the lone pairs on the O atoms (a p to f orbital donation) is seen from the O – U atoms. Additionally, both N and Cl atoms donate relatively more density to the f orbitals of the U atoms too (an p and s/p interaction respectively). For O, N and Cl atoms, the U interactions are purely donations and this would explain the small WBI values.

Concerning the U – Pd bonding or the apparent lack of in the NBO analysis can be further understood due to the covalent nature of the bonding and the donation of density across the U – Pd – P coordinate. Furthermore, there are large donations from the U – P(28) and U – P(17) bonds towards the anti-bonding Rydberg orbitals of the Pd atom. Taking the beta spin orbitals into account, donations

are observed between the P(7) – Pd(14) bond towards the anti-bonding orbitals of the U atom. After the fact the donations are similar regardless of the alpha or beta spin.

Although a large donation is not observed, all lone pairs on the Pd atom donate some density towards the U atom. A total of ~45 a.u. Moreover, when examining the covalency of the interaction/donation between the two atoms, the average value for this interaction is 0.04 which suggests a very high degree of overlap between the d and f orbitals of the two metallic atoms. An example of this interaction is shown in the table below.

Bonding Coordinate	Donation	Orbital Energy Difference	Overall Covalence contribution
Lone Pair (1) Pd(14) to U(15)	2.88	0.96	0.067
Lone Pair (5) Pd(14) to U(15)	0.03	39.84	0.046

This table shows two donations from the Pd atom to the various Ry* orbitals of the U atom. The donations individually are small but I have highlighted that the covalency is very small indicating a strong overlap between the d and f orbitals of the metals. Furthermore, both donations above differ in the difference in energy between the overlapping orbitals. Regardless of the difference in energy, the overlap or covalency is still very strong. This is suggestive of a strong covalent bond between the d and f orbitals of the U and Pd atoms.

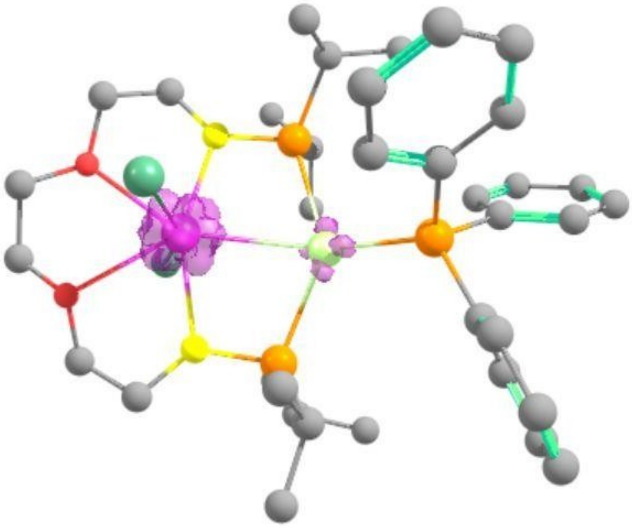

Fig. S29 Unpaired spin density plot for complex **6-UPd**.

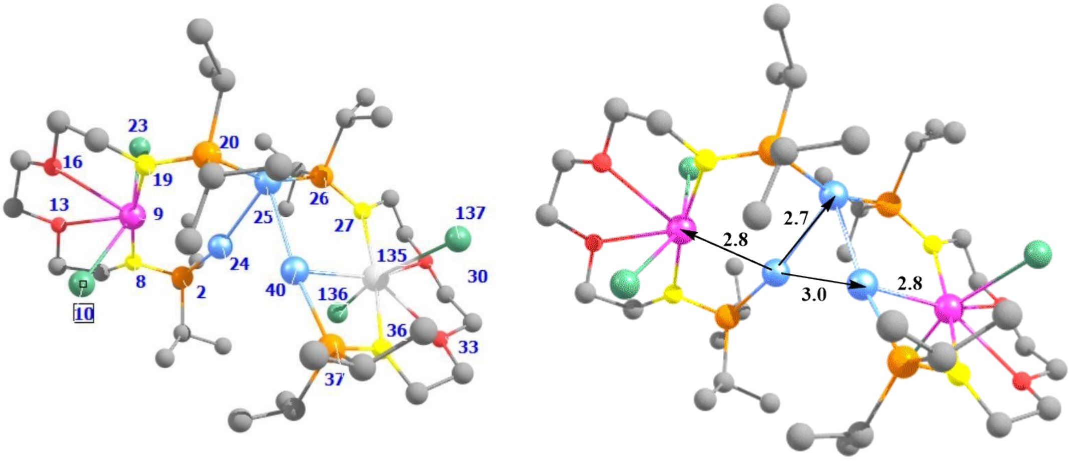

Fig. S30 Optimized structure of complex **7-U$_2$Pt$_3$**.

Table S14. The WBI of the complex **7-U$_2$Pt$_3$** with regards to the bonding surround the U atoms.

**Bonding coordinate**	**W BI**	**Bonding coordinate**	**WBI**
U9 – Pt24	0.8	Pt24 – Pt40	0.2
U9 – O16	0.3	Pt25 – Pt40	0.3
U9 – O13	0.3	U135 – Pt40	0.8
U9 – N8	0.8	U135 – O33	0.3
U9 – N19	0.7	U135 – O30	0.3
U9 – Cl10	1.1	U135 – N27	0.7
U9 – Cl23	1.1	U135 – N36	0.8
Pt24 – P2	0.6	N36 – P37	0.8
Pt25 – P20	0.5	N27 – P26	0.8
Pt24 – Pt25	0.3	Pt40 – P37	0.6
Pt25 – P26	0.5		

Table S15. NBO analysis summary of the complex **7-U$_2$Pt$_3$**.

	U(9)				Cl(10)			
1st Bond	15.96%				84.04%			
	s	p	d	f	s	p	d	f
	10.55%	21.55%	43.54%	24.36%	54.85%	45.15%	0.00%	0.00%
	U(9)				Cl(23)			
1st Bond	15.43%				84.57%			

	s	p	d	f	s	p	d	f
	13.84%	22.59%	40.16%	23.41%	56.68%	43.32%	0.00%	0.00%
	U(135)				Cl(136)			
1st Bond	15.93%				84.07%			
	s	p	d	f	s	p	d	f
	9.60%	21.69%	45.18%	23.53%	54.87%	45.13%	0.00%	0.00%
	U(135)				Cl(137)			
1st Bond	15.67%				84.33%			
	s	p	d	f	s	p	d	f
	8.96%	22.35%	45.53%	23.15%	56.57%	43.43%	0.00%	0.00%
	U(9)				N(8)			
1st Bond	13.54%				86.48%			
	s	p	d	f	s	p	d	f
	12.08%	4.59%	37.13%	46.15%	44.36%	55.63%	0.01%	0.00%
2nd Bond	11.57%				88.43%			
	s	p	d	f	s	p	d	f
	0.00%	8.14%	47.55%	44.29%	0.05%	99.93%	0.02%	0.00%
	U(9)				N(19)			
1st Bond	12.89%				87.11%			
	s	p	d	f	s	p	d	f
	12.22%	5.41%	38.16%	44.18%	46.62%	53.37%	0.01%	0.00%
2nd Bond	9.37%				90.63%			
	s	p	d	f	s	p	d	f
	0.03%	7.33%	50.81%	41.82%	0.01%	99.96%	0.03%	0.00%

	U(135)				N(27)			
1st Bond	13.05%				86.95%			
	s	p	d	f	s	p	d	f
	7.71%	5.65%	42.63%	43.98%	46.99%	53.00%	0.01%	0.00%
2nd Bond	9.71%				90.29%			
	s	p	d	f	s	p	d	f
	0.07%	7.81%	50.67%	41.44%	0.21%	99.76%	0.02%	0.00%
	U(135)				N(36)			
1st Bond	14.18%				85.82%			
	s	p	d	f	s	p	d	f
	5.06%	4.03%	43.67%	47.21%	44.61%	55.38%	0.01%	0.00%
2nd Bond	11.63%				88.37%			
	s	p	d	f	s	p	d	f
	0.03%	7.82%	47.49%	44.65%	0.06%	99.93%	0.02%	0.00%
	U(9)				Pt(24)			
1st Bond	8.96%				91.04%			
	s	p	d	f	s	p	d	f
	26.02%	12.11%	44.88%	16.98%	6.52%	0.24%	93.25%	0.00%
	P(20)				Pt(25)			
1st Bond	45.26%				54.74%			
	s	p	d	f	s	p	d	f
	34.65%	65.02%	0.33%	0.00%	3.76%	1.29%	94.95%	0.00%
	P(37)				Pt(40)			
1st	36.63%				63.37%			

Bond								
	s	p	d	f	s	p	d	f
	22.28%	76.94%	0.78%	0.00%	22.48%	77.46%	0.06%	0.00%
	P(26)				Pt(40)			
1st Bond	5.37%				94.63%			
	s	p	d	f	s	p	d	f
	32.85%	66.88%	0.27%	0.00%	3.01%	0.31%	96.68%	0.00%

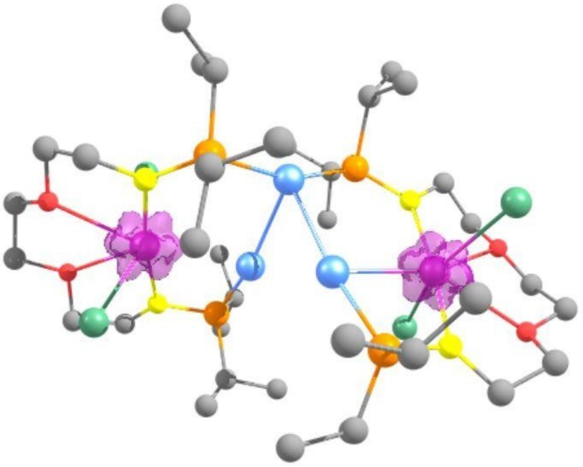

Fig. S31 Unpaired spin density plot for complex **7-U_2Pt_3**.

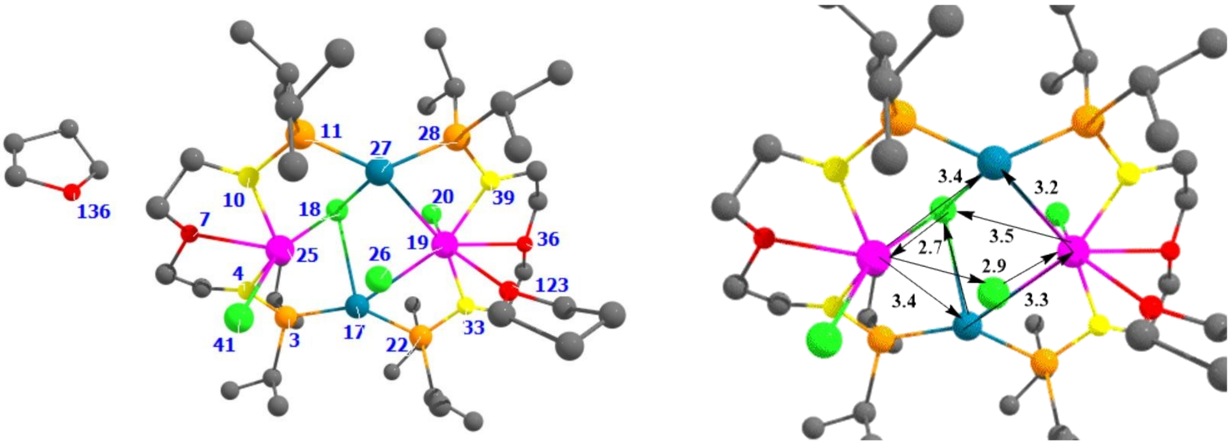

Fig. S32 Optimized structure of complex **4-U_2Pd_2**.

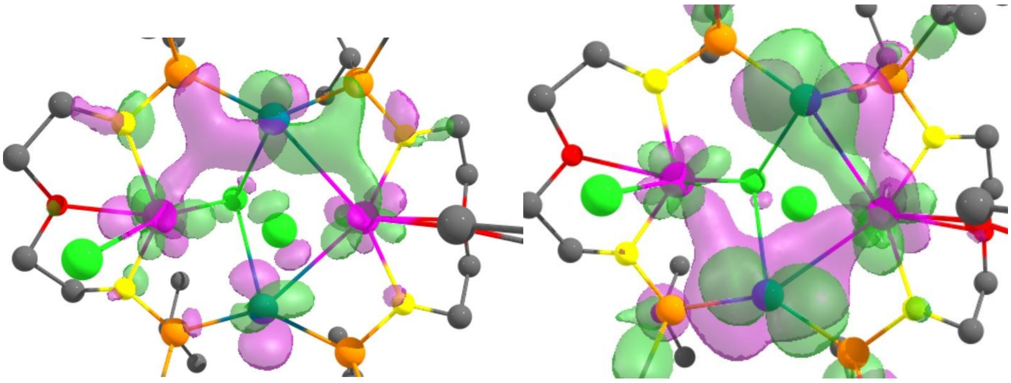

Fig. S33 Bonding U-Pd Molecular Orbital depiction complex **4-U_2Pd_2**.

Table S16. The WBI of the complex **4-U_2Pd_2** with regards to the bonding surround the U atoms.

**Bonding coordinate**	**WBI**	**Bonding coordinate**	**WBI**
U25 – O7	0.3	U19 – Pd27	0.3
U25 – N10	0.8	U19 – Pd17	0.3
U25 – N4	0.8	U19 – N33	0.8
U25 – Cl41	1.1	U19 – N39	0.8
U25 – Cl18	0.9	U19 – O36	0.3
U25 – Cl26	0.6	U19 – Cl18	0.3
U25 – O136	0.0	U19 – Cl26	0.6
U25 – Pd27	0.2	U19 – O123	0.3
U25 – Pd17	0.2	U19 – Cl20	1.1
Pd27 – P28	0.5	Pd17 – P22	0.4
Pd27 – P11	0.4	Pd17– P3	0.4
N10 – P11	0.8	N4 – P3	0.8
N39 – P28	0.8	N33 – P22	0.8

Table S17. NBO analysis summary of the complex **4-U_2Pd_2**.

	N(4)				U(25)			
1st Bond	86.04%				13.96%			
	s	p	d	f	s	p	d	f
	45.84%	54.16%	0.01%	0.00%	5.83%	4.51%	52.76%	36.86%
2nd Bond	87.36%				12.64%			
	s	p	d	f	s	p	d	f
	0.01%	99.98%	0.01%	0.00%	0.14%	11.84%	43.53%	44.47%

	N(10)	U(25)

1st Bond	86.14%				13.86%			
	s	p	d	f	s	p	d	f
	45.61%	54.38%	0.01%	0.00%	7.02%	3.59%	51.43%	37.93%
2nd Bond	87.80%				12.20%			
	s	p	d	f	s	p	d	f
	0.01%	99.98%	0.01%	0.00%	0.18%	12.80%	46.80%	40.21%

	Cl(18)				U(25)			
1st Bond	87.15%				12.85%			
	s	p	d	f	s	p	d	f
	25.53%	74.47%	0.00%	0.00%	7.50%	16.77%	39.48%	36.25%
2nd Bond	92.55%				7.45%			
	s	p	d	f	s	p	d	f
	33.29%	66.71%	0.00%	0.00%	1.59%	14.00%	53.42%	30.99%

	U(19)				Cl(20)			
1st Bond	15.37%				84.63%			
	s	p	d	f	s	p	d	f
	9.33%	15.10%	59.43%	16.13%	54.92%	45.08%	0.00%	0.00%

	U(19)				Cl(26)			
1st Bond	12.06%				87.94%			
	s	p	d	f	s	p	d	f
	3.93%	11.73%	58.13%	26.19%	35.54%	64.46%	0.00%	0.00%

	U(19)				N(33)			
1st Bond	13.43%				86.57%			
	s	p	d	f	s	p	d	f
	4.27%	3.24%	48.60%	43.85%	45.19%	54.81%	0.01%	0.00%
2nd Bond	12.89%				87.11%			
	s	p	d	f	s	p	d	f
	0.02%	4.80%	45.30%	49.87%	0.06%	99.93%	0.01%	0.00%

	U(19)				N(39)			
1st Bond	13.36%				86.64%			
	s	p	d	f	s	p	d	f
	4.15%	3.58%	50.59%	41.66%	45.21%	54.79%	0.01%	0.00%
2nd Bond	12.24%				87.76%			
	s	p	d	f	s	p	d	f
	0.07%	5.64%	48.57%	45.71%	0.00%	99.99%	0.01%	0.00%

	U(25)				Cl(26)			
1st Bond	13.12%				86.88%			
	s	p	d	f	s	p	d	f
	12.11%	12.04%	50.26%	25.59%	35.69%	64.31%	0.00%	0.00%

	U(25)				Cl(41)			
1st Bond	16.10%				83.90%			
	s	p	d	f	s	p	d	f
	15.60%	27.51%	35.91%	20.98%	53.19%	46.81%	0.00%	0.00%

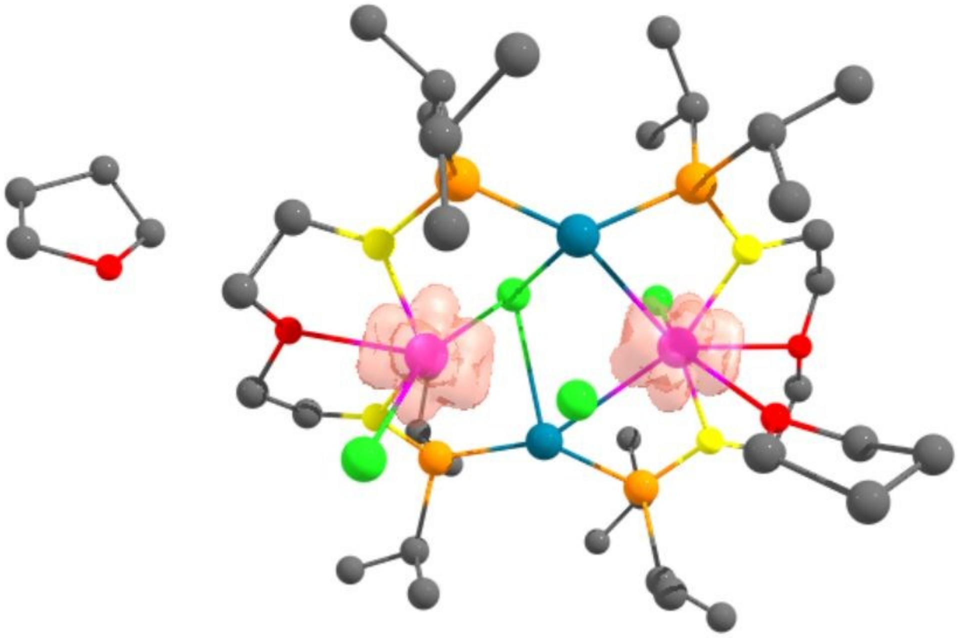

Fig. S34 Unpaired density plot of complex **4-U$_2$Pd$_2$**.

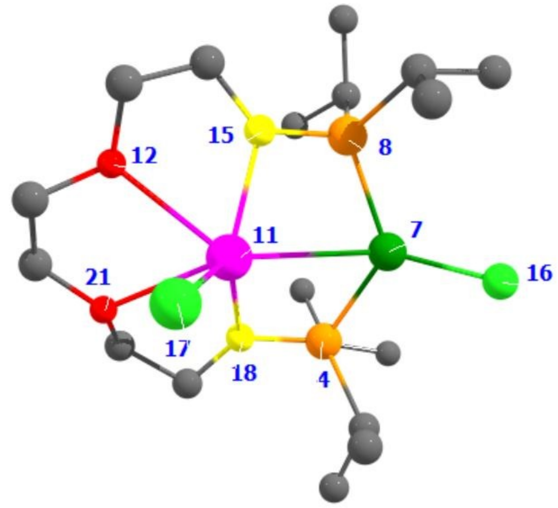
12
15
8
11
7
16
21
17
18
4

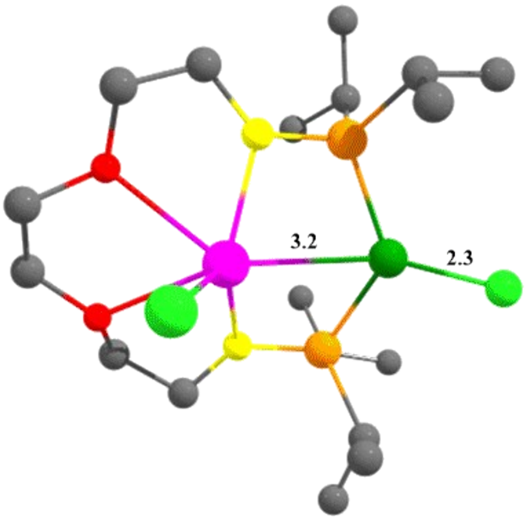
3.2
2.3

Fig. S35 Optimized structure of complex **5-UNi**.

SI – Coordinates:

Complex 3 3-U_2Ni_2

SCF Energy: -3014.75479135,

C	6.543413	-0.060730	-0.229981	-0.389372
C	5.121032	-0.480003	-0.612451	-0.177394
C	4.794041	-0.105406	-2.058715	-0.394893
P	3.685450	0.096666	0.504552	10.394158
C	4.477792	0.233961	2.228359	-0.168361
C	4.850349	-1.138206	2.786917	-0.404274
Ni	1.848238	-1.097266	0.419661	9.720762
P	1.761374	-3.097377	-0.411229	10.383076
C	2.615116	-4.433969	0.630181	-0.162612
C	2.047899	-4.442176	2.048424	-0.406008
U	1.170394	1.934233	-0.324684	61.097953
O	2.453133	4.113254	0.184772	-0.522163
C	1.693316	5.273320	-0.153865	-0.011519
C	0.356243	5.065965	0.527069	-0.101743
N	-0.075168	3.700406	0.275208	-0.702608
P	-1.727082	3.181876	0.296194	10.372577
C	-2.577394	4.358366	-0.923411	-0.159245
C	-4.100599	4.270407	-0.847281	-0.395984
Ni	-1.894338	1.076799	-0.190917	9.744450
P	-3.743463	-0.109080	-0.155035	10.401901
C	-5.046580	0.031489	1.214783	-0.165257
C	-4.405126	-0.276727	2.566485	-0.414282
N	3.366938	1.743635	0.082282	-0.705583
C	4.378617	2.783739	0.178790	-0.093288
C	3.781742	4.080593	-0.336175	-0.016028
Cl	1.634343	2.750619	-2.807138	9.518509
Cl	-0.417461	-0.057369	-1.734105	9.795902
U	-1.166617	-1.990000	0.225118	61.029528
O	-2.385024	-4.130962	-0.522791	-0.525152
C	-3.709157	-4.180836	0.011635	-0.015923
C	-4.335580	-2.857332	-0.386715	-0.095672
N	-3.360638	-1.802314	-0.163071	-0.712281
Cl	0.208760	0.104436	1.706002	9.809704
N	0.134610	-3.648133	-0.558398	-0.698710
C	-0.264252	-4.975868	-0.994605	-0.105414

C	-1.594750	-5.305729	-0.344969	-0.009152
Cl	-1.523167	-3.066950	2.628129	9.515228
C	-2.414436	3.721014	1.990812	-0.170532
C	-2.558700	5.225310	2.239968	-0.391000
C	2.564631	-3.324122	-2.123062	-0.170273
C	2.752501	-4.765319	-2.606814	-0.392600
C	-4.745970	0.216848	-1.747441	-0.174830
C	-6.153659	-0.382220	-1.826165	-0.391658
C	3.553691	0.977822	3.191623	-0.411206
C	-1.608368	3.052931	3.104193	-0.404677
C	-2.075942	4.082234	-2.339812	-0.413337
C	1.812016	-2.501590	-3.167468	-0.402961
C	4.134853	-4.270749	0.634408	-0.396689
C	-3.909391	-0.165882	-2.968721	-0.402980
C	-5.739489	1.392834	1.222663	-0.396201
H	4.712322	2.922179	1.219053	0.130761
H	5.271975	2.560486	-0.419177	0.125835
H	4.341402	4.955939	0.020109	0.125046
H	3.735190	4.093463	-1.432489	0.183256
H	1.585614	5.325034	-1.245398	0.179577
H	2.207556	6.172771	0.211313	0.124133
H	0.476053	5.252115	1.605322	0.135067
H	-0.350642	5.816447	0.146987	0.123147
H	0.459548	-5.755033	-0.718573	0.121623
H	-0.381904	-5.018425	-2.088589	0.137544
H	-2.089330	-6.160808	-0.826028	0.124026
H	-1.478414	-5.502497	0.729223	0.178534
H	-3.651622	-4.291533	1.101994	0.183677
H	-4.251111	-5.033496	-0.419340	0.123935
H	-4.621652	-2.910254	-1.447643	0.133381
H	-5.260233	-2.717706	0.191556	0.126600
H	5.056281	-1.571857	-0.519325	0.144276
H	7.258524	-0.524697	-0.920893	0.131525
H	6.821974	-0.374055	0.779991	0.132953
H	6.692849	1.020776	-0.296481	0.126126
H	4.860012	0.974658	-2.220007	0.152809
H	3.780123	-0.407303	-2.336345	0.171759
H	5.494871	-0.592404	-2.747427	0.123914
H	5.395820	0.820536	2.094061	0.129719
H	2.616859	0.432561	3.341360	0.189215
H	3.304128	1.981310	2.834195	0.147572
H	4.041365	1.083589	4.168702	0.130592
H	5.326863	-1.031195	3.769108	0.132715
H	5.549209	-1.680796	2.143018	0.133954

H	3.958124	-1.759226	2.917106	0.171450
H	-3.417088	3.276241	1.987597	0.142031
H	-0.581024	3.432947	3.130116	0.139849
H	-1.555472	1.969281	2.972872	0.185408
H	-2.063932	3.258779	4.080632	0.128882
H	-3.092828	5.390387	3.184224	0.130939
H	-3.121560	5.740312	1.456309	0.128336
H	-1.586807	5.717217	2.333871	0.127552
H	-2.272443	5.372825	-0.632738	0.124491
H	-2.383134	3.084950	-2.673815	0.172248
H	-0.985968	4.125112	-2.411187	0.185066
H	-2.493301	4.816231	-3.040551	0.122566
H	-4.554286	4.961122	-1.568520	0.131962
H	-4.494731	4.521001	0.141942	0.129690
H	-4.442740	3.261521	-1.096458	0.153362
H	3.556527	-2.875095	-1.981905	0.142134
H	2.318357	-2.566457	-4.138314	0.129566
H	1.742555	-1.447766	-2.889843	0.183831
H	0.789596	-2.872216	-3.298161	0.146628
H	1.796368	-5.246675	-2.832079	0.130716
H	3.288182	-5.396736	-1.892945	0.127163
H	3.334298	-4.764136	-3.537204	0.131349
H	2.372171	-5.393157	0.153230	0.125304
H	4.417949	-3.307302	1.066014	0.154454
H	4.573670	-4.334686	-0.365935	0.128686
H	4.596386	-5.055154	1.246784	0.131888
H	2.507196	-5.247371	2.635644	0.122134
H	0.964764	-4.585636	2.061202	0.180596
H	2.249195	-3.495023	2.560698	0.165540
H	-4.845361	1.309979	-1.736979	0.148387
H	-3.707485	-1.242488	-2.991376	0.139800
H	-2.944845	0.347466	-2.976326	0.193362
H	-4.444461	0.092875	-3.890673	0.128796
H	-6.665505	0.011545	-2.713335	0.132507
H	-6.775398	-0.138959	-0.960674	0.129882
H	-6.131733	-1.470140	-1.927102	0.127514
H	-5.797039	-0.740286	0.996822	0.125676
H	-6.489915	1.428355	2.021644	0.130601
H	-6.248573	1.620664	0.281680	0.130543
H	-5.020477	2.194558	1.411627	0.148961
H	-3.623308	0.453505	2.803129	0.164823
H	-3.946404	-1.268788	2.590608	0.186966
H	-5.158284	-0.232884	3.363039	0.123027

Complex 4 4-U_2Pd_2

148
SCF Energy: -3393.53670131

C	3.320602	5.121915	0.113195
C	4.453904	5.527088	1.058937
P	3.989843	6.261637	2.750020
N	3.075574	5.086113	3.631796
C	1.746099	4.654398	3.214888
C	1.442595	3.342228	3.903091
O	1.811621	3.551844	5.270014
C	1.621015	2.411711	6.116483
C	2.004402	2.881662	7.504967
N	3.207337	3.697335	7.412705
P	4.119322	4.139068	8.817813
C	2.880492	4.834513	10.084915
C	2.124635	6.008534	9.462254
C	5.445503	4.380081	1.253363
C	2.695052	7.576357	2.324538
C	2.171796	8.235160	3.600535
Pd	5.927661	6.945649	3.890767
Cl	4.258344	7.184300	6.429866
U	7.681745	7.721221	6.582812
Cl	6.418137	10.056942	6.735737
C	3.230062	8.623710	1.350082
P	7.867590	8.084404	3.376426
C	9.034009	7.079319	2.262345
C	9.408053	5.768305	2.947998
U	4.117567	4.583490	5.553766
Cl	6.933106	4.993888	5.837489
Pd	5.796459	5.753217	8.330017
P	7.172692	6.960203	9.705657
C	8.234789	5.885413	10.857339
C	9.055955	4.903554	10.028584
C	7.631356	9.673934	2.374991
C	7.099115	9.342219	0.979401
N	8.764559	8.537963	4.786249
C	9.897394	9.451455	4.767835
C	9.861387	10.293245	6.028457
O	9.631003	9.374160	7.098408
C	9.477893	9.986530	8.382375
C	9.218657	8.846178	9.345228
N	8.231362	7.950310	8.765092
C	6.711311	10.639867	3.117401
Cl	4.878489	2.200455	4.636209
C	4.667738	2.498336	9.584261

C	5.564114	1.746634	8.602269
C	5.381108	2.740606	10.914830
C	6.340024	8.148604	10.924082
C	5.603492	7.389306	12.028517
C	5.396044	9.085417	10.174652
C	1.934259	3.850294	10.778055
C	10.277861	7.812598	1.756445
C	9.104378	6.608950	11.888406
H	0.980994	5.395526	3.496254
H	1.663923	4.507282	2.132262
H	0.380939	3.072418	3.853386
H	2.058264	2.530610	3.492402
H	2.278973	1.604633	5.769020
H	0.571918	2.098335	6.061526
H	1.170215	3.463450	7.924236
H	2.136215	1.997633	8.146266
H	9.884073	10.129889	3.904437
H	10.854356	8.909712	4.724207
H	10.810628	10.821996	6.196266
H	9.035598	11.014759	5.998333
H	8.626308	10.676326	8.342131
H	10.393924	10.537596	8.638017
H	10.167405	8.319450	9.535695
H	8.897937	9.275434	10.304932
H	4.996283	6.370250	0.608109
H	3.726941	4.930689	-0.887995
H	2.548286	5.890212	0.007974
H	2.841038	4.193800	0.439056
H	4.981085	3.529464	1.762119
H	6.302341	4.696848	1.856583
H	5.817405	4.030818	0.281805
H	1.870778	7.034309	1.841243
H	2.968860	8.776462	4.118757
H	1.766813	7.505426	4.306768
H	1.375436	8.948474	3.353324
H	2.451604	9.365318	1.132514
H	3.548457	8.194172	0.395215
H	4.081270	9.155239	1.785792
H	3.559320	5.240552	10.845357
H	1.431778	5.669197	8.684939
H	2.808340	6.725979	8.999796
H	1.537270	6.531594	10.227097
H	1.385454	4.371114	11.573178
H	2.459854	3.012394	11.244763

H	1.189324	3.438413	10.092150
H	3.761546	1.905331	9.766122
H	6.467181	2.326520	8.379487
H	5.061785	1.546756	7.652257
H	5.876124	0.786647	9.032444
H	5.717690	1.789023	11.344318
H	4.743325	3.230516	11.657372
H	6.263391	3.371981	10.763566
H	8.388798	6.842292	1.406139
H	10.005379	5.140480	2.274940
H	8.519802	5.205488	3.248095
H	10.002039	5.959525	3.847720
H	10.974496	8.027069	2.572995
H	10.045583	8.756796	1.254753
H	10.813492	7.184391	1.033541
H	8.620337	10.139246	2.266842
H	6.199597	8.722176	1.048070
H	7.831342	8.809478	0.365439
H	6.833295	10.264959	0.449825
H	6.563941	11.550421	2.522933
H	7.109302	10.925311	4.093428
H	5.733029	10.184386	3.301822
H	7.462389	5.314242	11.389771
H	9.797953	5.433845	9.422684
H	8.409788	4.338294	9.349215
H	9.588071	4.195016	10.675669
H	9.528932	5.881826	12.592330
H	8.548129	7.343328	12.478361
H	9.944776	7.124979	11.415409
H	7.141748	8.743481	11.383018
H	5.064619	8.094849	12.672347
H	6.271820	6.804452	12.667553
H	4.865590	6.705950	11.596009
H	4.591517	8.519139	9.693604
H	5.904433	9.647071	9.387965
H	4.942341	9.800854	10.872332
O	9.908230	6.092240	6.592199
C	11.230308	6.656191	6.579815
C	12.116414	5.587559	7.195410
C	11.512825	4.316635	6.592877
C	10.019239	4.644096	6.490964
H	11.693631	3.424897	7.198914
H	11.935496	4.134387	5.598933
H	13.173019	5.723979	6.947940

H	12.015332	5.591563	8.285992
H	11.529488	6.860087	5.541398
H	11.192975	7.597709	7.126911
H	9.434971	4.218637	7.307518
H	9.576080	4.320757	5.548467
O	-1.812045	2.543108	5.253148
C	-2.375957	3.789368	5.662384
C	-3.503363	3.437277	6.626387
C	-4.033457	2.144437	6.000676
C	-2.748493	1.486995	5.489449
H	-1.582176	4.394462	6.111525
H	-2.766166	4.331085	4.786345
H	-3.102429	3.244647	7.627960
H	-4.257757	4.224756	6.706304
H	-4.579221	1.509650	6.704011
H	-4.705216	2.376838	5.166624
H	-2.900257	0.923416	4.561065
H	-2.325105	0.801062	6.236377

Complex 5: **5-UNi**

68

SCF Energy: -1661.13486617,

C	12.382946	8.856300	7.382619
C	10.909635	9.239199	7.232094
C	10.274981	9.581062	8.580435
P	9.829641	7.961826	6.348993
C	10.801041	7.586010	4.771127
C	11.086824	8.858468	3.970376
Ni	7.745802	8.909439	6.031209
P	5.695602	7.818565	5.969520
C	4.309535	9.040841	6.359811
C	4.250425	10.166520	5.323929
U	7.626634	6.664242	8.231532
O	6.335767	4.472435	8.483491
C	4.930889	4.784208	8.564496
C	4.588925	5.566973	7.297289
N	5.631435	6.549985	7.094163
Cl	8.058111	11.050712	5.414579
Cl	7.223481	7.198453	10.846011
N	9.720768	6.528766	7.255316
C	10.786275	5.671695	7.731410
C	10.184166	4.305492	8.031570
O	8.981284	4.507941	8.796134

C	8.279525	3.304756	9.097044
C	6.874603	3.668792	9.532169
C	5.274145	7.164611	4.242027
C	6.162503	5.961817	3.923929
C	10.069127	6.546851	3.925638
C	3.795487	6.839943	4.012108
C	4.508866	9.590939	7.772426
H	11.259104	6.056155	8.649674
H	11.597668	5.516107	7.002466
H	9.915112	3.791530	7.098055
H	10.875884	3.674050	8.605168
H	8.792907	2.751078	9.894415
H	8.237611	2.674457	8.197745
H	6.279846	2.754032	9.657330
H	6.876857	4.231220	10.475515
H	4.754544	5.387527	9.465290
H	4.356784	3.850807	8.633851
H	4.513750	4.857621	6.457777
H	3.586315	6.001238	7.438606
H	10.821273	10.121527	6.585491
H	12.938342	9.683953	7.840005
H	12.865144	8.634095	6.425895
H	12.508834	7.985925	8.034662
H	10.255156	8.713957	9.252454
H	9.251706	9.952581	8.458146
H	10.847294	10.368685	9.084181
H	11.750605	7.152527	5.115075
H	9.173625	6.983827	3.472019
H	9.763476	5.678682	4.517744
H	10.713564	6.196224	3.110689
H	11.583916	8.601293	3.027168
H	11.739271	9.552866	4.506459
H	10.162094	9.394727	3.732904
H	5.560212	7.993107	3.580088
H	3.647909	6.500514	2.979402
H	3.143539	7.703778	4.164861
H	3.448078	6.035801	4.668405
H	5.902254	5.100428	4.547584
H	7.218728	6.183473	4.092371
H	6.039994	5.664480	2.875753
H	3.376067	8.462039	6.330687
H	5.413042	10.208102	7.827285
H	4.594911	8.793150	8.516947
H	3.660475	10.225228	8.054822

H	3.454891	10.873498	5.587559	
H	4.040517	9.800358	4.314190	
H	5.193961	10.721771	5.291382	

Complex 7 7-U_2Pt_3

SCF Energy: -3338.83668418,

C	10.057844	6.775036	14.726637	-0.395717
P	7.328346	5.975917	15.269862	10.342971
C	8.163749	4.360423	15.735874	-0.169855
C	7.169989	3.207417	15.618710	-0.404013
C	8.724747	7.254045	15.311981	-0.186958
C	8.263231	8.541837	14.631102	-0.395638
C	8.792020	4.467587	17.127735	-0.414455
N	6.758928	5.832364	13.675118	-0.695588
U	4.556887	6.388614	13.793777	61.362183
Cl	3.878487	3.797101	13.466842	9.467958
C	7.571617	5.480420	12.528480	-0.099006
C	6.641659	4.993588	11.432853	-0.016173
O	5.570118	5.940608	11.377246	-0.515758
C	4.675398	5.738087	10.293346	0.001694
C	3.557983	6.738901	10.454780	-0.014080
O	2.982363	6.475025	11.726014	-0.522153
C	1.723591	7.089943	11.988629	0.007531
C	1.289910	6.559518	13.339833	-0.130995
N	2.328822	6.840848	14.334725	-0.695723
P	1.828158	7.244725	15.905795	10.315453
C	0.601477	5.928005	16.442367	-0.171459
C	0.074575	6.210043	17.849102	-0.417259
Cl	4.856863	8.959374	13.039440	9.461571
Pt	5.475542	6.572406	16.434941	60.084808
Pt	3.430899	7.960628	17.451507	59.419757
P	4.575903	9.363874	18.938706	10.340112
N	5.159467	8.848304	20.456315	-0.707775
C	5.648502	9.896732	21.355731	-0.121710
C	6.742536	9.358995	22.253761	-0.007325
O	6.190324	8.212805	22.900817	-0.521293
C	7.004769	7.636717	23.912981	-0.010199
C	6.190458	6.529894	24.534159	-0.004639
O	5.868882	5.618573	23.493416	-0.513086
C	5.065304	4.514535	23.917211	-0.010336
C	4.927826	3.609158	22.707470	-0.105984
N	4.618439	4.444711	21.565408	-0.700778

P	3.897306	3.868967	20.135508	10.288058
C	2.233737	3.124935	20.666625	-0.169683
C	1.294482	2.829561	19.498632	-0.401520
Pt	3.884067	5.694565	18.790626	59.680921
C	4.921610	2.366575	19.627514	-0.167798
C	4.421035	1.673611	18.359214	-0.413890
C	3.185204	10.644965	19.193831	-0.196682
C	2.098929	10.033098	20.081962	-0.400274
C	5.965897	10.333792	18.119675	-0.173322
C	7.223812	9.472635	18.052224	-0.404350
C	3.604193	12.033584	19.686365	-0.397334
C	0.810697	8.863044	15.874803	-0.193268
C	-0.621377	8.811694	15.333035	-0.397261
C	1.629685	9.951781	15.177614	-0.393389
C	1.244504	4.548944	16.315236	-0.412906
C	5.535661	10.847916	16.744989	-0.417477
C	6.382569	2.783019	19.491095	-0.402385
C	1.540696	4.031096	21.682736	-0.395267
H	8.295488	4.679141	12.742318	0.122993
H	8.142542	6.344574	12.155198	0.137107
H	7.151181	4.956503	10.459962	0.121174
H	6.224365	4.007677	11.675301	0.183261
H	4.276843	4.714841	10.328121	0.178587
H	5.196867	5.897610	9.339085	0.132901
H	3.937216	7.768790	10.422875	0.191241
H	2.808796	6.592865	9.664724	0.131463
H	1.009475	6.805393	11.202287	0.121221
H	1.845140	8.181314	12.003001	0.167936
H	0.324094	7.017243	13.583703	0.116531
H	1.119620	5.476153	13.243919	0.173574
H	8.863609	7.445309	16.384325	0.172946
H	9.975598	6.544161	13.660226	0.130405
H	10.461048	5.895752	15.235213	0.135294
H	10.802229	7.574647	14.824545	0.135568
H	9.007781	9.332642	14.783103	0.125146
H	7.304185	8.891613	15.017450	0.202134
H	8.136766	8.400353	13.553672	0.154465
H	8.960224	4.216673	14.992304	0.130418
H	7.656260	2.264449	15.896556	0.123138
H	6.774317	3.107198	14.604876	0.173326
H	6.313167	3.361799	16.280481	0.178779
H	8.063514	4.786801	17.878422	0.206369
H	9.620696	5.181265	17.160429	0.134796
H	9.187165	3.493006	17.437200	0.132756

H	0.748156	9.107277	16.945864	0.167571
H	1.767015	9.729731	14.115617	0.165119
H	2.634760	10.047870	15.602083	0.179713
H	1.125886	10.922132	15.265082	0.131390
H	-1.131300	9.753817	15.569550	0.140722
H	-1.217700	8.004037	15.766284	0.139842
H	-0.641999	8.707509	14.244013	0.134639
H	-0.226554	5.995891	15.723442	0.135915
H	2.096492	4.454119	17.000125	0.186460
H	1.616218	4.352725	15.306600	0.184127
H	0.516256	3.770626	16.571241	0.129799
H	-0.644572	5.437311	18.145781	0.138058
H	-0.436290	7.175703	17.922079	0.137740
H	0.898135	6.207980	18.574057	0.203798
H	6.158794	11.194737	18.773154	0.131411
H	8.041446	10.040492	17.592675	0.131069
H	7.544826	9.129463	19.038703	0.168840
H	7.039269	8.579709	17.441261	0.187933
H	6.356295	11.406998	16.280617	0.140606
H	5.278692	10.022250	16.072384	0.221445
H	4.674572	11.522510	16.800762	0.136619
H	2.778365	10.765047	18.178630	0.165979
H	1.248675	10.721057	20.165937	0.129280
H	1.731263	9.083989	19.677546	0.175097
H	2.469116	9.821213	21.089124	0.181340
H	2.733693	12.700598	19.665896	0.138743
H	3.964783	12.008665	20.717885	0.137039
H	4.377346	12.492026	19.062889	0.138049
H	2.509126	2.176732	21.152684	0.132914
H	0.402411	2.307654	19.866028	0.129597
H	0.967362	3.762112	19.034626	0.175403
H	1.742543	2.211197	18.718721	0.142575
H	0.594528	3.574587	21.998239	0.123551
H	2.147884	4.215499	22.570270	0.153332
H	1.317508	5.010521	21.248782	0.195784
H	4.819122	1.675598	20.477689	0.134017
H	7.005463	1.902257	19.294241	0.123419
H	6.499774	3.476555	18.655674	0.173173
H	6.764442	3.288223	20.381087	0.176417
H	5.144705	0.911702	18.046530	0.136307
H	3.464242	1.166741	18.505537	0.132929
H	4.313086	2.382288	17.531546	0.192213
H	4.146136	2.865125	22.925917	0.123463
H	5.868827	3.056677	22.558637	0.143667

H	5.558995	3.999721	24.753248	0.121908
H	4.087362	4.894317	24.242138	0.177266
H	5.267734	6.932332	24.974260	0.181949
H	6.776836	6.021845	25.312696	0.133505
H	7.247510	8.393658	24.671644	0.132707
H	7.931105	7.246284	23.471220	0.189538
H	7.024657	10.103914	23.011396	0.122575
H	7.627724	9.058187	21.678603	0.182033
H	6.069909	10.757798	20.818924	0.121500
H	4.837531	10.276224	21.995601	0.160316
U	5.044236	6.639076	21.181107	61.393984
Cl	2.957676	7.334890	22.737047	9.477328
Cl	7.651254	6.279350	20.614980	9.456693

Complex 6 6-UPd

SCF Energy: -2319.36345369,

C	-1.816143	-0.714730	2.732197
C	-2.883013	-0.928328	1.849927
C	-3.975113	-1.700660	2.265119
C	-4.001602	-2.236472	3.551523
C	-2.948058	-1.999369	4.435046
C	-1.853993	-1.240033	4.023575
P	-2.797402	-0.095405	0.197131
C	-3.728955	1.484468	0.501021
C	-3.952071	1.981800	1.789518
C	-4.634833	3.185338	1.969853
C	-5.102481	3.900938	0.869940
C	-4.886162	3.408652	-0.418312
C	-4.200455	2.211317	-0.602506
Pd	-0.582282	-0.017274	-0.673697
U	2.190754	0.092821	0.307810
Cl	3.439137	0.042196	-2.257970
P	0.116760	2.386238	-0.676462
N	1.728317	2.314119	-0.184695
C	2.727818	3.360414	-0.072292
C	4.127147	2.746833	-0.201971
O	4.224346	1.593245	0.664015
C	5.463753	0.882126	0.580767
C	5.316294	-0.437427	1.322917
O	4.325712	-1.191887	0.629890
C	4.009358	-2.515736	1.096233
C	2.993760	-3.113907	0.113468

N	1.887466	-2.184756	-0.040963
P	0.345086	-2.404369	-0.689081
C	0.374747	-3.016480	-2.494264
C	1.512531	-3.997579	-2.779935
C	-0.811560	3.582672	0.463299
C	-0.443698	5.052462	0.238843
C	-0.563002	3.168766	1.912597
C	0.007352	3.198909	-2.381458
C	0.820153	2.406920	-3.401993
C	-1.444547	3.360341	-2.829450
C	-0.365819	-3.937970	0.187294
C	-0.163645	-3.849657	1.698408
C	-1.819875	-4.250856	-0.159199
Cl	1.939270	-0.037267	3.059345
C	-3.995877	-0.979266	-0.914704
C	-5.384117	-0.887740	-0.730305
C	-6.253057	-1.546188	-1.597478
C	-5.749387	-2.288570	-2.665925
C	-4.373048	-2.368510	-2.866958
C	-3.501369	-1.716174	-1.995681
C	0.410179	-1.844002	-3.473927
H	2.656037	3.883679	0.895199
H	2.662242	4.133114	-0.856506
H	4.296377	2.408186	-1.230715
H	4.895000	3.476843	0.090690
H	6.266179	1.475490	1.040709
H	5.695686	0.686846	-0.473922
H	6.277895	-0.969272	1.306567
H	5.006002	-0.272993	2.364244
H	3.587806	-2.446053	2.107790
H	4.927531	-3.119056	1.117051
H	3.513035	-3.298759	-0.836788
H	2.701019	-4.096477	0.520879
H	-1.873284	3.444016	0.229674
H	-0.986972	5.681552	0.954622
H	-0.702131	5.406086	-0.763267
H	0.624320	5.231738	0.396781
H	0.495957	3.249521	2.175273
H	-0.858551	2.133010	2.099366
H	-1.137285	3.808525	2.592808
H	0.462093	4.191206	-2.257725
H	0.357270	1.433004	-3.596751
H	1.841026	2.209601	-3.066474
H	0.860105	2.952099	-4.353300

H	-1.485495	3.838374	-3.815628
H	-2.041825	3.964990	-2.140479
H	-1.925026	2.378712	-2.921071
H	-0.584056	-3.543465	-2.603223
H	1.383015	-4.443553	-3.773819
H	1.562403	-4.818734	-2.057453
H	2.472431	-3.474234	-2.776626
H	1.334673	-1.267440	-3.362851
H	-0.432733	-1.158622	-3.317530
H	0.356156	-2.208395	-4.507902
H	0.265230	-4.750340	-0.202332
H	-0.854544	-3.131177	2.146176
H	0.847813	-3.532229	1.961058
H	-0.355098	-4.826645	2.158820
H	-2.130867	-5.176102	0.341391
H	-1.986129	-4.392269	-1.231120
H	-2.487545	-3.455390	0.184581
H	-3.599782	1.429934	2.654999
H	-4.803786	3.558637	2.976160
H	-5.636919	4.835814	1.013756
H	-5.253043	3.956760	-1.281728
H	-4.038995	1.834751	-1.608511
H	-5.788364	-0.287084	0.079274
H	-7.325924	-1.470878	-1.443019
H	-6.429804	-2.794254	-3.345385
H	-3.974648	-2.932785	-3.705452
H	-2.427760	-1.765757	-2.154514
H	-4.800615	-1.894750	1.587807
H	-4.849291	-2.840852	3.862937
H	-2.975097	-2.417558	5.437573
H	-1.010923	-1.067077	4.685826
H	-0.932937	-0.166164	2.413715

7. References

S1 M. Green, J. A. K. Howard, J. L. Spencer, F. G. A. Stone, *J. Chem. Soc., Dalton Trans.* 1977, 271−277.

S2 Siemens. SAINT v4 software reference manual. Madison, Wisconsin, USA, Siemens Analytical X-Ray Systems, Inc.; 2014.

S3 G. M. Sheldrick, SHELXTL 2014. Program for structure refinement; Universitat of Göttingen: Göttingen, Germany, 2014.

S4 M. J. Frisch, G. W. Trucks, H. B. Schlegel, G. E. Scuseria, M. A. Robb, J. R. Cheeseman, G. Scalmani, V. Barone, B. Mennucci, G. A. Petersson, H. Nakatsuji, M. Caricato, X. Li, H. P. Hratchian, A. F. Izmaylov, J. Bloino, G. Zheng, J. L. Sonnenberg, M. Hada, M. Ehara, K. Toyota, R. Fukuda, J. Hasegawa, M. Ishida, T. Nakajima, Y. Honda, O. Kitao, H. Nakai, T. Vreven, J. A. Montgomery Jr., J. E. Peralta, F. Ogliaro, M. Bearpark, J. J. Heyd, E. Brothers, K. N. Kudin, V. N. Staroverov, T. Keith, R. Kobayashi, J. Normand, K. Raghavachari, A. Rendell, J. C. Burant, S. S. Iyengar, J. Tomasi, M. Cossi, N. Rega, J. M. Millam, M. Klene, J. E. Knox, J. B. Cross, V. Bakken, C. Adamo, J. Jaramillo, R. Gomperts, R. E. Stratmann, O. Yazyev, A. J. Austin, R. Cammi, C. Pomelli, J. W. Ochterski, R. L. Martin, K. Morokuma, V. G. Zakrzewski, G. A. Voth, P. Salvador, J. J. Dannenberg, S. Dapprich, A. D. Daniels, O. Farkas, J. B. Foresman, J. V. Ortiz, J. Cioslowski, D. J. Fox, Gaussian 09, Revision D.01., Gaussian, Inc., Wallingford, CT 2013.

S5 A. D. Becke, *J. Chem. Phys.* 1993, **98**, 5648−5652

S6 J. P. Perdew, Y. Wang, *Phys. Rev. B: Condens. Matter Mater. Phys.* 1992, **45**, 13244.

S7 X. Cao, M. Dolg, *J. Mol. Struct.: THEOCHEM* 2004, **673**, 203−209

S8 X. Cao, M. Dolg, H. Stoll, *J. Chem. Phys.* 2003, **118**, 487−496.

S9 W. Küchle, M. Dolg, H. Stoll, H. Preuss, *J. Chem. Phys.* 1994, **100**, 7535−7542.

10 T. Leininger, A. Nicklass, W. Küchle, H. Stoll, M. Dolg, A. Bergner, *Chem. Phys. Lett.* 1996, **255**, 274−280.

S11 M. Dolg, U. Wedig, H. Stoll, H. Preuss, *J. Chem. Phys.* 1987, **86**, 866−872.

S12 W. J. Hehre, R. Ditchfield, J. A. Pople, *J. Chem. Phys.* 1972, **56**, 2257-2261.

S13 P. C. Hariharan, J. A. Pople, *Theoret. Chim. Acta* 1973, **28**, 213−222.

S14 H. B. Schlegel, *J. Comput. Chem.* 1982, **3**, 214−218.

S15 A. E. Reed, L. A. Curtiss, F. Weinhold, *Chem. Rev.* 1988, **88**, 899−926.

S16 G. A. Zhurko, Home Page: a set of graphical tools for facilitating working with quantum chemistry computations (http://www.chemcraftprog.com).
